# Research about eye health and eye health services in Pacific Island Countries and Territories: a scoping review

**DOI:** 10.1016/j.lanwpc.2024.101152

**Published:** 2024-07-27

**Authors:** Lisa M. Hamm, Iris Wainiqolo, Nayana Pant, Subash Bhatta, Danielle Petrie-Deely, Pushkar Silwal, Benjamin Zuvani, Ana Patricia Marques, Nimisha Chabba, Laite Tuiloma, Valeria Lopez, Osea Masilaca, Jacqueline Ramke

**Affiliations:** a School of Optometry and Vision Sciences, University of Auckland, New Zealand; b The Fred Hollows Foundation New Zealand, Auckland, New Zealand; c Pacific Eye Institute, Suva, Republic of Fiji; d National Prevention of Blindness Committee, Port Moresby, Papua New Guinea; e The Fred Hollows Foundation New Zealand, Port Moresby, Papua New Guinea; f International Centre for Eye Health, London School of Hygiene & Tropical Medicine, United Kingdom; g Eye Department, Ebeye Hospital, Kwajalein Atoll, Republic of the Marshall Islands; h Fiji National University, Suva, Republic of Fiji

**Keywords:** Oceania, Pacific Island Countries and Territories, Eye health research, Global eye health, Vision impairment

## Abstract

**Background:**

We aimed to summarise the extent and nature of published research about eye health and eye health services in Pacific Island Countries and Territories since 1980.

**Methods:**

We searched Medline, EMBASE, Global Health and Cochrane Library to identify publications about eye health and eye health services in 22 Pacific Island Countries and Territories from 1 January 1980 to 26 January 2024. Study selection and data extraction were conducted by two reviewers independently.

**Findings:**

Of the 1610 publications identified, 180 were included. This research was most commonly conducted in Papua New Guinea (n = 52) or Fiji (n = 33) and focused on diabetic retinopathy (n = 29) or trachoma (n = 18), with few focused on cataract or refractive error. While eye health services research was common in the past, recent research focused on trachoma. The included research was largely undertaken and funded by people and organisations from Australia, Aotearoa New Zealand and the USA, though authors with Pacific affiliations is increasing.

**Interpretation:**

Few countries have up-to-date estimates of the prevalence of vision impairment or service coverage to enable evidence-informed planning. Increased effort is required to strengthen research capability to ensure research priorities in eye health are set by Pacific Peoples.

**Funding:**

The Fred Hollows Foundation New Zealand.

**Research in context**

**Evidence before this study**

We ran a preliminary search on Medline, EMBASE, and Cochrane reviews, using (“Eye health” or “eyecare” or “eye care”) and “Pacific Island∗” as keywords. We limited the search to review articles written since 2000. We found structured reviews asking specific questions about Pacific Islanders living in high-income countries, narrative reviews about the state of eye health in Pacific Island Countries, and a few studies estimating prevalence of common eye conditions in the Pacific region. The reviews about prevalence highlighted the lack of quality data from many Pacific Island Countries. We did not find a comprehensive review summarising research about eye health and eye health services in Pacific Island Countries, which we believe is an important step to setting goals for future research.

**Added value of this study**

We used a structured scoping review to comprehensively map published research about eye health and eye health services in Pacific Island Countries, highlighting key trends from the beginning of 1980 to the end of 2023. By synthesising the characteristics of the 180 included publications, we highlight knowledge gaps. Notably, for refractive error and cataract, there was relatively few estimates of service coverage, and little research on strategies to improve access to and outcomes of services for these conditions. This evidence gap will need to be addressed to enable Pacific Island Countries to be included in monitoring of progress towards ambitious targets set by member states at the World Health Assembly in 2021 for effective service coverage for cataract and refractive error. By summarising the country affiliations of authors and funders, we also illuminate the influence on eye health research of individuals and organisations outside the region, highlighting the need to strengthen research capacity within Pacific Island Countries.

**Implications of all the available evidence**

Pacific Island Countries have grown in their capacity to provide quality eyecare, demonstrated by the establishment of the Pacific Eye Institute in Suva, Republic of Fiji, and the increasing research about eye health and eye health services since 1980. However, the region also faces longstanding and emerging challenges, culminating in financial instability that limits investment in research, including eye health research. Pacific Island Countries benefit from continued support from institutions and funding agencies from high-income countries, but care must be taken to mitigate the known power asymmetries in global health when high-income countries support research in low- and mid-income countries. Continued strengthening of pathways for leadership in eye health and eye health services research within Pacific Island Countries is needed to ensure research priorities in eye health are set by Pacific Peoples.

## Introduction

Eye health services are a vital component of a health system; quality eye care is associated with better overall health outcomes,^1^ and contributes to attainment of several of the Sustainable Development Goals (SDGs).^2^ Substantial progress has been made towards achieving universal access to quality eye health services in the Pacific region over the last few decades. Longstanding collaboration between local, regional, and international initiatives has led to achievements like the establishment of the Pacific Eye Institute in Suva, The Republic of Fiji (hereafter referred to as Fiji), which delivers quality ophthalmic training for regional clinicians.^3^ This combination of Pacific Island institutions, leadership, and expertise with international support for health system strengthening has accelerated the strengthening of eye health services in the Pacific.^3^

However, achievements have been in the context of ongoing challenges. A key challenge is the ability to provide accessible services and maintain supplies and infrastructure across geographically dispersed, and often remote islands.^4^ Further, the ongoing impact of colonisation within an increasingly capitalist global context, combined with the disproportionate impact of climate change in the region,^5^ has contributed to economic instability in many Pacific Island Countries and Territories^6^ (hereafter referred to as Pacific Island Countries). This economic instability has impacted on a wide range of services, including eye health services.^6^, ^7^, ^8^ These challenges have been exacerbated by the COVID-19 pandemic and its ongoing economic, social, and health impacts.^9^

An overview of the extent of blindness and vision impairment in the wider Pacific region published in 2002 highlighted some specific challenges, including the lack of prevalence data and reliance on visiting teams for surgical services.^10^ There has been progress on both of these challenges over the past two decades, particularly in terms of the development of the in-country eye health workforce which has strengthened surgical capacity within the region. Despite prevalence surveys being conducted in some countries, substantial data gaps remain. Indeed, projections from the Vision Loss Expert Group for the region^11^^,^^12^ are made with very few reports from Pacific Island Countries, which understandably creates uncertainty in the estimates generated.

Despite these uncertainties, cataract and uncorrected refractive error are commonly considered to be the leading causes of vision impairment in the region,^10^, ^11^, ^12^ despite both having highly cost-effective treatments.^1^ Indeed, strategies to improve access to good quality services for people with refractive error or cataract were recently identified as the leading *grand challenges* in global eye health,^13^ and ambitious targets to improve effective service coverage for these two conditions were endorsed by member states at the 74th World Health Assembly in 2021.^14^ In response, across the world—including in Pacific Island Countries—more and better evidence is required to inform efforts to increase effective coverage for refractive and error and cataract, and to monitor progress towards these targets.^1^^,^^13^

In addition to cataract and refractive error, diabetic retinopathy is generally considered another important cause of vision impairment in the Pacific given the high prevalence of diabetes in the region.^15^ A further challenge is that vision loss from diabetic retinopathy can generally not be restored, which reinforces the need to implement evidence-informed strategies to ensure effective screening and treatment services are accessible to prevent disease progression.^1^

### Objective

In the context of the evolving achievements and challenges outlined above, it is useful to understand the extent of existing evidence from the region that countries can draw on to strengthen eye health services, as well as identify gaps in the available evidence. Therefore, the objective of this review was to summarise the extent and nature of published research about eye health and eye health services in Pacific Island Countries since 1980. We aimed to map where and on what topics research was conducted as well as who was involved in funding and undertaking the research and to explore trends over time. Given the broad scope, and varied disciplinary approaches of relevant research, we undertook a structured scoping review.^16^^,^^17^

## Methods

### Protocol and registration

We have reported this review according to the relevant items from the scoping review extension of the PRISMA guideline (PRISMA-ScR^18^) (Annex 1). Ethics was not sought as data are in the public domain. The protocol was published to the Open Science Framework in September 2023 (https://osf.io/xkhnp). The protocol was implemented as described; however, our data synthesis plan evolved as we aligned included literature to our aim, as recommended in scoping reviews.^19^ Some of the data extracted to align with WHO Guide for Action tools^20^, ^21^, ^22^ have been summarised in a table detailing included publications (Annex 2) rather than in this main report.

### Eligibility criteria

We framed our eligibility criteria in terms of population, concepts, and study characteristics.^16^ Eligibility criteria are detailed in our protocol (https://osf.io/xkhnp) and briefly summarised here.

#### Population(s)

We included studies conducted in, or about, any of the 22 Pacific Island Countries (as defined by the Pacific Community [www.spc.int]): American Samoa, Cook Islands, Federated states of Micronesia, Fiji, French Polynesia, Guam, Kiribati, Marshall Islands, Nauru, New Caledonia, Niue, Northern Mariana Islands, Palau, Papua New Guinea (PNG), Pitcairn Islands, Samoa, Solomon Islands, Tokelau, Tonga, Tuvalu, Vanuatu, and Wallis & Futuna. To retain focus on the geographic region, we excluded studies that involved Pacific Peoples living in other countries outside the region. We included publications with data from countries outside the Pacific only if data from at least one Pacific Island Country were included and reported separately.

#### Concept

We included studies related to eye health or eye health services. We use broad definitions for both, as articulated for the *Lancet Global Health* Commission on Global Eye Health in 2021.^23^ We use ‘e*ye health’* to mean ‘the state in which vision, ocular health, and functional ability is maximised, thereby contributing to overall health and well-being, social inclusion, and quality of life’.^23^ We use ‘*eye health services’* to refer to ‘all types of interventions that improve eye health, encompassing the spectrum of promotion, prevention, treatment and rehabilitation’.^23^

#### Study characteristics

We included all study types, including case studies and commentaries, if they included data on eye health or eye health services. We excluded conference abstracts, books and academic theses. We did not restrict our search by language. To explore trends over the past four decades, we included research published from 1 January 1980.

### Search strategy and study selection

We developed a search strategy with an experienced information specialist (Annex 3), and completed custom searches in Medline, EMBASE, Global Health and Cochrane Library databases initially on 16 June 2023 and updated on 26 January 2024. Results were uploaded to Covidence software. After study selection, we reviewed the reference lists of all included studies, and added potentially relevant new reports for screening. We also completed a citation search of relevant reviews highlighted in our initial screening.

Two authors independently screened titles and abstracts of all identified reports, with conflicts resolved by discussion. Two authors independently screened the full text of all potentially relevant studies, with conflicts resolved by discussion.

### Data charting

Two researchers independently extracted the pre-specified data items from each included study. This included year of publication, the Pacific Island Country that was the focus of the study, targeted eye condition (with a particular focus on the leading causes of vision impairment globally—refractive error, cataract, glaucoma, macular degeneration and diabetic retinopathy^24^), country affiliation of author, funding source, as well as several data items about topic and methodological approach. Detailed information about data charting is in our protocol (https://osf.io/xkhnp).

### Data synthesis

Extracted data were used to categorise publications in several ways. Data items related to publication topic and methodological approach were used to assign publications as being primarily focused on either.

• Eye health (population-based prevalence/school-based prevalence/service-based report of various outcomes/case study); or
• Eye health services (basic science/pilot or validation/service evaluation or improvement/workforce development/patient perspective/commentary).

The main condition of interest was assigned to each publication (cataract, refractive error, diabetic retinopathy, trachoma, other). Publications that did not target a single eye condition were broken into two categories based on whether the focus was reporting vision impairment (often also detailing the causes) or whether the publication targeted an aspect of eye health services which was not condition-specific.

For each study we reported all unique country affiliations of the authorship team and all reported funders. We pooled federal government contributions from different departments, however, national aid agencies such as NZAID, AusAID, USAID and UKDID, were reported separately.

## Results

**Fig. 1:**
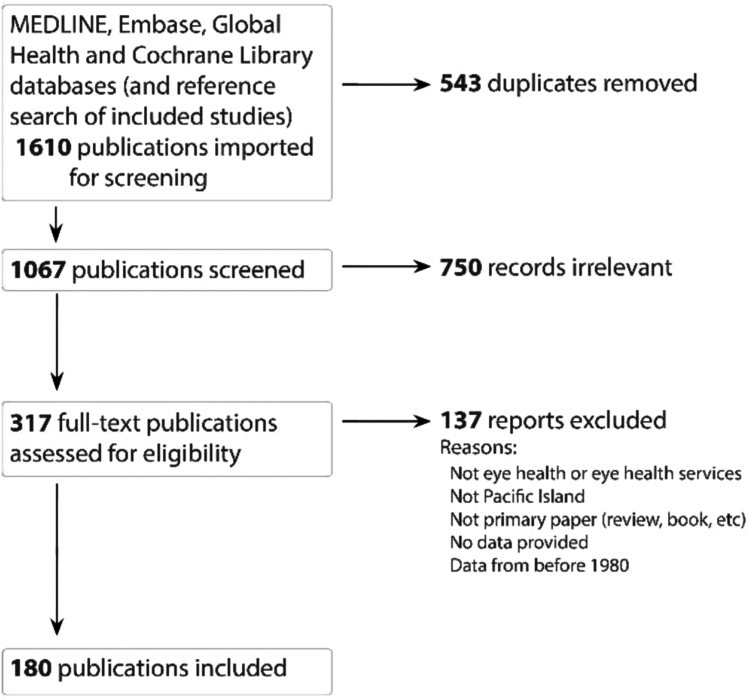
**PRISMA flow diagram to summarise search and selection of included publications**.

Our search identified 1610 publications (1602 from the database searches and 8 from screening reference lists of included publications), leading to 180 publications that met inclusion criteria (Fig. 1); one included study was translated from Japanese, all other included studies were published in English. A summary of all included publications is provided in Annex 2.

### Where was the research conducted?

**Fig. 2:**
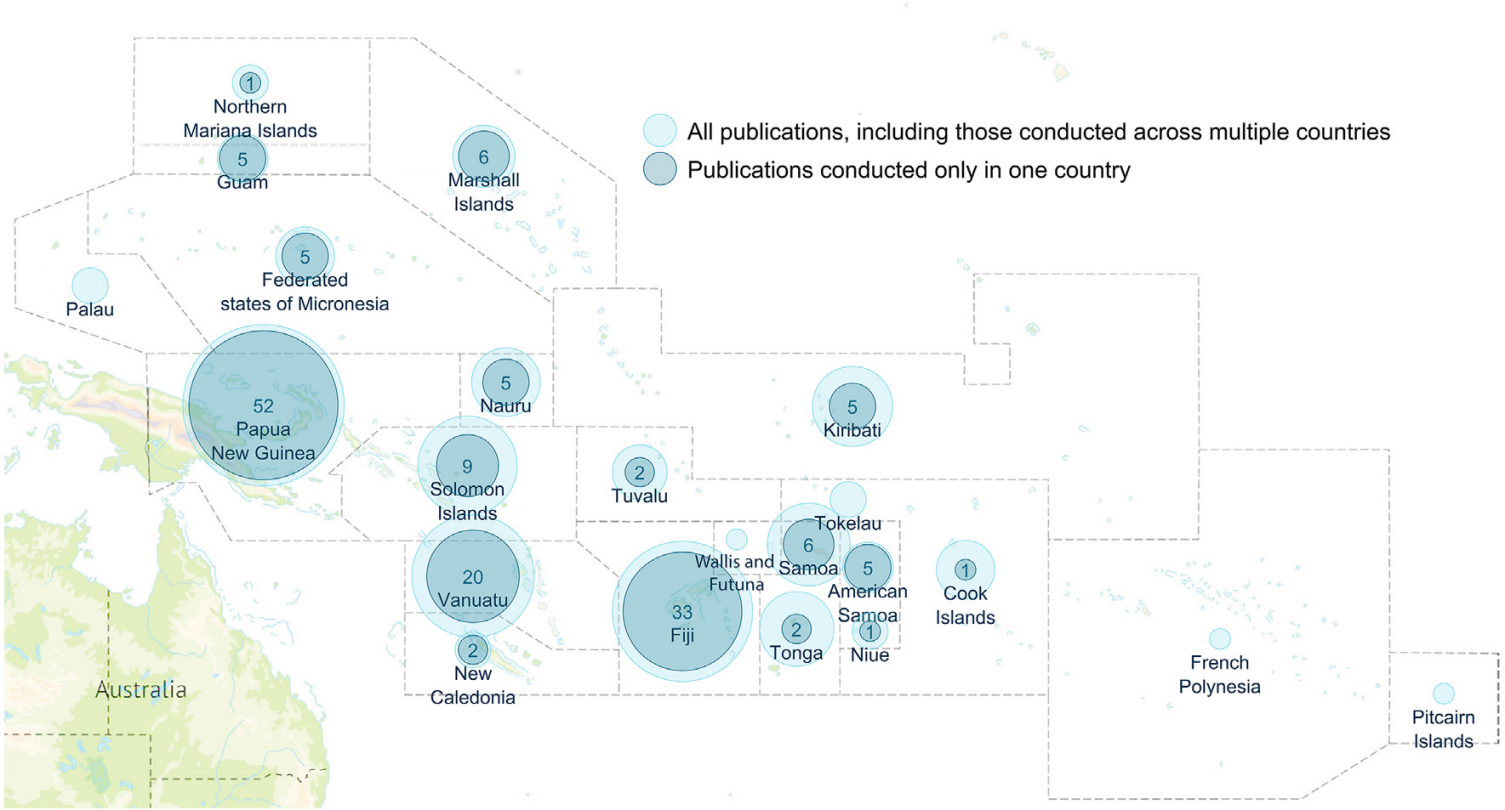
**Map representing number of publications by Pacific Island Country**. Wider, lighter blue dots include multi-country publications (counted multiple times), while darker blue dots indicate publications only about one country. The numbers within the dots indicate the number of publications.

Twenty publications reported results for more than one Pacific Island Country or took a regional approach. Of the 160 publications focused on a single country, these were most commonly conducted in PNG (n = 52, 29%) or Fiji (n = 33, 18%); five of the countries (French Polynesia, Palau, Pitcairn Islands, Tokelau and Wallis and Futuna) had no research outputs (Fig. 2).

### Who conducted the research?

The countries with which author teams most frequently had an affiliation were Australia (n = 65, 36%), Aotearoa New Zealand (n = 52, 29%), the United States of America (n = 50, 28%), PNG (n = 46, 26%), United Kingdom (n = 33, 18%) and Fiji (n = 30, 17%). Most publications (n = 125, 69%) had at least one author affiliated with a Pacific Island Country, but only 27 (15%) were conducted entirely by authors affiliated with institutions in Pacific Island Countries.

Almost half of the included publications did not report specific funding (n = 77, 43%). The most frequent funder was The Fred Hollows Foundation New Zealand (n = 21, 12%), followed by The Fred Hollows Foundation Australia (n = 16, 9%) and governmental aid agencies from Aotearoa New Zealand (NZAID, n = 16, 9%), Australia (AusAID, n = 15, 8%) and the United States of America (USAID, n = 12, 7%).

Some included publications discussed the merits and limitations of external support provided to the region. A small group of early studies by visiting clinicians highlighted adventure and travel anecdotes, including a study using ‘sight-seeing’ in the title, which sparked debate in the literature about motivation for engagement. Some included publications critiqued record keeping and follow up of visiting clinical teams, the impact of externally funded, free-for-user cataract surgery on the sustainability of in-country eye health services, and the appropriateness of donated glasses for the correction of refractive error.

### What was the research about?

The specific conditions most commonly focused on were diabetic retinopathy (n = 29, 16%), trachoma (n = 18, 10%), refractive error (n = 14, 8%), and cataract (n = 9, 5%). There were only two studies targeting glaucoma, and no studies specifically about age-related macular degeneration. Twenty-five studies were about vision impairment more generally (more detail in Annex 5).

**Fig. 3:**
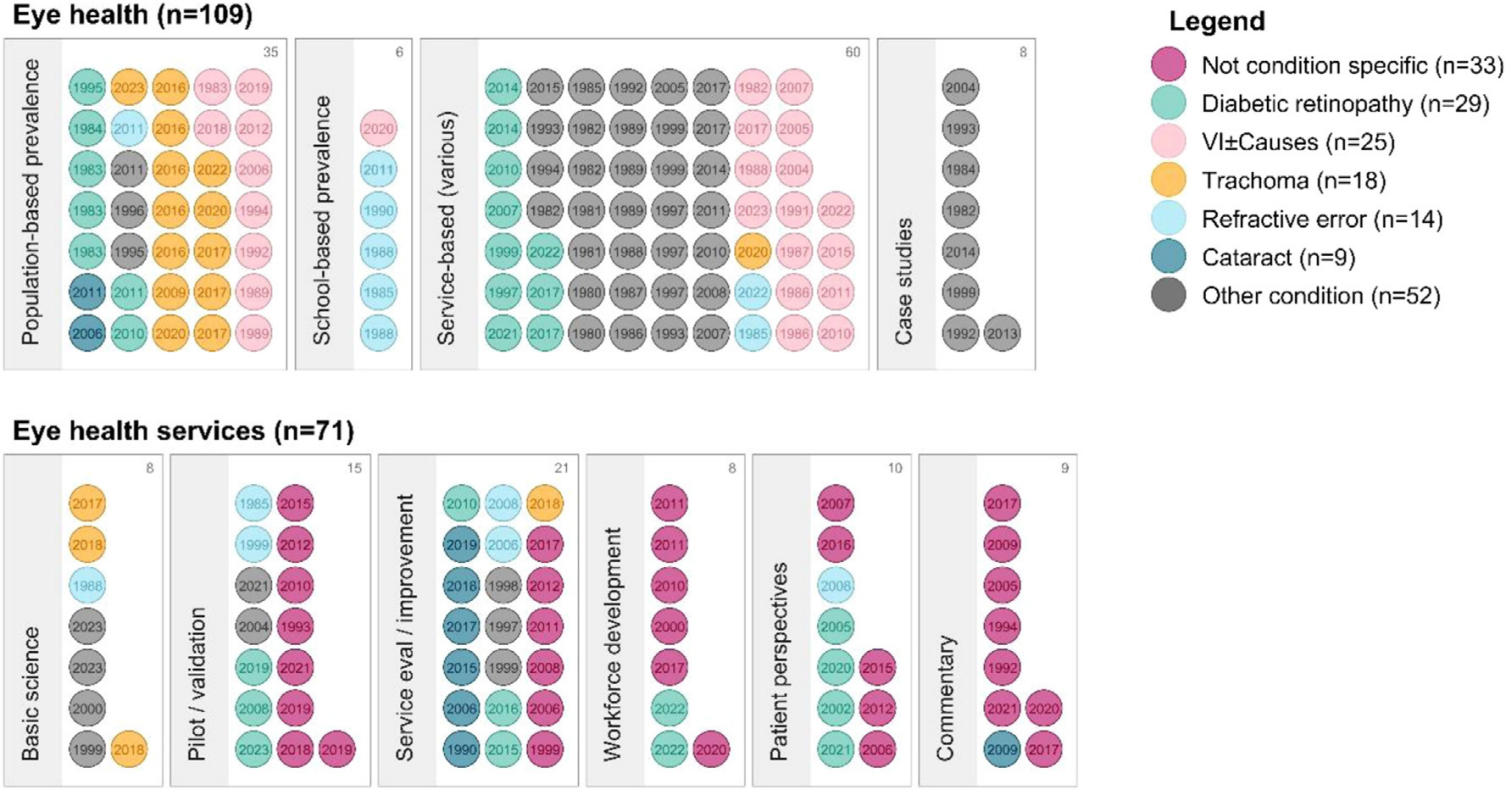
**Overview of study types and topics included in eye health and eye health services publications in Pacific Island Countries from 1980 to 1923**. Each publication is represented by a dot including the year of publication. Colour represents the targeted eye condition, outlined in the legend; VI, vision impairment.

Most of the included publications presented data on eye health of populations (n = 109, 61%), with the remaining publications presenting data primarily about eye health services (n = 71, 39%). Fig. 3 summarises included publications by targeted eye condition and topic focus.

Over half of the publications that focused on eye health sampled people attending services (n = 60), and an additional eight were case studies. These publications included retrospective reviews of medical records and reports from outreach activities and international visits, and often focused on less researched conditions (including conjunctivitis, vitamin A deficiency, ocular trauma and growths). Many of these publications included an estimate of how common eye conditions were among people attending services (sometimes referred to as an estimate of prevalence).

Thirty-five publications estimated prevalence of one or more eye condition with population-based samples, most commonly focused on trachoma (n = 13), vision impairment (n = 9), or diabetic retinopathy (n = 7). In population-based prevalence studies about vision impairment that reported the causes of impairment, cataract was most commonly the leading cause of blindness, and uncorrected refractive error was the leading cause of vision impairment. An additional six publications sampled school children, all but one was investigating the prevalence of refractive error.

The 71 publications focused on eye health services were varied; 21 aimed to evaluate or improve the quality of current services, with six of these focused on cataract surgery. Ten publications described patient perspectives on current practice; gathering insights with questionnaires and interviews, most commonly about prevention of diabetic retinopathy (n = 4). Nine publications broadly summarised existing service provision through expert commentary, and another eight described the strengthening of the eye care workforce (these tended to have a broad focus—only three of the combined 17 targeted a specific condition).

We classified twenty-three publications as aiming to strengthen future innovation within eye health services. Eight of these publications used basic science approaches (most commonly genetic analysis) to understand disease etiology. This work focused on biomarkers for trachoma, but also included studies investigating achromatopsia and Leber's congenital amaurosis. An additional 15 studies aimed to develop, pilot or validate specific tools, including portable screening tools for diabetic retinopathy (n = 3), glaucoma (n = 1) or refractive error (n = 2), as well as standardised questionnaires to understand the impact of low vision (n = 6).

### What were key trends since 1980?

**Fig. 4:**
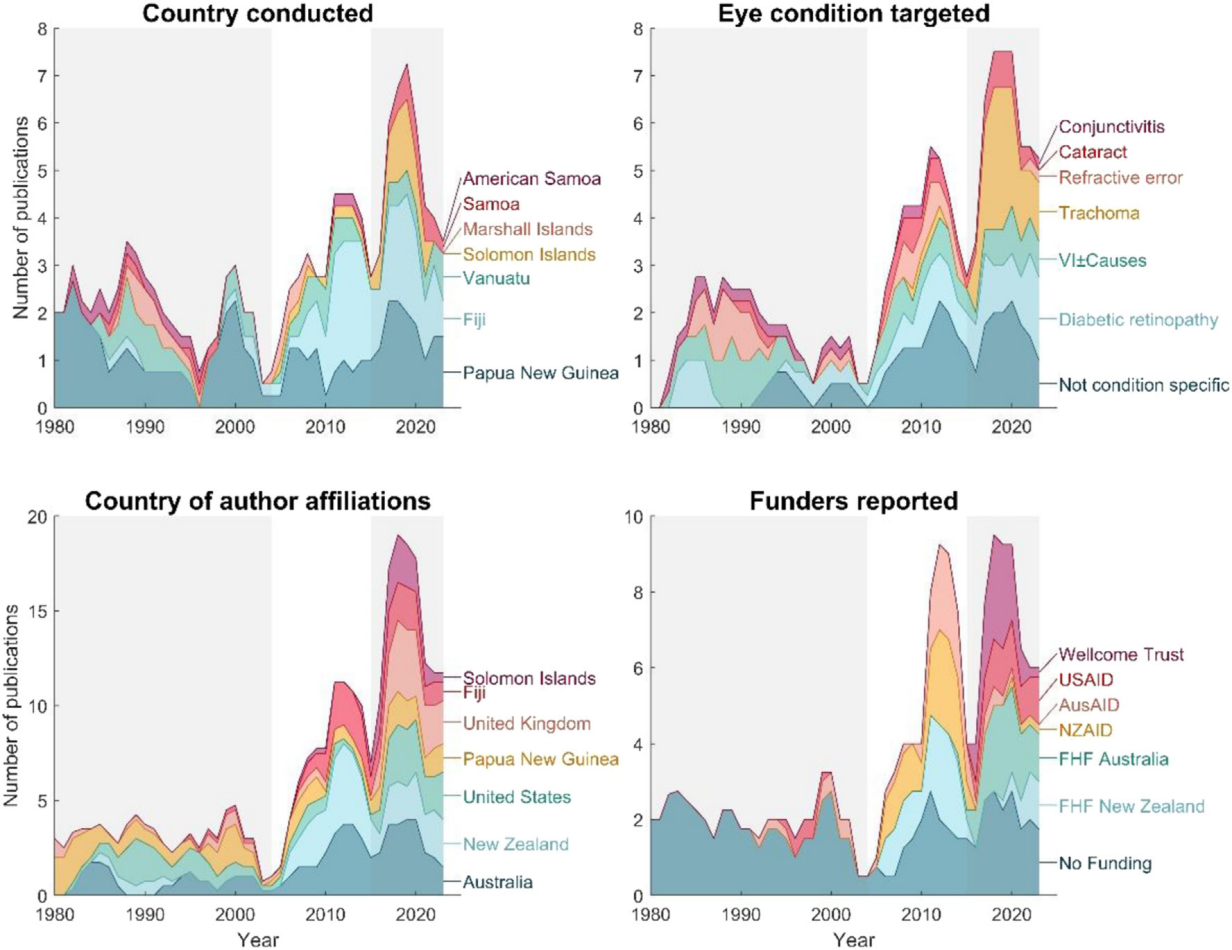
**Characteristics of publications on eye health or eye health services in Pacific Island Countries from 1980 to 2023 in terms of a) the country of research b) the eye condition targeted by the research c) the countries with which authors had affiliations and d) the funders reported**. The number of publications is a rolling average, with data for each year representing a mean of the previous three years. Country conducted and eye condition targeted are limited to one per publication, while funders reported, and country of author affiliations often include more than one per publication. Only the top seven most frequently reported options are plotted. Divisions between shaded areas emphasise dips in research outputs, highlighting the three phases of research described in the text. VI, vision impairment; FHF, The Fred Hollows Foundation.

Across our study period, the number of reports published increased at a rate of about 4% per year. From 1980 to 2023, author teams increasingly included at least one member with an affiliation in a Pacific Island Country, whereas research done exclusively by authors with Pacific Island Country affiliation decreased. The focus of research has changed over time, appearing to be separated into phases highlighted by dips and peaks in research outputs (Fig. 4).

We grouped the study period into three phases to describe the trends. In the first phase from the 1980s to the early 2000s, research was largely conducted in PNG (and to a lesser extent in Vanuatu) mainly focused on overall vision impairment and refractive error. This phase of research also included the publication of many descriptions of patients accessing care for rare eye conditions, and work conducted in remote islands. These publications did not tend to report funding, and had small authorship teams, mostly affiliated with institutions in PNG, the United States of America, and Australia.

In the second phase of research, from 2004 to 2015, more publications were focused on Fiji, and the focus of the research shifted from describing eye health to evaluating and/or improving eye health services in ways that were not condition specific (focusing on topics such as workforce development and local infrastructure). Much of the research in this phase was supported by funding from AusAID, NZAID and The Fred Hollows Foundation New Zealand, with author affiliation increasingly within Aotearoa New Zealand and Fiji.

The most recent phase saw an emphasis on trachoma research with large, international authorship teams, reporting integrated funding from global trachoma initiatives. During this phase, research continued in PNG, Fiji, and Vanuatu, and increased in Solomon Islands. This most recent phase appears to have peaked around 2020, with a decline in output in subsequent years.

**Fig. 5:**
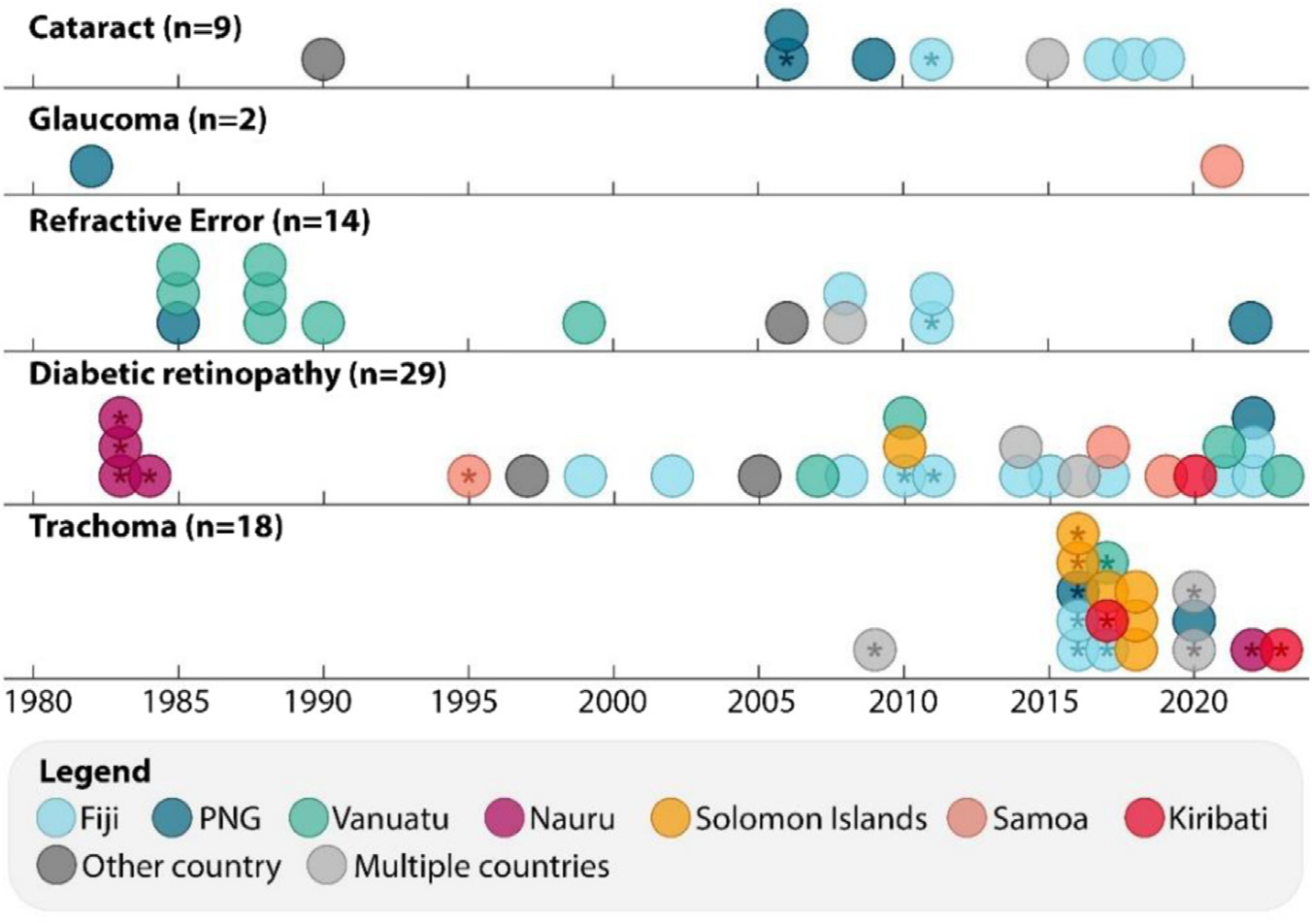
**Summary of publications in Pacific Island Countries from 1980 to 2023 that focused on the leading causes of vision loss globally.** Each study is represented by a dot, with colour indicating the country of research. Eye conditions are sorted by most common cause of blindness globally in 2020,^24^ top to bottom (no publications focused on age-related macular degeneration); trachoma was added because it was a focus of research in the region. Asterisks denote population-based prevalence studies.

The trajectory of research about specific eye conditions in Pacific Island Countries does not reflect the leading causes of blindness globally (Fig. 5). Specifically, research about cataract, glaucoma and refractive error are not increasing. There are few studies about cataract (almost all in PNG and Fiji), only two publications focused on glaucoma (one in the 1980s), and most of the publications about refractive error were interested in school-children in the 1980s. Diabetic retinopathy and trachoma have been the clear focus of recent research, with diabetic retinopathy research sustained over time, while trachoma research increased dramatically after 2015. The only population-based prevalence studies conducted in the last decade were about trachoma, and vision impairment more generally. PNG and Fiji are the only countries with estimates of service coverage for cataract or refractive error. In terms of studies that could contribute knowledge on how to improve service coverage, six studies focused on monitoring or improving quality of cataract outcomes (primarily in Fiji) and two studies focused on improvement of refractive error outcomes.

## Discussion

### Summary of findings

We took a very inclusive approach with the selection criteria for this review, to capture the full extent of published literature about eye health and eye health services in Pacific Island Countries across the more than four decades since 1980. Half of the included publications were focused on PNG or Fiji, while five of our 22 countries had no publications. Few countries have up-to-date estimates of prevalence of vision impairment or service coverage to inform planning. There was a time when eye health service research was prioritised, but this has dwindled in the last decade. Author affiliations and funding sources were more commonly from Australia, Aotearoa New Zealand and the United States of America than any Pacific Island Country, though the inclusion of authors with affiliations in Pacific Island Countries is increasing.

The publications included sit across a spectrum of research methodologies and robustness. While some of the identified publications follow ‘gold standard’ approaches, almost 40% of included publications simply described the eye health of patients engaging with clinical activities. These clinic reports represent the bulk of research driven primarily by Pacific Island researchers, and while they may not represent the most robust research methods, these reports are critical for growth in the field in two ways. Firstly, these reports generate useful information in the absence of any other description of eye health and eye health services in many Pacific Island Country settings. Secondly, the reports represent the development of research capacity from within Pacific Island Countries, an important step towards the development of Pacific eye health leadership.^3^^,^^25^

Although the increase in publications over this period is similar to that in ophthalmic journals overall (between 3 and 4% annual increase^26^), the trends in research suggest a mismatch between research priorities and regional and global priorities. Specifically, research about many of the leading causes of blindness and vision impairment globally,^24^ and in the Pacific,^12^ including cataract and refractive error, was scarce. While service coverage estimates were available for cataract in PNG and Fiji, and refractive error in Fiji (from studies that pre-dated the effective coverage definitions^27^^,^^28^), additional studies will be required for Pacific Island Countries to feed into the reporting against WHO targets for effective coverage due in 2030.^29^ Fortunately, this evidence gap is being addressed in some countries—a national Rapid Assessment of Avoidable Blindness was completed in Vanuatu in late 2023, and similar studies are planned for Samoa and Fiji in 2024.

The sustained interest in diabetic retinopathy across our study period is encouraging given the high prevalence of diabetes,^15^ and diabetes complications^30^ in the region. The extensive work on trachoma in recent years is perhaps unexpected, as it was suspected to be low in the region as of 2015.^12^ However, a 2018 review highlighted the lack of quality prevalence data,^31^ a gap which was actively filled.

The phases of research outputs about eye health and eye health services correspond with a series of external factors. For example, the establishment of the Pacific Eye Institute in Fiji in 2006^3^ with concurrent funding from Aotearoa New Zealand and Australia for eye health services research, and the Global Trachoma Mapping Project, which launched in 2012 and provided extensive funding for trachoma mapping projects.^32^ The timing of these initiatives in relation to subsequent trends in publications highlights the key role of external funding in research priority setting.

### Implications for policy and practice

Our intent is that this review (and associated condition and country summaries in Annex 4 and Annex 5) can aid researchers in Pacific Island Countries and their partners to identify priority research questions. However, the extent of external funding and internationally affiliated research revealed in this review highlights the need for external actors to reflect on their contribution to the regional research ecosystem.

Unbalanced research collaboration between high- and low- or middle-income countries is common in the field of global health; training and funding opportunities are disproportionately accessible for people in high-income countries.^33^ Although touted as mutually beneficial (or philanthropic), when people from high-income countries are involved in research in low- or middle-income countries, hierarchical power dynamics can undermine initiatives and priorities within the low- or middle-income countries.^34^^,^^35^ An important component of countering these power dynamics is strengthening research leadership in low- and middle-income countries.

This review highlights some barriers to engagement with research for eye care providers in Pacific Island Countries which inhibit the strengthening of in-country research leadership. Included papers about workforce development and infrastructure highlight high clinical demands on staff. The experience of collaboratively compiling this review also revealed that motivation from collaborators within Pacific Island Countries to shape research is high, but so are practical barriers. Mid-level, highly motivated eye health professionals are often tasked with staffing busy clinics and running outreach programs while working in often challenging contexts, including infrastructure and resource constraints. Staff are facing these challenges while navigating increasingly frequent weather events, which disrupt plans and communication systems, as well as pose an active threat to families and homes. To achieve sustained development of research capacity in the region, strategies must be developed to ensure there are pathways for aspiring researchers to be trained and funded without compromising services.

Any efforts to foster eye health research leadership within Pacific Island Countries face considerable financial constraints. Given the pressing needs in the region in the face of climate change and economic instability,^5^^,^^7^^,^^8^ exacerbated by the COVID-19 pandemic,^9^ allocating in-country resources to research on eye health and eye health services is a challenge. It is imperative, therefore, that the investments that are made are strategic. Research priorities need to contribute to improving services that will lead to the highest impact. This needs to be a continued collaborative venture, building from the longstanding synergy between Pacific Island and international initiatives.^3^ and incorporating lessons learned about power asymmetries inherent in global health research.^34^^,^^35^ To align research projects with in-country priorities and to contribute to sustainable research in the region, we call for researchers and funders from outside the region to thoughtfully embed the strengthening of Pacific Island researchers within all future research initiatives.

### Limitations

Our review must be interpreted in the context of several limitations. First, limiting the review to published reports was important to understand the state of research, but it means relevant information within grey literature may have been missed. Second, the broad scope of the review necessarily limited the level of detail we were able to provide, though we believe this approach is in keeping with the remit of a scoping review.^18^ For example, within publications focused on vision impairment broadly, we did not collate information on the range of eye conditions reported as common causes of vision impairment. Even among population-based prevalence studies, inconsistent categorisation prevented meaningful summary by eye condition. Third, we used some simplifications which may mask realities. For example, funding sources were only extracted if specifically listed as contributing financially, meaning organisations referred to vaguely were not captured in our analysis. Finally, while not a limitation of our approach *per se*, we acknowledge that the country of author affiliation does not necessarily reflect whether or not the person is from a Pacific Island Country. While we are aware of a small number of Pacific Island authors with an affiliation outside the Pacific, it is likely that overall we have overestimated involvement of Pacific Island researchers due to temporary residents using a Pacific Island affiliation when publishing research (often in PNG). Similarly, reporting all authorships rather than first and last authorships meant we cannot reflect on the role of researchers from Pacific Island Countries, which may have highlighted further imbalances for these researchers, as recently identified from health research in Sub-Saharan Africa.^36^ We hope the value of this first high-level summary of the eye health in the region overrides the limitations inherent in mapping such a broad and diverse field, and acts as a launching point for future research.

## Conclusion

More research is needed so that Pacific Island Countries can take an evidence-informed approach to advancing national and regional eye health priorities. In particular, there are few estimates of service coverage for refractive error and cataract, and limited research on strategies to improve access to and outcomes of services for these conditions. Addressing this evidence gap is essential for Pacific Island Countries to be included in monitoring progress towards the ambitious targets set by member states at the World Health Assembly in 2021. In the process of enhancing research in the region, care must be taken to channel funding and research capacity strengthening to maximise impact, and to increase the decision-making capability of Pacific Island Countries to navigate and own their research journey.

## Reflexivity statement

This review is intended to provide a resource for priority setting within Pacific Island Countries and at the regional level. A key goal of the research process was reciprocal capacity strengthening, where screening and data extraction were done by collaborating authors across the Pacific region, facilitated by workshops to promote shared learning, collaboration, and dissemination of gathered information. Specifically, several senior researchers from Pacific Island Countries participated in drafting the protocol. We recruited eye health workers from Pacific Island Countries interested in learning more about research to participate in the review process. Two workshops were run to support early career researchers, and those new to structured reviews, to build analytical skills, and inform research goals. Everyone who collaborated in screening and data extraction were invited to contribute to developing the manuscript and being an author (9 of 13 females). Those who completed screening but not data extraction were formally acknowledged. Using online software (Covidence) allowed all collaborators to access raw data, reflect on content and write summaries of included papers, forming the basis of data synthesis. All members of the authorship team were provided with a summary as data extraction neared completion, to generate feedback. The research shared in this succinct publication, is also available in a comprehensive report detailing data by country and condition (Annex 4 and Annex 5), allowing independent exploration and interpretation of the data.

## Ethics and dissemination

Ethical approval has not been sought as the included literature is publicly available.

## Contributors

LH: methodology, investigation [screening, data extraction, data synthesis], data curation, formal analysis, visualisation, project administration, writing—original draft; IW: investigation [screening, data extraction, data synthesis–review], writing—review & editing; NP: investigation [screening, data extraction, data synthesis–review], writing—review & editing; SB: investigation [screening, data extraction, data synthesis–review], writing—review & editing; DP: investigation [screening, data extraction, data synthesis–review], writing—review & editing; PS: investigation [screening, data extraction, data synthesis–review], writing—review & editing; BZ: investigation [screening, data extraction, data synthesis–review], writing—review & editing; APM: investigation [screening, data extraction, data synthesis–review], writing—review & editing; NC: investigation [screening, data extraction, data synthesis–review], writing—review & editing; LT: investigation [screening, data extraction, data synthesis–review], writing—review & editing; VL: investigation [screening, data extraction, data synthesis–review], writing—review & editing; OM: investigation [screening, data extraction, data synthesis–review], writing—review & editing; JR: conceptualisation, methodology, investigation [screening, data extraction, data synthesis], supervision, writing—original draft.

## Editor note

The Lancet Group takes a neutral position with respect to territorial claims in published maps and institutional affiliations.

## Declaration of interests

JR, LH PS and APM report a grant from The Fred Hollows Foundation New Zealand to support the conduct of this study. No other author has a conflict to disclose.

## Appendix A Supplementary data

ANNEX 1-3

ANNEX 4_Hamm et al Overview by Country

ANNEX 5_Hamm et al By Condition
