# Supplementary material for: Research about eye health and eye health services in Pacific Island Countries and Territories: a scoping review
Source: Lancet Reg Health West Pac. 2024 Jul 27;50:101152. doi: 10.1016/j.lanwpc.2024.101152 (PMC11332796; doi:10.1016/j.lanwpc.2024.101152)
Supplement: ANNEX 1-3 [file mmc1.docx]

Annex for Research about eye health and eye health services in Pacific Island Countries and Territories: a scoping review

Annex 1: PRISMA-Scr checklist page 2

Annex 2: Summary of included studies page 4

Annex 3: Search Strategy page 16

Annex 4 and 5 available separately

# Annex 1: PRISMA-Scr

| **SECTION** | **ITEM** | **PRISMA-ScR CHECKLIST ITEM** | **PAGE #** |
| --- | --- | --- | --- |
| **TITLE** | | | |
| Title | 1 | Identify the report as a scoping review. | 1 |
| **ABSTRACT** | | | |
| Structured summary | 2 | Provide a structured summary that includes (as applicable): background, objectives, eligibility criteria, sources of evidence, charting methods, results, and conclusions that relate to the review questions and objectives. | 2 |
| **INTRODUCTION** | | | |
| Rationale | 3 | Describe the rationale for the review in the context of what is already known. Explain why the review questions/objectives lend themselves to a scoping review approach. | 4 |
| Objectives | 4 | Provide an explicit statement of the questions and objectives being addressed with reference to their key elements or other relevant key elements used to conceptualize the review questions and/or objectives. | 4 |
| **METHODS** | | | |
| Protocol and registration | 5 | Indicate whether a review protocol exists; state if and where it can be accessed; and if available, provide registration information, including the registration number. | 5 |
| Eligibility criteria | 6 | Specify characteristics of the sources of evidence used as eligibility criteria (e.g., years considered, language, and publication status), and provide a rationale. | 5 |
| Information sources* | 7 | Describe all information sources in the search (e.g., databases with dates of coverage and contact with authors to identify additional sources), as well as the date the most recent search was executed. | 5 |
| Search | 8 | Present the full electronic search strategy for at least 1 database, including any limits used, such that it could be repeated. | Annex3 |
| Selection of sources of evidence† | 9 | State the process for selecting sources of evidence (i.e., screening and eligibility) included in the scoping review. | 5,6 |
| Data charting process‡ | 10 | Describe the methods of charting data from the included sources of evidence (e.g., calibrated forms or forms that have been tested by the team before their use, and whether data charting was done independently or in duplicate) and any processes for obtaining and confirming data from investigators. | 6 |
| Data items | 11 | List and define all variables for which data were sought and any assumptions and simplifications made. | 6 |
| Critical appraisal | 12 | If done, provide a rationale for conducting a critical appraisal of included sources of evidence; describe the methods used and how this information was used | na |
| Synthesis of results | 13 | Describe the methods of handling and summarizing the data that were charted. | 6 |
| **RESULTS** | | | |
| Selection of sources of evidence | 14 | Give numbers of sources of evidence screened, assessed for eligibility, and included in the review, with reasons for exclusions at each stage, ideally using a flow diagram. | Fig1 |
| Characteristics of sources of evidence | 15 | For each source of evidence, present characteristics for which data were charted and provide the citations. | Annex2 |
| Critical appraisal | 16 | If done, present data on critical appraisal of included sources of evidence (see item 12). | na |
| Results of individual sources of evidence | 17 | For each included source of evidence, present the relevant data that were charted that relate to the review questions and objectives. | Annex2 |
| Synthesis of results | 18 | Summarize and/or present the charting results as they relate to the review questions and objectives. | 6-8.Fig2-5 |
| **DISCUSSION** | | | |
| Summary of evidence | 19 | Summarize the main results (including an overview of concepts, themes, and types of evidence available), link to the review questions and objectives, and consider the relevance to key groups. | 8,9 |
| Limitations | 20 | Discuss the limitations of the scoping review process. | 10 |
| Conclusions | 21 | Provide a general interpretation of the results with respect to the review questions and objectives, as well as potential implications and/or next steps. | 9,10 |
| **FUNDING** | | | |
| Funding | 22 | Describe sources of funding for the included sources of evidence, as well as sources of funding for the scoping review. Describe the role of the funders of the scoping review. | 2,11 |

# Annex 2: Summary of Included Studies

# Publications primarily about Eye Health

| Study ID | Title | Focus | Aim | Eye condition | Authors affiliation | Funding |
| --- | --- | --- | --- | --- | --- | --- |
| 'Naraqi 1992' | 'Quinine blindness.' | 'Case study' | 'To present a case report on quinine blindness and review the management' | 'Quinine blindness' | 'PNG' | 'No Funding' |
| 'Cheng 1999' | 'Superior orbital fissure syndrome in a latent type 2 diabetic patient' | 'Case study' | 'To describe a case of a complete superior orbital fissure syndrome including the optic nerve' | 'Cranial nerve palsy' | 'PNG' | 'No Funding' |
| 'Grogan 2014' | 'Sudden onset Oculo-cardiac Reflex post-traumatic eye injury in PNG: a case study and discussion' | 'Case study' | 'To present a case developing oculocardiac reflex leading to bradycardia due to trauma and its management' | 'Ocular trauma' | 'PNG' | 'No Funding' |
| 'Scrimgeour 1982' | 'A probable case of ocular angiostrongyliasis in New Britain, Papua New Guinea.' | 'Case study' | 'To describe a case of an intraocular nematode infestation' | 'Ocular angiostrongyliasis' | 'PNG' | 'No Funding' |
| 'Scrimgeour 1984' | 'Oculopharyngeal and distal myopathy: a case study from Papua New Guinea.' | 'Case study' | 'To describe a case of ptosis (alongside wider issues)' | 'Ptosis' | 'PNG, Australia' | 'No Funding' |
| 'Hanlon 1993' | 'An unusual retinal pigment epitheliopathy endemic to the island of Guam.' | 'Case study' | 'To present three case reports as samples of the clinical presentation of an unusual retinal pigment epitheliopathy resembling ophthalmomyiasis interna posterior' | 'Retinopathy (ALS-PDC)' | 'Guam' | 'No Funding' |
| 'Tay 2004' | 'Goldenhar syndrome: a case from Papua New Guinea.' | 'Case study' | 'To report a case of Goldenhar Syndrome in Papua new guinea' | 'Tumors/growths' | 'Australia, PNG' | 'No Funding' |
| 'Aiyub 2013' | 'Congenital orbital teratoma.' | 'Case study' | 'To describe the successful use of demis fat graft in socket reconstruction following lid-sparing exenteration for congenital orbital teratoma.' | 'Tumors/growths' | 'Fiji, Australia' | 'No Funding' |
| 'Goodhew 2023' | 'Changes in trachoma indicators in Kiribati with two rounds of azithromycin mass drug administration, measured in serial population-based surveys.' | 'Population-based prevalence' | 'To compare different indicators for the eye disease trachoma before and after two annual rounds of mass azithromycin treatment of affected communities of Kiribati ' | 'Trachoma' | 'Kiribati, UK, USA, Australia, Nigeria, Switzerland' | 'USAID, Intl Trachoma Initiative, Sightsavers, Act to END, Pfizer, FHFAus, Queen Elizabeth Trust' |
| 'Schaumberg 1995' | 'Vitamin A deficiency in the South Pacific' | 'Population-based prevalence' | 'To determine the extent of Vitamin A deficiency in the South Pacific region' | 'Vitamin A deficiency' | 'USA, Fiji' | 'USAID' |
| 'Burnett 2018' | 'Rapid assessment of avoidable blindness and diabetic retinopathy in people aged 50 years and older in the National Capital District of Papua New Guinea' | 'Population-based prevalence' | 'To conduct an assessment of avoidable blindness, diabetes mellitus and diabetic retinopathy (DR) in adults aged 50 years and older in the National Capital District (NCD) region of Papua New Guinea (PNG)' | 'VI±Causes' | 'Australia, PNG, NZ, UK, Netherlands' | 'FHFAus' |
| 'Butcher 2020' | 'Conjunctival scarring, corneal pannus and Herbert's pits in adolescent children in trachoma-endemic populations of the Solomon Islands and Vanuatu' | 'Population-based prevalence' | 'To determine the prevalence of pannus, HPs, and conjunctival scarring in children living in villages in which a high proportion of 1–9-year-olds previously had TF.' | 'Trachoma' | 'UK, Solomon Islands, Vanuatu, USA, Australia, Switzerland' | 'FHFAus, Queen Elizabeth Trust, Intl Trachoma Initiative, Pfizer' |
| 'Heriot 1983' | 'Diabetic retinopathy in a natural population.' | 'Population-based prevalence' | 'To understand prevalence of DR (population-based sample)' | 'Diabetic retinopathy' | 'Australia' | 'No Funding' |
| 'King 1983' | 'Characteristics associated with diabetic retinopathy in Nauruans.' | 'Population-based prevalence' | 'To determin the prevalence and characteristics of diabetic retinopathy (population-based sample)' | 'Diabetic retinopathy' | 'Australia' | 'WHO, NIH' |
| 'King 1983' | 'Diabetic retinopathy in Nauruans.' | 'Population-based prevalence' | 'To establish the impact of hyperglycemia on retinopathy in the responders to a diabetes survey in Nauru (population based sample)' | 'Diabetic retinopathy' | 'Australia, New Caledonia' | 'No Funding' |
| 'Heriot 1983' | 'Ophthalmic findings among one thousand inhabitants of Rarotonga, Cook Islands.' | 'Population-based prevalence' | 'To establish the prevalence of diabetes and its vascular complications among the Cook Islanders.' | 'VI±Causes' | 'Australia, New Caledonia' | 'No Funding' |
| 'Zimmet 1984' | 'The high prevalence of diabetes mellitus, impaired glucose tolerance and diabetic retinopathy in Nauru--the 1982 survey.' | 'Population-based prevalence' | 'To determine the prevalence of Diabetes mellitus , impaired glucose tolerance and diabetic retinopathy in Nauru.' | 'Diabetic retinopathy' | 'Australia, Nauru' | 'NIH' |
| 'Wessels 1989' | 'Project Canvas-Back in the Marshall Islands.' | 'Population-based prevalence' | 'To determine the prevalence of eye problems in Wotje, Marshall Islands. ' | 'VI±Causes' | 'USA' | 'Adventist International Eye Society, Loma Linda University' |
| 'Barker 1989' | 'Health and functional status of the elderly in a Polynesian population.' | 'Population-based prevalence' | 'To determine the health status and functional limitations of Niuean elderly (population-wide sample)' | 'VI±Causes' | 'New Zealand' | 'Govt of NZ' |
| 'Newland 1992' | 'Epidemiology of blindness and visual impairment in Vanuatu.' | 'Population-based prevalence' | 'To estimate the prevalence and causes of blindness among the Vanuatu population' | 'VI±Causes' | 'Australia, Vanuatu, Japan' | 'AusAID, ASPECT' |
| 'Newland 1994' | 'Epidemiology of blindness and visual impairment in the kingdom of Tonga.' | 'Population-based prevalence' | 'To obtain an accurate estimate of blindness and its causes in Tonga (population-based sample)' | 'VI±Causes' | 'Australia, Tonga' | 'ASPECT, Australian International Development Assistance Bureau' |
| 'Collins 1995' | 'High prevalence of diabetic retinopathy and nephropathy in Polynesians of Western Samoa.' | 'Population-based prevalence' | 'To determine the prevalence of diabetic retinopathy and nephropathy and to define associated risk factors in Polynesian Western Samoans with non-insulin-dependent diabetes mellitus (NIDDM) or impaired glucose tolerance (IGT).' | 'Diabetic retinopathy' | 'Australia, Samoa' | 'National Inst of Diabetes, Govt of Western Samoa, Intl Diabetes Inst, WHO (Western Pacific Region), Menzies Centre, Baker Medical Research Inst' |
| 'Schaumberg 1996' | 'Risk factors for xerophthalmia in the Republic of Kiribati.' | 'Population-based prevalence' | 'To identify risk factors for xerophthalmia in the Republic of Kiribati' | 'Vitamin A deficiency' | 'USA, Kiribati' | 'Govt of Kiribati, Helen Keller International, Johns Hopkins, Peoples of the South Pacific, USAID' |
| 'Garap 2006' | 'Blindness and vision impairment in the elderly of Papua New Guinea.' | 'Population-based prevalence' | 'To estimate the magnitude and causes of blindness and vision impairment in Papua New Guinea for service delivery planning and ophthalmic education development.' | 'VI±Causes' | 'PNG, India, NZ' | 'NZAID, FHFNZ' |
| 'Garap 2006' | 'Cataract and its surgery in Papua New Guinea.' | 'Population-based prevalence' | 'To determine the prevalence of visually significant cataract, unoperated blinding cataract, and cataract surgery for those aged 50 years and over in Papua New Guinea. Also, to determine the characteristics, rate, coverage and outcome of cataract surgery, and barriers to its uptake.' | 'Cataract' | 'PNG, India, NZ' | 'NZAID, FHFNZ' |
| 'Mathew 2009' | 'Trachoma in the Pacific Islands: evidence from Trachoma Rapid Assessment.' | 'Population-based prevalence' | 'To establish the presence or absence of trachoma in the Pacific Island region (population-based sample)' | 'Trachoma' | 'Australia' | 'WHO, Royal Victorian Eye and Ear Hospital' |
| 'Brian 2010' | 'Diabetic eye disease among adults in Fiji with self-reported diabetes.' | 'Population-based prevalence' | 'To characterize diabetic eye disease and its management among adults aged 40 years with self-reported diabetes in Fiji (population-based sample)' | 'Diabetic retinopathy' | 'NZ, Australia, Fiji' | 'NZAID, AusAID, FHFNZ' |
| 'Brian 2011' | 'Population-based study of self-reported ocular trauma in Fiji.' | 'Population-based prevalence' | 'To characterize causes, risk factors and outcomes for ocular trauma among adults aged 40 years in Fiji (population-based sample)' | 'Ocular trauma' | 'NZ, Australia, Fiji' | 'NZAID, AusAID, FHFNZ' |
| 'Brian 2011' | 'Cataract and its surgery in Fiji.' | 'Population-based prevalence' | 'To characterize cataract and its surgery among adults aged 40 years in Fiji' | 'Cataract' | 'NZ, Australia, Fiji' | 'NZAID, AusAID, FHFNZ' |
| 'Brian 2011' | 'Refractive error and presbyopia among adults in Fiji.' | 'Population-based prevalence' | 'To characterize refractive error, presbyopia and their correction among adults aged ≥ 40 years in Fiji, and contribute to a regional overview of these conditions (population-based sample)' | 'Refractive error' | 'New Zealand, Australia, Fiji' | 'NZAID, AusAID, FHFNZ' |
| 'Brian 2011' | 'Diabetic eye disease among adults in Fiji with previously undiagnosed diabetes.' | 'Population-based prevalence' | 'To determine prevalence and severity of diabetic retinopathy among adults >40 with previously undiagnosed diabetes in Fiji (population-based sample)' | 'Diabetic retinopathy' | 'New Zealand, Fiji, Australia' | 'NZAID, AusAID, FHFNZ' |
| 'Ramke 2012' | 'Prevalence and causes of blindness and low vision among adults in Fiji.' | 'Population-based prevalence' | 'To estimate the prevalence and causes of blindness and low vision among adults aged 40 years in Fiji' | 'VI±Causes' | 'NZ, Australia, Fiji' | 'NZAID, AusAID' |
| 'Macleod 2016' | 'Low Prevalence of Ocular Chlamydia trachomatis Infection and Active Trachoma in the Western Division of Fiji.' | 'Population-based prevalence' | 'To determine whether conjunctival infection with Chlamydia trachomatis (Ct) could be detected in one administrative division of Fiji' | 'Trachoma' | 'UK, Fiji, USA' | 'FHFAus, United Kingdom's Department of International Development (DFID), Global Trachoma Mapping Project, Sightsavers, Wellcome Trust, Govt of UK' |
| 'Butcher 2016' | 'Low Prevalence of Conjunctival Infection with Chlamydia trachomatis in a Treatment-Naive Trachoma-Endemic Region of the Solomon Islands.' | 'Population-based prevalence' | 'To determine the relationship between chlamydial infection and trachoma in region of Solomon Islands' | 'Trachoma' | 'UK, Solomon Islands, USA, Australia' | 'Wellcome Trust, UKDID, GTMP, Sightsavers, FHFAus' |
| 'Cocks 2016' | 'Community seroprevalence survey for yaws and trachoma in the Western Division of Fiji.' | 'Population-based prevalence' | 'To assess the seroprevalence of antibodies to T. pallidum and C. trachomatis in order to evaluate the need for further interventions for these NTDs in Fiji, using population-based cluster-randomised sampling' | 'Trachoma' | 'UK, Fiji' | 'Wellcome Trust, FHFAus' |
| 'Ko 2016' | 'Population-Based Trachoma Mapping in Six Evaluation Units of Papua New Guinea.' | 'Population-based prevalence' | 'To determine the prevalence of trachomatous inflammation – follicular (TF) in children aged 1–9 years, and trachomatous trichiasis (TT) in those aged ≥15 years, in suspected trachoma-endemic areas of Papua New Guinea (PNG)' | 'Trachoma' | 'PNG, UK, Solomon Islands, Australia, USA' | 'UKDID, Sightsavers, USAID, END in Asia, Pfizer' |
| 'Sokana 2016' | 'Mapping Trachoma in the Solomon Islands: Results of Three Baseline Population-Based Prevalence Surveys Conducted with the Global Trachoma Mapping Project.' | 'Population-based prevalence' | 'To complete the baseline trachoma map of the Solomon Islands by establishing prevalences of active trachoma and trichiasis in the provinces of Choiseul, Western, Rennell-Bellona, and Temotu' | 'Trachoma' | 'Solomon Islands, UK, USA' | 'GTMP, UKDID, Sightsavers, USAID, ENVISION, END in Asia, Pfizer, Wellcome Trust' |
| 'Macleod 2017' | 'Eyelash Epilation in the Absence of Trichiasis: Results of a Population-Based Prevalence Survey in the Western Division of Fiji.' | 'Population-based prevalence' | 'To understand significance of eyelash epilation and estimate the prevalence of it in the absence of trichiasis, and factors associated with this behaviour, in the Western Division of Fiji' | 'Trachoma' | 'UK, Fiji, Sweden' | 'Wellcome Trust, FHFAus' |
| 'Taleo 2017' | 'Integrated Mapping of Yaws and Trachoma in the Five Northern-Most Provinces of Vanuatu.' | 'Population-based prevalence' | 'To estimate the baseline prevalence of trachoma and yaws in the 5 northern-most provinces of Vanuatu (population-based sample)' | 'Trachoma' | 'Vanuatu, UK, Solomon Islands, South Africa, Switzerland, USA' | 'GTMP, UKDID, USAID, ENVISION, END in Asia, Pfizer, Wellcome Trust' |
| 'Cama 2017' | 'Prevalence of signs of trachoma, ocular Chlamydia trachomatis infection and antibodies to Pgp3 in residents of Kiritimati Island, Kiribati.' | 'Population-based prevalence' | 'To estimate prevalences of TF, TT, ocular Ct infection, and anti-Ct antibodies on Kiritimati Island, Kiribati, to assess local relationships between these parameters, and to help determine the need for interventions against trachoma on Kiribati islands other than Tarawa (using population-based sampling)' | 'Trachoma' | 'Fiji, Australia, Kiribati, UK, USA, Switzerland' | 'GTMP, Sightsavers, UKDID, Wellcome Trust, FHFAus, USAID, Pfizer, ENVISION, End in Asia' |
| 'Lee 2019' | 'Rapid assessment of avoidable blindness in Papua New Guinea: a nationwide survey.' | 'Population-based prevalence' | 'To estimate the prevalence and main causes of blindness and vision impairment in people aged 50 years and older in Papua New Guinea (PNG)' | 'VI±Causes' | 'Australia, PNG, UK, the Netherlands' | 'FHFAus' |
| 'Butcher 2020' | 'Ocular Chlamydia trachomatis infection, anti-Pgp3 antibodies and conjunctival scarring in Vanuatu and Tarawa, Kiribati before antibiotic treatment for trachoma.' | 'Population-based prevalence' | 'To demonstrate the utility of non-TF markers by contrasting the relationship between TF and ocular Ct infection in Vanuatu, where infection is suspected to be rare, to that in Kiribati, a neighbouring country where infection is suspected to be more common (population-based sample)' | 'Trachoma' | 'UK, Vanuatu, Kiribati, Solomon Islands, Portugal, Australia' | 'FHFAus, USAID, undação para a Ciência e Tecnologia Portugal, Wellcome Trust' |
| 'Lynch 2022' | 'A national survey integrating clinical, laboratory, and WASH data to determine the typology of trachoma in Nauru.' | 'Population-based prevalence' | 'To better understand the typology of trachoma and to determine whether there is a need for trachoma interventions (prevalence data also collected with population-based sampling)' | 'Trachoma' | 'Australia, Nauru, UK, USA, Switzerland' | 'FHFAus, Sightsavers, Core Trachoma Initiative, USAID, END in Asia' |
| 'Grosvenor 1988' | 'Myopia in Melanesian school children in Vanuatu' | 'School-based prevalence' | 'To describe refractive error in children in Vanuatu (school-based sample)' | 'Refractive error' | 'USA' | 'No Funding' |
| 'Garner 1985' | 'Prevalence of myopia in school children in Vanuatu.' | 'School-based prevalence' | 'To estimate prevalence of refractive errors in Melanesian children (school-based sample) and investigate genetic vs environmental contributors to myopia' | 'Refractive error' | 'NZ' | 'Govt of NZ' |
| 'Garner 1988' | 'Refraction and its components in Melanesian schoolchildren in Vanuatu.' | 'School-based prevalence' | 'To describe refraction in children (school-based sample)' | 'Refractive error' | 'NZ, USA' | 'Govt of NZ' |
| 'Garner 1990' | 'Ocular dimensions and refractive power in Malay and Melanesian children.' | 'School-based prevalence' | 'To compare ocular growth and the development of refractive error in the two groups' | 'Refractive error' | 'NZ, Malaysia' | 'South Pacific Medical Research Committee' |
| 'Lindquist 2011' | 'Screening for uncorrected refractive error in secondary school-age students in Fiji.' | 'School-based prevalence' | 'To estimate the rate of uncorrected refractive error and the associations with gender, age, ethnicity and place of residence' | 'Refractive error' | 'Australia, Fiji' | 'No Funding' |
| 'Hamm 2020' | 'Evaluation of vision screening of 5-15-year-old children in three Tongan schools: comparison of The Auckland Optotypes and Lea symbols.' | 'School-based prevalence' | 'To assess (1) the functional vision of children attending primary schools in Tonga and (2) how a new recognition acuity test (The Auckland Optotypes displayed on a tablet computer) compares to use of a standardised eye chart (school-based sample)' | 'VI±Causes' | 'NZ, Australia, Tonga, UK' | 'NZAID, Cure Kids, NZOVRF, Robert Leitl Trust, Ranchhold Foundation' |
| 'Owusu-Afriyie 2023' | 'Epidemiology of eye diseases: outcomes from a free provincial eye clinic in Papua New Guinea.' | 'Service-based various outcomes' | 'To ascertain the prevalence and pattern of eye problems in Madang Province, Papua New Guinea' | 'VI±Causes' | 'NZ, PNG' | 'FHFNZ' |
| 'Cronin-Lampe 2021' | 'Engagement with Diabetes Retinal Screening: An analysis of secondary data from the Pacific Eye Institute in Suva' | 'Service-based various outcomes' | 'To understand facilitators and barriers to diabetic retinopathy screening by retrospective review of patient data, including demographic information' | 'Diabetic retinopathy' | 'NZ, Fiji' | 'No Funding' |
| 'Davis 1982' | 'Retinopathy in malaria.' | 'Service-based various outcomes' | 'To determine the frequency of retinal hemorrhages in acuity malaria of al types and to examine various clinical and hematological indices in relation to their genesis' | 'Retinopathy (malaria)' | 'PNG' | 'No Funding' |
| 'Hamilton 1988' | 'Three years' experience of the East New Britain project for the disabled.' | 'Service-based various outcomes' | 'To register all disabled persons, determine types (and causes where possible) and needs, as well as assist disabled persons and educate the community.' | 'VI±Causes' | 'PNG, UK' | 'No Funding' |
| 'Pryor 1994' | 'Vitamin A deficiency and otitis media in Chuuk State, Micronesia.' | 'Service-based various outcomes' | 'To report the prevalence of OM and Vitamin A Deficiency (VAD) in Chuuk State, Micronesia, and their public health implications, to explore the potential relationship between OM and VAD, and to demonstrate that simple data gathering and PHC training/screening technique are transferable to PHC workers in the field. ' | 'Vitamin A deficiency' | 'USA' | 'Micronesia Otitis Media Training Project, Maternal Child Health Improvement Project, Govt of USA' |
| 'Durand 1997' | 'Diabetes in the indigenous population of the Commonwealth of the Northern Mariana Islands.' | 'Service-based various outcomes' | 'To determine the prevalence of diabetes in the Indigenous population of Commonwealth of the North Mariana Islands, from medical records' | 'Diabetic retinopathy' | 'USA, Northern Mariana Islands' | 'No Funding' |
| 'Duke 2017' | 'Prevalence of non-communicable disease in rural Vanuatu.' | 'Service-based various outcomes' | 'To assess prevalence rates for diabetes, hypertension, obesity and eye disease for adults in Vanuatu, South Pacific.' | 'VI±Causes' | 'Australia, Vanuatu' | 'Roche Diagnostics Australia, Aspen Pharmacare' |
| 'Bern 1993' | 'Acute hemorrhagic conjunctivitis due to enterovirus 70 in American Samoa: Serum-neutralizing antibodies and sex-specific protection' | 'Service-based various outcomes' | 'To describe outbreak and look for evidence of protection from clinical disease from previous outbreaks.' | 'Conjunctivitis' | 'USA' | 'No Funding' |
| 'Steele 2015' | 'A unique retinal epitheliopathy is associated with amyotrophic lateral sclerosis/Parkinsonism-Dementia complex of Guam' | 'Service-based various outcomes' | 'To examine whether a linear retinal pigment epitheliopathy is associated with the amyotrophic lateral sclerosis/parkinsonism-dementia complex of Guam.' | 'Retinopathy (ALS-PDC)' | 'Guam, USA, Canada, UK' | 'CurePSP' |
| 'Macleod 2020' | 'Trachoma, anti-Pgp3 serology and ocular Chlamydia trachomatis infection in Papua New Guinea' | 'Service-based various outcomes' | 'To supplement routine trachoma mapping with CT infection data to help guide local policy development and expand the evidence base on trachoma in Melanesia (population-based sample)' | 'Trachoma' | 'UK, PNG, USA, Singapore, Australia' | 'UKDID, USAID, END in Asia, GTMP, Sightsavers' |
| 'Ree 1980' | 'Ocular leprosy in Papua New Guinea.' | 'Service-based various outcomes' | 'To describe ocular lesions in leprosy patients by type, duration and activity of the disease ' | 'Ocular leprosy' | 'PNG' | 'No Funding' |
| 'Hornabrook 1980' | 'Congenital nystagmus among the Red-skins of the Highlands of Papua New Guinea.' | 'Service-based various outcomes' | 'To understand nystagmus in what appears to be a unique form of albinism in a rural area' | 'Nystagmus' | 'PNG, UK' | 'No Funding' |
| 'Dethlefs 1981' | 'Anterior uveitis in Papua New Guinea.' | 'Service-based various outcomes' | 'To describe case studies of acute anterior uveitis (iritis), presenting at clinic' | 'Anterior uveitis' | 'PNG' | 'No Funding' |
| 'Dethlefs 1981' | 'Prevalence of ocular manifestations of leprosy in Port Moresby, Papua New Guinea.' | 'Service-based various outcomes' | 'To document the prevalence and type of ocular complications seen in leprosy patients in Port Moresby.' | 'Ocular leprosy' | 'PNG' | 'No Funding' |
| 'Anonymous 1982' | 'Acute hemorrhagic conjunctivitis--American Samoa.' | 'Service-based various outcomes' | 'To report an outbreak of severe conjunctivitis on Tutuila, the main island of American Samoa. ' | 'Conjunctivitis' | 'USA' | 'CDC (USA)' |
| 'Dethlefs 1982' | 'Glaucoma in Port Moresby Papua New Guinea.' | 'Service-based various outcomes' | 'To describe patients with glaucoma who presented over a two year period at Port Moresby General Hospital, Papua New Guinea. ' | 'Glaucoma' | 'Australia' | 'No Funding' |
| 'Parsons 1982' | 'An ocular survey of community school children in Madang Province.' | 'Service-based various outcomes' | 'To 1) initiate a school ocular health service, 2) screen young children for ocular deficits including refractive errors, colour perception deficiency and organic or functional ocular lesions, 3) assess the incidence and severity of trachoma in this group, and treat the florid cases to prevent future complications, 4) train medical auxiliary workers and nursing staff in methods of screening for ocular pathology (school-based sampling)' | 'VI±Causes' | 'PNG' | 'No Funding' |
| 'Onorato 1985' | 'Acute hemorrhagic conjunctivitis caused by enterovirus type 70: an epidemic in American Samoa.' | 'Service-based various outcomes' | 'To establish incidence rates of acute haemorrhagic conjunctivitis (AHC) caused by Enterovirus Type 70 in American Samoa, and to evaluate possible risk factors for AHC acquisition ' | 'Conjunctivitis' | 'USA, American Samoa' | 'No Funding' |
| 'Kawabe 1985' | 'Visual acuity of the Gidra in lowland Papua New Guinea.' | 'Service-based various outcomes' | 'To measure VA and anterior segment health in rural (hunter and gatherer) community in PNG' | 'Refractive error' | 'Japan' | 'Govt of Japan' |
| 'Parsons 1986' | 'Ocular trauma in Papua New Guinea.' | 'Service-based various outcomes' | 'To characterise ocular trauma seen in Papua New Guinea over a 5-year period.' | 'Ocular trauma' | 'PNG' | 'No Funding' |
| 'Andrist 1986' | 'Visual characteristics of natives in Fiji, South Pacific.' | 'Service-based various outcomes' | 'To aid in the planning of future projects by summarizing data on refractive and other ocular anomalies encountered in a population of 800 Fijians examined during the summer of 1983 (outreach sample)' | 'VI±Causes' | 'USA' | 'No Funding' |
| 'Egbert 1986' | 'Ophthalmic disease in Western Samoa.' | 'Service-based various outcomes' | 'To describe eye diseases seen during a set period of time, mostly clinical' | 'VI±Causes' | 'USA' | 'No Funding' |
| 'Parsons 1987' | 'Blindness in Papua New Guinea.' | 'Service-based various outcomes' | 'To understand characteristics of blind people in PNG seen over a 5-year period (1980-1984)' | 'VI±Causes' | 'PNG' | 'No Funding' |
| 'Adams 1987' | 'Toxoplasma antibodies and retinochoroiditis in the Marshall Islands and their association with exposure to radioactive fallout.' | 'Service-based various outcomes' | 'To determine if radiation exposure may have produced long term immunosuppression which might place the exposed population at greater risk for clinical illness due to toxoplasmosis. A second goal of the investigation was to evaluate the prevalence of Toxoplasma seropositivity and the prevalence and incidence of suspected Toxoplasma retinochoroiditis in a larger population of Marshallese which included unexposed individuals.' | 'Ocular toxoplasmosis' | 'USA' | 'Govt of USA' |
| 'Tefuarani 1988' | 'Childhood malignant tumours in Papua New Guinea.' | 'Service-based various outcomes' | 'To determine the incidence and patterns of malignancies in children in Papua New Guinea' | 'Tumors/growths' | 'PNG' | 'No Funding' |
| 'Sawyer 1989' | 'An epidemic of acute hemorrhagic conjunctivitis in American Samoa caused by coxsackievirus A24 variant.' | 'Service-based various outcomes' | 'To describe the 1986 epidemic of acute hemorrhagic conjunctivitis' | 'Conjunctivitis' | 'USA, American Samoa' | 'No Funding' |
| 'Cox 1989' | 'A retinopathy on Guam with high prevalence in Lytico-Bodig.' | 'Service-based various outcomes' | 'To understand the relationship between Lytico- Bodig and a unique retinopathy on Guam.' | 'Retinopathy (ALS-PDC)' | 'Canada, Guam, USA' | 'Govt of Canada' |
| 'Maisel 1989' | 'Large optic disks in the Marshallese population.' | 'Service-based various outcomes' | 'To compare the fundus photographs of a group of patients noted to have high cup/disk ratios and that of a selected group of control patients in order to prove that the optic disks that appeared on ophthalmoscopic examination to have high cup/disk ratios were actually larger than those optic disks judged clinically normal on ophthalmoscopy.' | 'Large optic disks' | 'USA' | 'Govt of USA' |
| 'Parsons 1991' | 'A decade of ophthalmic statistics in Papua New Guinea.' | 'Service-based various outcomes' | 'To describe opthalmological practice in a clinic in PNG both quantitatively and qualitatively' | 'VI±Causes' | 'PNG' | 'No Funding' |
| 'Danks 1992' | 'A clinical and cytological study of vitamin A deficiency in Kiribati.' | 'Service-based various outcomes' | 'To assess the Vitamin A status of children (birth to six years) by clinical and cytological means.' | 'Vitamin A deficiency' | 'Australia' | 'ASPECT' |
| 'Campbell 1993' | 'Pathologic findings in the retinal pigment epitheliopathy associated with the amyotrophic lateral sclerosis/parkinsonism-dementia complex of Guam.' | 'Service-based various outcomes' | 'To pathologically investigate the retinal pigment epitheliopathy associated with the amyotrophic lateral sclerosis/parkinsonism-dementia complex of people in Guam' | 'Retinopathy (ALS-PDC)' | 'USA, Guam' | 'NIH' |
| 'Seaton 1997' | 'Visual loss in immunocompetent patients with Cryptococcus neoformans var. gattii meningitis.' | 'Service-based various outcomes' | 'To review ophthalmic findings in 82 immunocompetent patients and attempt to identify those features of the disease that predict an unfavourable visual outcome.' | 'Meningitis' | 'PNG, UK' | 'No Funding' |
| 'Hudson 1997' | 'Giant millipede 'burns' and the eye.' | 'Service-based various outcomes' | 'To determine the frequency and severity of eye conditions due to ‘burns’ from these millipedes, and to review management.' | 'Millipede burns' | 'PNG' | 'No Funding' |
| 'Verma 1997' | 'Profile of ocular trauma in Papua New Guinea.' | 'Service-based various outcomes' | 'To determine the pattern and rates of ocular and adnexal injuries so as to determine the size and extent of the problem.' | 'Ocular trauma' | 'PNG' | 'No Funding' |
| 'Brooks 1999' | 'Diabetic retinopathy and nephropathy in Fiji: comparison with data from an Australian diabetes centre.' | 'Service-based various outcomes' | 'To assess the magnitude of problems posed by diabetic retinopathy in Fiji and compare the findings with those from an Australian diabetes centre.' | 'Diabetic retinopathy' | 'Australia, Fiji' | 'AusAID' |
| 'Verma 1999' | 'Orbital malignancy in Papua New Guinea: a 21 year review.' | 'Service-based various outcomes' | 'To analyse the pattern of orbital malignancies seen in PNG over a 21 year period and to see how it differed from that reported in other parts of the world' | 'Tumors/growths' | 'PNG' | 'No Funding' |
| 'Mancel 1999' | 'Clinical aspects of ocular leptospirosis in New Caledonia (South Pacific).' | 'Service-based various outcomes' | 'To investigate the role of pathogenic leptospires as an aetiological agent of ocular leptospirosis.' | 'Ocular leprosy' | 'New Caledonia' | 'No Funding' |
| 'Yamamoto 2004' | 'Causes of blindness and the results of cataract surgical intervention in the Micronesian islands: a retrospective study.' | 'Service-based various outcomes' | 'To report the causes of blindness and the results of cataract surgical intervention in the Micronesian islands' | 'VI±Causes' | 'Japan' | 'Govt of Japan, Yomiuri Light and Humanity Association, Govt of Micronesia' |
| 'Baker 2005' | 'Sight-seeing in the Solomon Islands.' | 'Service-based various outcomes' | 'To describe the eye care needs and services in the Solomon Islands from a personal perspective, gained during the eye surgery support visit to the Solomon Islands under Pacific Island Project (PIP)' | 'VI±Causes' | 'Australia' | 'AusAID' |
| 'Balachandra 2005' | 'Coral stone landscape and pterygia; is there an association?.' | 'Service-based various outcomes' | 'To find an association between pterygia, diabetes mellotus and the coral stones used in RMI. ' | 'Tumors/growths' | 'Marshall Islands' | 'Outer Island Health Care' |
| 'Smith 2007' | 'The prevalence and severity of diabetic retinopathy, associated risk factors and vision loss in patients registered with type 2 diabetes in Luganville, Vanuatu.' | 'Service-based various outcomes' | 'To determine the prevalence and severity of diabetic retinopathy in patients with type 2 diabetes in Luganville, the second largest town in Vanuatu. Additionally, to investigate risk factors for retinopathy and the effect of retinopathy on visual acuity (VA) within this group.' | 'Diabetic retinopathy' | 'UK, Solomon Islands' | 'Royal College of Ophth, Commonwealth Foundation' |
| 'Tay 2007' | 'Nanophthalmos in a Melanesian population.' | 'Service-based various outcomes' | 'To characterize the ophthalmic features and causes of visual loss in a cohort of Melanesians living in New Caledonia with nanophthalmos.' | 'Micro or an-ophthalmia' | 'Australia, New Caledonia, UK' | 'Western Sydney Genetics Prgm, Children's Hospital (Westmead)' |
| 'Ramke 2007' | 'Eye disease and care at hospital clinics in Cook Islands, Fiji, Samoa and Tonga.' | 'Service-based various outcomes' | 'To obtain eye disease and care data to assist with service planning in Cook Islands, Fiji, Samoa and Tonga (through staff interview and audit)' | 'VI±Causes' | 'Australia, NZ' | 'FHFNZ, NZAID' |
| 'Olsson 2008' | 'Conjunctivitis outbreak among divers.' | 'Service-based various outcomes' | 'To document the case of an outbreak involving diving equipment - likely due to a communal container to store diving masks as a likely vector to spread infectious conjunctivitis.' | 'Conjunctivitis' | 'USA' | 'No Funding' |
| 'Cama 2010' | 'Childhood visual impairment in Fiji.' | 'Service-based various outcomes' | 'To establish the prevalence and causes of low vision and blindness in children aged 0 to 15 years in Fiji using existing data and new surveys' | 'VI±Causes' | 'Australia, Fiji' | 'Govt of Australia, Lions Club' |
| 'Qoqonokana 2010' | 'Diabetic retinopathy in a hospital eye clinic population in Honiara, Solomon Islands.' | 'Service-based various outcomes' | 'To determine the presence, severity and context of diabetic retinopathy among diabetic adults using hospital eye clinic services in Honiara, Solomon Islands' | 'Diabetic retinopathy' | 'Fiji, NZ, Australia' | 'FHFNZ, NZAID, AusAID' |
| 'Yomai 2010' | 'Microphthalmia and anophthalmia in Chuuk State, Federated States of Micronesia.' | 'Service-based various outcomes' | 'To explore potential putative genetic and environmental causes previously associated with rare congenital defects of eye development (microphthalmia and anophthalmia) in Chuuk, Federated States of Micronesia, where alarmingly high rates have been observed. The study will hopefully shed light on the prevention of these conditions.' | 'Micro or an-ophthalmia' | 'Micronesia' | 'No Funding' |
| 'Brian 2011' | 'Serum retinol and xerophthalmia among a prison population in Papua New Guinea.' | 'Service-based various outcomes' | 'To estimate the prevalence of vitamin A deficiency (VAD) and one of its clinical manifestations, xerophthalmia, and examine the predictive value of nyctalopia and ocular signs for serum retinol concentrations among a prison population in Papua New Guinea.' | 'Vitamin A deficiency' | 'NZ, Australia, PNG' | 'NZAID, KOHA-PICD, FHFNZ, U of Otago (NZ)' |
| 'Barnes 2011' | 'Survey on Visual Impairment and Refractive Errors on Ta'u Island, American Samoa.' | 'Service-based various outcomes' | 'To assess the prevalence of presenting visual impairment and refractive errors on the isolated island of Ta’u, American Samoa.' | 'VI±Causes' | 'USA' | 'Hawaii and Pacific Basin Grant' |
| 'WinTin 2014' | 'The prevalence of diabetes complications and associated risk factors in Pacific Islands countries.' | 'Service-based various outcomes' | 'To determine the prevalence of diabetes complications and associated risk factors among people with type 2 diabetes attending diabetes clinics in three Pacific Island countries, Nauru, Solomon Islands and Vanuatu.' | 'Diabetic retinopathy' | 'Australia, Solomon Islands, Nauru, Vanuatu' | 'World Diabetes Foundation' |
| 'Damato 2014' | 'Sight-threatening diabetic retinopathy at presentation to screening services in Fiji.' | 'Service-based various outcomes' | 'To report the spectrum of retinopathy at first presentation to photoscreening services, to determine the proportion of patients that present with sight-threatening diabetic retinopathy (STDR), and to raise awareness of the burden of diabetic eye disease in Fiji.' | 'Diabetic retinopathy' | 'NZ, Fiji' | 'FHFAus' |
| 'Baker 2014' | 'Profile of ocular trauma in the Solomon Islands.' | 'Service-based various outcomes' | 'To characterize the causes of ocular trauma and determine the risk factors for infection and vision loss following ocular trauma in the Solomon Islands' | 'Ocular trauma' | 'Australia, Fiji, Solomon Islands' | 'Allergan Australia' |
| 'Lees 2015' | 'Eye health outreach services in the Pacific Islands region: an updated profile.' | 'Service-based various outcomes' | 'To analyse and describe the records of eye health outreach clinics from a single provider in seven Pacific Islands.' | 'VI±Causes' | 'NZ' | 'No Funding' |
| 'Jeganathan 2017' | 'Screening for Diabetic Eye Disease among Samoan Adults: A Pilot Study.' | 'Service-based various outcomes' | 'To determine the prevalence of diabetic retinopathy in Samoa by piloting a retinal photography screening programme.' | 'Diabetic retinopathy' | 'USA, Australia, Samoa' | 'No Funding' |
| 'Bhikoo 2017' | 'Demographic features and visual outcomes of patients presenting to diabetic photo-screening and treated for sight threatening retinopathy in Fiji.' | 'Service-based various outcomes' | 'To describe the demographic features and visual outcomes of patients presenting to photo-screening services, and treated for sight threatening retinopathy (STR) in a low resource setting, Fiji' | 'Diabetic retinopathy' | 'NZ, Fiji' | 'No Funding' |
| 'Gushchin 2017' | 'Simbu Ptosis: An Outreach Approach to Myogenic Ptosis in Eastern Highlands of Papua New Guinea-Experience and Results From a High-Volume Oculoplastic Surgical Camp.' | 'Service-based various outcomes' | 'To present the results of a high-volume oculoplastic surgical outreach in a remote region of Simbu Province in the Eastern Highlands of Papua New Guinea. The authors describe the clinical features and evaluation and treatment of a novel ptosis syndrome found in this area (outreach sample)' | 'Ptosis' | 'USA, Nepal' | 'Monica Mercer' |
| 'Milner 2017' | 'Neurodevelopmental outcomes for high-risk neonates in a low-resource setting.' | 'Service-based various outcomes' | 'To assess early childhood neurodevelopmental and health outcomes for a cohort of high-risk neonates, defined on the basis of NICU admission, relative to control healthy, term neonates in Fiji, specifically: 1. prevalence of moderate to severe NDI at approximately 2 years of age 2. prevalence of stunting, wasting, anaemia, other health morbidity, immunisation rates and health service usage' | 'Neurodevelopmental visual impairment' | 'Australia, UK, Fiji' | 'AusAID, Cure Kids Fiji' |
| 'Owusu-Afriyie 2022' | 'Prevalence of Risk Factors of Retinal Diseases among Patients in Madang Province, Papua New Guinea.' | 'Service-based various outcomes' | 'To explore the prevalence of risk factors of retinal diseases among patients seeking services from Madang Provincial Hospital Eye Clinic in Papua New Guinea.' | 'VI±Causes' | 'PNG' | 'No Funding' |
| 'Owusu-Afriyie 2022' | 'Knowledge and Awareness of Diabetes and Diabetic Retinopathy among Patients Seeking Eye Care Services in Madang Province, Papua New Guinea.' | 'Service-based various outcomes' | 'To assess the knowledge and awareness of diabetes and diabetic retinopathy among ophthalmic patients in Madang' | 'Diabetic retinopathy' | 'PNG, NZ' | 'No Funding' |
| 'Owusu-Afriyie 2022' | 'Prevalence and Distribution of Refractive Errors among Ophthalmic Patients in Madang Province, Papua New Guinea.' | 'Service-based various outcomes' | 'To assess the prevalence and distribution of refractive errors in Madang Province, Papua New Guinea' | 'Refractive error' | 'NZ, PNG' | 'No Funding' |

# Publications primarily about Eye Health Services

| Study ID | Title | Focus | Aim | Eye condition | Authors affiliation | Funding | P | S | D | T | R |
| --- | --- | --- | --- | --- | --- | --- | --- | --- | --- | --- | --- |
| 'Butcher 2018' | 'Clinical signs of trachoma are prevalent among Solomon Islanders who have no persistent markers of prior infection with Chlamydia trachomatis' | 'Basic science' | 'To determine whether there is evidence of ongoing transmission and pathology from ocular Chlamydia trachomatis (Ct) infection.' | 'Trachoma' | 'UK, Solomon Islands, USA' | 'Wellcome Trust, FHFAus, Intermediate Clinical Fellowship, Inst Strategic Support Fund, USAID' | '✗' | '✗' | '✗' | '✗' | '✗' |
| 'Grosvenor 1988' | 'High axial length/corneal radius ratio as a risk factor in the development of myopia.' | 'Basic science' | 'To present evidence to suggest that the relationship between axial lenght of the eye and corneal radius is useful to predict refractive state' | 'Refractive error' | 'USA' | 'No Funding' | '✓' | '?' | '✗' | '✗' | '✗' |
| 'Winick 1999' | 'Homozygosity mapping of the Achromatopsia locus in the Pingelapese.' | 'Basic science' | 'To obtain insights into the genetic basis of achromatopsia, as well as into the genetic history of Pingelapese kindreds with achromatopsia.' | 'Achromatopsia' | 'USA' | 'Rockefeller University, NIH, Irma T Hirschel Award' | '✗' | '✗' | '?' | '✗' | '✗' |
| 'Sundin 2000' | 'Genetic basis of total colourblindness among the Pingelapese islanders.' | 'Basic science' | 'To describe the genetic basis of total colour blindness in Pingelap' | 'Achromatopsia' | 'USA' | 'Research center (Johns Hopkins), Foundation for Retinal Research, Grousbeck Family Foundation, The Louise Sloan Trust, Wilmer Intramural Grant, Research to Prevent Blindness' | '✗' | '✗' | '?' | '✗' | '✗' |
| 'Butcher 2017' | 'Active Trachoma Cases in the Solomon Islands Have Varied Polymicrobial Community Structures but Do Not Associate with Individual Non-Chlamydial Pathogens of the Eye.' | 'Basic science' | 'To investigate whether active trachoma was associated with a common non-chlamydial infection or with a dominant polymicrobial community dysbiosis in the Solomon Islands' | 'Trachoma' | 'UK, Solomon Islands, Australia' | 'UKDID, GTMP, Sightsavers, FHFAus, Wellcome Trust, Govt of Solomon Islands' | '✗' | '✗' | '✗' | '?' | '✗' |
| 'Vasileva 2018' | 'Conjunctival transcriptome profiling of Solomon Islanders with active trachoma in the absence of Chlamydia trachomatis infection.' | 'Basic science' | 'To characterise patterns of gene expression at the conjunctivae of children in these provinces with and without clinical signs of trachomatous inflammation-follicular (TF) and C. trachomatis infection.' | 'Trachoma' | 'UK, Solomon Islands' | 'UKDID, Global Trachoma Mapping Project, Sightsavers, FHFAus, Wellcome Trust' | '?' | '✗' | '?' | '✗' | '✗' |
| 'Johnson 2023' | 'Viruses Associated with Acute Conjunctivitis in Vanuatu.' | 'Basic science' | 'To investigate the viruses that were associated with acute conjunctivitis.' | 'Conjunctivitis' | 'Vanuatu, Switzerland, USA' | 'NIH, Research to Prevent Blindness, Peierls Foundation' | '?' | '✗' | '?' | '✗' | '✗' |
| 'Albakri 2023' | 'Novel CRB1 pathogenic variant in Chuuk families with Leber congenital amaurosis.' | 'Basic science' | 'To determine the cause of Leber congenital amaurosis (LCA) in Chuuk state, Federated States of Micronesia (FSM). We describe the discovery of a novel frameshift pathogenic variant in CRB1, as the cause of LCA in Chuuk, Micronesia, a geographically isolated population.' | 'Leber congenital amaurosis' | 'USA, Saudi Arabia, Thailand, Micronesia' | 'The Foerderer Fund, Robison Harley Endowed Chair, NIH, Albert Einstein Society' | '?' | '✗' | '✗' | '✗' | '✗' |
| 'Garap 2021' | 'Eye health in Papua New Guinea.' | 'Commentary' | 'To highlights the progress made in eye health in PNG and the role of the National Prevention of Blindness Committee' | 'Not condition specific' | 'PNG, UK' | 'The Brien Holden Foundation, CBM, Govt of Australia, UKDID, FHFAus, IAPB, Laila Foundation, Lions Club, International Foundation, USAID' | '✗' | '✗' | '✗' | '✗' | '✗' |
| 'Vlaardingerbroek 1992' | 'Integrated primary schooling of blind children in Papua New Guinea.' | 'Commentary' | 'To describe the preparatory training and integrated primary schooling carried out in Goroka, Eastern Highlands for blind children in PNG' | 'Not condition specific' | 'PNG' | 'No Funding' | '✗' | '✗' | '✗' | '✗' | '✓' |
| 'Galbraith 1994' | 'ASPECT in the Pacific: walking the aid tightrope. Australian South Pacific Eye Consultant Teams.' | 'Commentary' | 'To discuss the key issues in the provision of aid to developing countries to develop the provision of eye health services' | 'Not condition specific' | 'Australia' | 'No Funding' | '✗' | '✗' | '✗' | '✗' | '✗' |
| 'Szetu 2005' | 'Vanuatu national eye care programme.' | 'Commentary' | 'To describe the origins, achievements and challenges of the Vanuatu National Eye Care Programme. ' | 'Not condition specific' | 'Vanuatu' | 'No Funding' | '✗' | '✗' | '✗' | '✗' | '✗' |
| 'duToit 2009' | 'Education of eye health professionals to meet the needs of the Pacific.' | 'Commentary' | 'To assess the use of education best practice in ophthalmic programmes delivered at the Pacific Eye Institute to tackle eye health workforce shortages and improve workforce effectiveness.' | 'Not condition specific' | 'NZ' | 'No Funding' | '✗' | '✗' | '✗' | '✗' | '✗' |
| 'Pahau 2009' | 'Free cataract surgery may undermine local services in developing countries.' | 'Commentary' | 'To comment on the effect of visiting teams provoding free of charges cataract surgery' | 'Cataract' | 'PNG, NZ' | 'No Funding' | '✗' | '✗' | '✗' | '?' | '✗' |
| 'Brule 2017' | 'An in-country model of workforce support for trained mid-level eye care workers in Papua New Guinea and Pacific Islands.' | 'Commentary' | 'To describe the in-country model of workforce support for trained mid-level eye care workers in Papua New Guinea and the Pacific Islands' | 'Not condition specific' | 'Canada, NZ, USA' | 'No Funding' | '✗' | '✗' | '✗' | '✗' | '✗' |
| 'Machin 2017' | 'Celebrating the Diverse Roles of Pacific Island Ophthalmic Nurses and Technicians.' | 'Commentary' | 'To highlight and celebrate the diverse roles of Pacific Island ophthalmic nurses and technicians' | 'Not condition specific' | 'NZ, Australia' | 'No Funding' | '✗' | '✗' | '✗' | '✗' | '✗' |
| 'Baral 2020' | 'Eye nurse training in the Pacific islands.' | 'Commentary' | 'To describe the ophthalmic nursing program in Fiji and PNG' | 'Not condition specific' | 'Nepal' | 'No Funding' | '✗' | '✗' | '✗' | '✗' | '✗' |
| 'Burnett 2016' | 'Experiences of vision impairment in Papua New Guinea: implications for blindness prevention programs' | 'Perspective (patient)' | 'To better understand the beliefs, perceptions and emotional responses to vision impairment in PNG' | 'Not condition specific' | 'Australia, PNG' | 'Avoidable Blindness Initiative, Govt of Australia' | '?' | '✗' | '✗' | '✗' | '✗' |
| 'Szmedra 2007' | 'Small island states in crisis: the economic impact of lifestyle diseases in the South Pacific.' | 'Perspective (patient)' | 'To monetize the annual economic loss suffered in Nauru and Fiji as a result of NCD' | 'Not condition specific' | 'USA, Fiji' | 'U of the South Pacific' | '✗' | '✗' | '✗' | '✗' | '✗' |
| ''Ofanoa 2021' | 'A qualitative study of patient perspectives of diabetes and diabetic retinopathy services in Vanuatu.' | 'Perspective (patient)' | 'To explore the perceptions of diabetic patients in Vanuatu on these issues, to help inform the design of health promotion materials and community activities to empower people to self-manage and shape diabetic services that are integrated and people-centred.' | 'Diabetic retinopathy' | 'NZ, Vanuatu' | 'FHFNZ' | '✓' | '✗' | '✗' | '✗' | '✗' |
| 'Kuruvatti 2002' | 'Life is sweet in Fiji: The availability of diabetes healthcare and health education, and diabetes awareness amongst diabetic patients in Fiji' | 'Perspective (patient)' | 'To assess the knowledge of diabetes possessed by patients who attended the hospital Diabetic Clinic in Lautoka' | 'Diabetic retinopathy' | 'UK' | 'No Funding' | '✓' | '✗' | '✗' | '✗' | '✗' |
| ''Ofanoa 2020' | 'Patient Perspectives of Diabetes and Diabetic Retinopathy Services in Kiribati: A Qualitative Study' | 'Perspective (patient)' | 'To provide an understanding of patients with diabetes’ knowledge and perceptions of diabetes, health promotion, and associated health care services to help inform the design of effective health promotion materials, community engagement activities, and effective patient consultations.' | 'Diabetic retinopathy' | 'New Zealand, Kiribati' | 'FHFNZ' | '✓' | '✗' | '✗' | '✗' | '✗' |
| 'Anonymous 2005' | 'Diabetes-related preventive-care practices--Guam, 2001-2003.' | 'Perspective (patient)' | 'To determine the prevalence of preventive-care practices among persons with diabetes in Guam (wider study was a population-based sample, random-digit–dialed telephone survey of noninstitutionalized civilian adults aged >18 years)' | 'Diabetic retinopathy' | 'Guam, USA' | 'CDC (USA)' | '✓' | '✗' | '✗' | '✗' | '✗' |
| 'duToit 2006' | 'Awareness and use of eye care services in Fiji.' | 'Perspective (patient)' | 'To determine the awareness, use, and barriers to use of eye services in Fiji’s Central Province (population-based sample)' | 'Not condition specific' | 'Australia' | 'Govt of Australia, Intl Centre for Eye Care Ed' | '?' | '✗' | '?' | '?' | '✗' |
| 'duToit 2008' | 'Spectacles in Fiji: need, acquisition, use and willingness to pay.' | 'Perspective (patient)' | 'To assess the need for spectacles and the status of the spectacle supply system.' | 'Refractive error' | 'NZ, Australia' | 'Govt of Australia, Vision SRC, The Optometric Vision Research Foundation' | '✗' | '✗' | '✗' | '✓' | '✗' |
| 'Brian 2012' | 'Eye care in Fiji: a population-based study of use and barriers.' | 'Perspective (patient)' | 'To determine the use of medical services for eye problems in Fiji, and barriers to seeking that care (population-based sample)' | 'Not condition specific' | 'NZ, Australia' | 'NZAID, AusAID, FHFNZ' | '?' | '✗' | '✗' | '✗' | '✗' |
| 'Burnett 2015' | 'Perceptions of Eye Health and Eye Health Services among Adults Attending Outreach Eye Care Clinics in Papua New Guinea.' | 'Perspective (patient)' | 'To determine how people attending outreach eye care clinics in Papua New Guinea (PNG) perceive eye health and eye health services' | 'Not condition specific' | 'Australia, PNG' | 'Govt of Australia' | '?' | '✗' | '✗' | '✗' | '✗' |
| 'Caceres 2023' | 'Diabetic Retinopathy Screening Using a Portable Retinal Camera in Vanuatu.' | 'Pilot/validation of tool' | 'To test the feasibility of using a portable, handheld retinal camera for the screening of referral-warranted diabetic retinopathy (proof-of-concept study)' | 'Diabetic retinopathy' | 'USA, Vanuatu' | 'National Eye Institute, Alcon Research Institute, Research to Prevent Blindness, Helmut F Stern Professorship, University of Michigan' | '✗' | '✓' | '✗' | '✗' | '✗' |
| 'Adachi 1999' | 'Usefulness of hand-held automatic refractometer in volunteer medical activities in Vanuatu' | 'Pilot/validation of tool' | 'To understand the usefulness of hand-held automatic refractometer by medical volunteers to carry out refraction services in Vanuatu.' | 'Refractive error' | 'Japan' | 'No Funding' | '✗' | '✓' | '✗' | '✗' | '✗' |
| 'Maqsood 2004' | 'Vitamin A deficiency and inflammatory markers among preschool children in the Republic of the Marshall Islands' | 'Pilot/validation of tool' | 'To determine whether the exclusion of individuals with elevated acute phase proteins is associated with sampling bias and to characterize inflammation in children with night blindness.' | 'Vitamin A deficiency' | 'USA, Marshall Islands' | 'Pacific Health Research Inst, UNICEF, Fergusson Foundation, Hawaii Community Foundation, NIH' | '?' | '✗' | '✗' | '✗' | '✗' |
| 'Cappa 2018' | 'The development and testing of a module on child functioning for identifying children with disabilities on surveys. III: Field testing' | 'Pilot/validation of tool' | 'To document the testing of the module and summarize its results, including a description of prevalence levels across countries using different cut-offs, and comparisons with prevalence levels obtained using the TQSI and the WG-SS' | 'Not condition specific' | 'USA, Serbia, Mexico' | 'No Funding' | '✗' | '✓' | '✗' | '✗' | '?' |
| 'Sprunt 2019' | 'The UNICEF/Washington group child functioning module-accuracy, inter-rater reliability and cut-off level for disability disaggregation of fiji's education management information system' | 'Pilot/validation of tool' | 'To determine the validity (sensitivity and specificity) of different cut-off levels of the UNICEF/ Washington Group Child Functioning Module (CFM) for predicting the presence of disabilities in primary school aged Fijian children compared to standard clinical assessments of impairment, and also to determine the inter-rater reliability between teacher and parent CFM responses' | 'Not condition specific' | 'Australia' | 'Govt of Australia' | '✗' | '?' | '✓' | '✗' | '✓' |
| 'Mactaggart 2021' | 'Interrogating and reflecting on disability prevalence data collected using the washington group tools: Results from population-based surveys in cameroon, guatemala, india, maldives, nepal, turkey and vanuatu' | 'Pilot/validation of tool' | 'To compare disability prevalence estimates by Washington Group module, threshold, application and domain and assess whether alternative combinations of questions may be valuable.' | 'Not condition specific' | 'UK, Turkey, Guatemala, Nepal, Cameroon, USA, India' | 'No Funding' | '✗' | '✗' | '✗' | '✗' | '✓' |
| 'Garner 1985' | 'Use of an automatic refraction device in a Third World country.' | 'Pilot/validation of tool' | 'To examine the feasibility of providing spectacles form the measurements obtained by an automatic refraction instrument in the hands of a naïve operator.' | 'Refractive error' | 'NZ' | 'Nikon, French Airlines, Govt of NZ, NZOA, Optical Holdings Limited' | '✗' | '✓' | '✗' | '✗' | '✗' |
| 'Delaplain 1993' | 'Tripler pioneers telemedicine across the Pacific.' | 'Pilot/validation of tool' | 'To investigate the efficiency of telemedicine.' | 'Not condition specific' | 'USA, Marshall Islands' | 'No Funding' | '✗' | '✗' | '✓' | '✗' | '✗' |
| 'Nakhate 2008' | 'Using basic technology to screen for diabetic retinopathy in Fiji.' | 'Pilot/validation of tool' | 'To explore the use of basic retinal screening for detecting DR' | 'Diabetic retinopathy' | 'Fiji, USA' | 'No Funding' | '✗' | '✓' | '✗' | '✗' | '✗' |
| 'O'Connor 2010' | 'Validation of a quality of life questionnaire in the Pacific Island.' | 'Pilot/validation of tool' | 'To adapt an existing validated quality of life instrument, the Impact of Vision Impairment (IVI) questionnaire for Pacific Island countries.' | 'Not condition specific' | 'Australia' | 'Govt of Australia' | '✗' | '✗' | '✗' | '✗' | '✓' |
| 'Williams 2012' | 'Measuring vision-specific quality of life among adults in Fiji.' | 'Pilot/validation of tool' | 'To evaluate an 18-item vision-specific quality-of-life questionnaire designed for use with adults in Fiji' | 'Not condition specific' | 'NZ, Australia' | 'NZAID, AusAID, FHFNZ' | '✗' | '✗' | '?' | '✗' | '✓' |
| 'Paudel 2015' | 'Papua New Guinea vision-specific quality of life questionnaire: a new patient-reported outcome instrument to assess the impact of impaired vision.' | 'Pilot/validation of tool' | 'To develop and validate a new vision-specific quality of life (VS QoL) instrument and to assess the impact of vision impairment and eye disease on the quality of life of adults in Papua New Guinea (PNG)' | 'Not condition specific' | 'Australia, PNG' | 'AusAID' | '?' | '✗' | '?' | '✗' | '✓' |
| 'Sprunt 2019' | 'Validating the UNICEF/Washington Group Child Functioning Module for Fijian schools to identify seeing, hearing and walking difficulties.' | 'Pilot/validation of tool' | 'To investigate the seeing, hearing and walking questions of the UNICEF/Washington Group Child Functioning Module and the inter-rater reliability between teachers and parents as proxy respondents' | 'Not condition specific' | 'Australia' | 'Govt of Australia' | '✗' | '✗' | '?' | '✗' | '✓' |
| 'LaMonica 2019' | 'Nonmydriatic fundus photography in a high-risk population of Samoans with diabetes: The Soifua Manuia eye screening program.' | 'Pilot/validation of tool' | 'To field-test the PanOptic iExaminer System to screen for retinal complications stemming from diabetes in a remote, resource-constrained setting' | 'Diabetic retinopathy' | 'USA, Samoa' | 'No Funding' | '✗' | '✓' | '✗' | '✗' | '✗' |
| 'LaMonica 2021' | 'Remote Screening for Optic Nerve Cupping Using Smartphone-based Nonmydriatic Fundus Photography.' | 'Pilot/validation of tool' | 'To investigate factors that impact inter-rater agreement of glaucoma suspect optic disc status using a low-cost, handheld nonmydriatic fundus camera' | 'Glaucoma' | 'USA, Samoa' | 'NIH, Downs Intl Health Fellowship, Yale School of Public Health, Harry Lee Family Chair' | '✗' | '✓' | '?' | '✗' | '✗' |
| 'Lehmann 1999' | 'High rates of Chlamydia trachomatis infections in young Papua New Guinean infants' | 'Service evaluation / improvement' | 'To determine the importance of Chlamydia trachomatis in the etiology of severe infection in young Papua New Guinean infants.' | 'Conjunctivitis' | 'Philippines, PNG, Switzerland, Australia, UK' | 'WHO (Acute Respiratory Infection)' | '?' | '✗' | '?' | '✗' | '✗' |
| 'Falconer 2010' | 'Counting the cost of type 2 diabetes in Vanuatu' | 'Service evaluation / improvement' | 'To determine the health system costs, cost to people with diabetes and their carers, and impact on quality of life associated with type 2 diabetes in Vanuatu' | 'Diabetic retinopathy' | 'Australia' | 'Govt of Vanuatu, Aus and NZ Society of Neph, Intl Centre for Eyecare Ed' | '?' | '✗' | '✗' | '✗' | '✗' |
| 'Brian 1999' | 'Ophthalmic interventions in the developing world: insights for successful outcomes.' | 'Service evaluation / improvement' | 'To describe the Pacific Island Project (PIP) on Vanuatu (the teams' goals are (i) to treat correctable blindness and (ii) to teach local people ophthalmic skills so that we can ultimately become redundant.' | 'Not condition specific' | 'Australia' | 'AusAID' | '✗' | '?' | '✗' | '✓' | '✗' |
| 'Kool 2015' | 'DR services in Fiji: attitudes, barriers and screening practices.' | 'Service evaluation / improvement' | 'To describe the attitudes and perceptions of primary health care doctors in Fiji regarding the importance of eye care in diabetes mellitus (DM) management, to explore current eye care practice, and to investigate awareness and use of relevant clinical practice guidelines.' | 'Diabetic retinopathy' | 'NZ' | 'No Funding' | '?' | '✓' | '?' | '✗' | '✗' |
| 'WinTin 2016' | 'Comparing metabolic control and complications in type 2 diabetes in two Pacific Islands at baseline and following diabetes care intervention' | 'Service evaluation / improvement' | 'To compare metabolic control and complications in people with type 2 diabetes in Nauru and the Solomon Islands before and after a project intervention' | 'Diabetic retinopathy' | 'Australia, Solomon Islands, Nauru' | 'MoH Nauru, MoH Solomon Islands, International Centre for Eyecare Ed, Aus and NZ Society of Neph' | '✓' | '✗' | '?' | '✗' | '✗' |
| 'Leppaniemi 1990' | 'Surgery in Tuvalu: a 10-year review.' | 'Service evaluation / improvement' | 'To estimate the number and types of surgeries in a 10 Year period, from medical records (clinical sample)' | 'Cataract' | 'Tuvalu' | 'No Funding' | '✗' | '✗' | '✗' | '✓' | '✗' |
| 'Seaton 1997' | 'The effect of corticosteroids on visual loss in Cryptococcus neoformans var. gattii meningitis.' | 'Service evaluation / improvement' | 'To compare treatment regimes for type of meingitis on visual loss (retrospctive)' | 'Meningitis' | 'PNG, UK' | 'No Funding' | '✗' | '✗' | '✗' | '?' | '✗' |
| 'Verma 1998' | 'Intraoperative use of mitomycin C in the treatment of recurrent pterygium.' | 'Service evaluation / improvement' | 'To compare the results of a single intraoperative application of 0.02% mitomycin C solution with a similar group of 65 patients in which the drug was not used.' | 'Pterygium' | 'PNG' | 'No Funding' | '?' | '✗' | '✗' | '✓' | '✗' |
| 'Ramke 2006' | 'An assessment of recycled spectacles donated to a developing country.' | 'Service evaluation / improvement' | 'To determine the suitability of donated recycled spectacles for the Pacific nation of Tuvalu' | 'Refractive error' | 'Australia, NZ' | 'NZOVRF, Govt of Australia' | '✗' | '✗' | '✗' | '✓' | '✗' |
| 'Pahau 2006' | 'Monitoring cataract surgery outcomes in Papua New Guinea.' | 'Service evaluation / improvement' | 'To monitor and analyze cataract surgery outcomes in PNG' | 'Cataract' | 'PNG, NZ' | 'NZAID, FHFNZ' | '✗' | '✗' | '?' | '?' | '✗' |
| 'Yap 2006' | 'Assessment of clinical notes in Papua New Guinea.' | 'Service evaluation / improvement' | 'To examine the comprehensiveness and utility of records in the Port Moresby General Hospital Eye Clinic by retrospectively assessing outpatient notes of 100 consecutive new patients, and compared them with an idealized record template.' | 'Not condition specific' | 'PNG, NZ' | 'NZAID, FHFNZ' | '✗' | '✗' | '✗' | '✗' | '✗' |
| 'Williams 2008' | 'Evaluation of the first 5 years of a national eye health programme in Vanuatu.' | 'Service evaluation / improvement' | 'To evaluate against its objectives the achievements of the first 5 years of a national eye health programme in Vanuatu.' | 'Not condition specific' | 'NZ, Fiji' | 'No Funding' | '✗' | '✗' | '✗' | '✗' | '✗' |
| 'Ramke 2008' | 'Public sector refraction and spectacle dispensing in low-resource countries of the Western Pacific.' | 'Service evaluation / improvement' | 'To conduct an appraisal of public sector arrangements for the correction of refractive error in eight Pacific Island countries' | 'Refractive error' | 'NZ' | 'No Funding' | '?' | '?' | '?' | '?' | '✗' |
| 'Hobday 2011' | 'Eye health promotion in Western Pacific island countries.' | 'Service evaluation / improvement' | 'To assess the status of eye health promotion activities in WPIC' | 'Not condition specific' | 'NZ' | 'No Funding' | '✗' | '✗' | '✗' | '✗' | '✗' |
| 'Williams 2012' | 'Using health rights to improve programme design: a Papua New Guinea case study.' | 'Service evaluation / improvement' | 'To use a rights-based tool to assess the design of activities proposed for Papua New Guinea by a consortium of Australian non-government organisations.' | 'Not condition specific' | 'NZ' | 'No Funding' | '✗' | '✗' | '✗' | '✗' | '✗' |
| 'Pikacha 2015' | 'Cataract Surgery Outcomes From a Resident Training in a Low-Resource Setting in the Pacific.' | 'Service evaluation / improvement' | 'To assess the overall quality of the cataract surgeries performed by a single resident during training in a core surgical competency in a low-resource environment' | 'Cataract' | 'Solomon Islands, NZ' | 'No Funding' | '✗' | '✗' | '✗' | '?' | '✗' |
| 'Marella 2017' | 'The situation of low vision services in Papua New Guinea: an exploratory study.' | 'Service evaluation / improvement' | 'To investigate the current situation of low vision services and barriers to low vision service in PNG' | 'Not condition specific' | 'Australia, PNG' | 'Govt of Australia, Brien Holden Vision Institute' | '✗' | '✗' | '✗' | '✗' | '✓' |
| 'Bhikoo 2017' | 'Short-term outcomes of small incision cataract surgery provided by a regional population in the Pacific.' | 'Service evaluation / improvement' | 'To describe the patient demographics, visual and surgical outcomes of eyes undergoing SICS at a regional ophthalmic teaching institute in Fiji.' | 'Cataract' | 'NZ, Fiji' | 'No Funding' | '✗' | '✗' | '✗' | '✓' | '✗' |
| 'Bhikoo 2018' | 'Small incision cataract surgery provided by a regional population in the Pacific: a 12-month follow-up.' | 'Service evaluation / improvement' | 'To share follow up data on visual and surgical outcomes after cataract surgery (@12 months)' | 'Cataract' | 'NZ, Fiji' | 'No Funding' | '✗' | '✗' | '✗' | '✓' | '✗' |
| 'Romani 2018' | 'Feasibility and safety of mass drug coadministration with azithromycin and ivermectin for the control of neglected tropical diseases: a single-arm intervention trial.' | 'Service evaluation / improvement' | 'To investigate the feasibility and safety of mass drug administration of two integrated therapies with azithromycin and ivermectin in a single-arm intervention trial' | 'Trachoma' | 'Australia, UK, Solomon Islands, Switzerland' | 'Intl Trachoma Initiative, Murdoch Research Inst, Mackinnon Trust, Wellcome Trust, Govt of Solomon Islands' | '✗' | '✗' | '✗' | '✓' | '✗' |
| 'Meyer 2019' | 'Improved Refractive Outcomes of Small-Incision Extracapsular Cataract Surgery after Implementation of a Biometry Training Course.' | 'Service evaluation / improvement' | 'To determine whether a biometry training course could improve refractive outcomes of patients undergoing manual small-incision extracapsular cataract surgery (SICS).' | 'Cataract' | 'NZ, Fiji' | 'FHFNZ' | '✗' | '✗' | '✗' | '✓' | '✗' |
| 'Vince 2017' | 'Forty years of postgraduate medical training at the University of Papua New Guinea.' | 'Workforce development' | 'To analyse the output of the various postgraduate programs, and to put this in the context of the National Department of Health (NDoH) Hospital standards' | 'Not condition specific' | 'PNG' | 'No Funding' | '✗' | '✗' | '✗' | '✗' | '✗' |
| 'Farmer 2000' | 'Developing eye care in papua new Guinea.' | 'Workforce development' | 'To describe current eye care workforce, and future directions ' | 'Not condition specific' | 'Australia' | 'No Funding' | '✗' | '✗' | '✗' | '✗' | '✗' |
| 'duToit 2010' | 'The development of competency-based education for mid-level eye care professionals: a process to foster an appropriate, widely accepted and socially accountable initiative.' | 'Workforce development' | 'To develop competencies for mid-level eye care professionals to meet quality outcomes.' | 'Not condition specific' | 'NZ' | 'No Funding' | '?' | '?' | '?' | '?' | '?' |
| 'duToit 2011' | 'Facilitating the quality of care in a specialist Pacific ophthalmic nursing workforce.' | 'Workforce development' | 'To assess influences on the quality of care provided by specialist mid-level ophthalmic personnel in WPICT and devise strategies to train, retain and maintain performance of these personnel' | 'Not condition specific' | 'PNG, NZ, Kenya' | 'No Funding' | '✗' | '✗' | '?' | '?' | '✗' |
| 'Tousignant 2011' | 'Adapting the World Federation for Medical Education standards for use in a self-audit of an eye care training programme.' | 'Workforce development' | 'To describe the evaluation of a newly instated eye nurse training programme' | 'Not condition specific' | 'NZ' | 'FHFNZ' | '✗' | '✗' | '✗' | '✗' | '✗' |
| 'Tousignant 2020' | 'State of the eye health system in the Pacific: is medical technology available and used by mid-level eye care workers?.' | 'Workforce development' | 'To describe the availability, use and comfort with ophthalmic equipment and medications by mid-level eye care workers in Papua New Guinea and Pacific Island countries and territories as indicators of the state of eye care in the Pacific.' | 'Not condition specific' | 'Canada, USA, Fiji, NZ' | 'No Funding' | '✗' | '✗' | '✗' | '✗' | '✗' |
| 'Ram 2022' | 'Impact of diabetic retinopathy awareness training on community health workers' knowledge and referral practices in Fiji: a qualitative study.' | 'Workforce development' | 'To explore the impact of training on CHWs’ knowledge of DR and their referral practices related to DR screening in Fiji 2 years post-training' | 'Diabetic retinopathy' | 'NZ, Fiji' | 'FHFNZ' | '?' | '?' | '✗' | '✗' | '✗' |
| 'Ram 2022' | 'Increasing and sustaining diabetic retinopathy screening in Fiji by leveraging community health workers (CHWs) services: A qualitative study.' | 'Workforce development' | 'To describe the development and implementation of DR awareness training for community health workers (CHWs) and their subsequent engagement to raise awareness and scale-up DR screening for communities throughout Fiji.' | 'Diabetic retinopathy' | 'NZ, UK, Fiji' | 'FHFNZ' | '✗' | '✓' | '✗' | '✗' | '✗' |

Acronyms and abbreviations used for reporting funders

| Funder acronyms and abbreviations | |
| --- | --- |
| 'FHFNZ' | 'Fred Hollows Foundation New Zealand' |
| 'FHFAus' | 'Fred Hollows Foundation Australia (used then unspecified country)' |
| 'NZAID' | 'New Zealand Agency for International Development/Aid and Development' |
| 'AusAID' | 'Australian Agency for International Development' |
| 'USAID' | 'United States Agency for International Development' |
| 'UKDID' | 'United Kingdom’s Department for International Development' |
| 'NIH' | 'National Institute of Health (United States)' |
| 'GTMP' | 'Global Trachoma Mapping Project' |
| 'ASPECT' | 'Australian South Pacific Eye Consultant Team' |
| 'CDC' | 'Centre for Disease control' |
| 'NZOVRF' | 'New Zealand Optometric Vision Research Foundation' |
| 'WHO' | 'World Health Organization' |
| 'Aus and NZ Society of Neph' | 'Australia and New Zealand Society for Nephrology' |
| 'Peoples of the South Pacific' | 'Foundation for the Peoples of the South Pacific' |
| 'UNICEF' | 'United Nations International Children's Emergency Fund' |
| 'CurePSP' | 'Also reported as: Foundation for PSP/CBD (PSP: Progressive Supranuclear Palsy, CBD: Cortico-Basal Degeneration' |
| **General abbreviations used in reporting funders** | |
| 'Govt' | 'Government' |
| 'Intl' | 'International' |
| 'Inst' | 'Institute' |
| 'Prgm' | 'Programme' |

# Annex 3: Search Strategy

Ovid MEDLINE(R) ALL <1946 to May 24, 2023>

----------------------------------------------------------------------

1 Pacific Islands/ (4170)

2 exp Polynesia/ (11382)

3 Hawaii/ (8450)

4 2 not 3 (2932)

5 exp Melanesia/ (7129)

6 exp Micronesia/ (2144)

7 New Zealand/ (44050)

8 6 not 7 (2128)

9 (Micronesia or Melanesia or Palau or Guam or Nauru or Vanuatu or Fiji or Tuvalu or Tonga or Samoa$ or Tokelau or Niue or Kiribati).tw. (7463)

10 (Papua adj1 New adj1 Guinea).tw. (4917)

11 (New adj1 Caledonia).tw. (1695)

12 (Wallis adj2 Futuna).tw. (47)

13 (French adj2 Polynesia).tw. (1146)

14 Pitcairn.tw. (43)

15 ((Cook or Marshall or Pacific or Solomon) adj2 (Island$ or territor$ or area$)).tw. (9718)

16 (Northern adj1 Mariana adj1 Island$).tw. (155)

17 1 or 4 or 5 or 8 or 9 or 10 or 11 or 12 or 13 or 14 or 15 or 16 (28246)

18 exp Eye Diseases/ (634421)

19 exp Cataract Extraction/ (36991)

20 Lens Implantation, Intraocular/ (13174)

21 Lenses, Intraocular/ (16370)

22 ((intraocular or intra ocular) adj3 lens$).tw. (20179)

23 (IOL or IOLs).tw. (12683)

24 cataract$.tw. (63592)

25 (myopia or hyperop$ or hypermetrop$ or anisometrop$ or ammetrop$ or astigmati$ or presbyop$).tw. (36098)

26 (refractive adj1 error$).tw. (11465)

27 Eyeglasses/ (8092)

28 (spectacle or spectacles).tw. (7546)

29 (eyeglasses or eye glasses).tw. (1017)

30 exp Visual Acuity/ (92359)

31 (visual adj1 acuit$).tw. (79688)

32 ((macul$ or retina$) adj2 degener$).tw. (34887)

33 (AMD or ARMD or CNV or maculopath$).tw. (30733)

34 (glaucoma$ or IOP or OHT).tw. (79115)

35 (ocular adj1 hypertension).tw. (5873)

36 ((diabet$ or proliferat$) adj3 retinopath$).tw. (31476)

37 (diabet$ adj3 (eye$ or vision or visual$ or sight$)).tw. (5719)

38 (retinopath$ adj3 (eye$ or vision or visual$ or sight$)).tw. (2585)

39 (dilated adj2 fundus).tw. (877)

40 ((eye$ or vision or retina$ or ophthalm$ or retinopath$) adj2 exam$).tw. (28876)

41 ((eye$ or vision or retina$ or retinopath$ or ophthalm$) adj2 assess$).tw. (6765)

42 ((eye$ or vision or retina$ or ophthalm$ or retinopath$) adj2 test$).tw. (8127)

43 Visually Impaired Persons/ (2744)

44 ((low$ or impair$ or partial$ or loss$ or limit$) adj3 (vision or visual$ or sight$)).tw. (71345)

45 exp Vision Tests/ (117229)

46 exp Vision, Ocular/ (29833)

47 (trachoma$ or tracoma$ or trichiasis).tw. (19437)

48 onchocerciasis.tw. (3913)

49 (retinopath$ adj2 prematur$).tw. (7956)

50 (amblyop$ or strabismus).tw. (19709)

51 exp Diagnostic Techniques, Ophthalmological/ (191247)

52 exp Ophthalmologic Surgical Procedures/ (123147)

53 Ophthalmology/ (24971)

54 Optometry/ (5733)

55 Orthoptics/ (1895)

56 (ophthalmology or optometry or orthoptics).tw. (28568)

57 Ophthalmologists/ (907)

58 Optometrists/ (222)

59 (ophthalmologist$ or optometrist$ or optician$ or orthopist$ or refractionists).tw. (19087)

60 (ophthalmic adj3 (surgeon$ or physician$ or nurse$ or technician$ or officer$ or assistant$ or staff$ or worker$)).tw. (1049)

61 (eye$ adj3 (surgeon$ or physician$ or nurse$ or technician$ or officer$ or assistant$ or staff$ or worker$)).tw. (1510)

62 (eye$ adj2 (care or health or service$)).tw. (7181)

63 ((centre$ or center$ or camp$ or clinic$ or hospital$) adj2 (vision or eye$ or refraction or refractive)).tw. (14225)

64 or/18-63 (889621)

65 17 and 64 (566)

66 (mouse or mice or rat or rats).ti. (1434988)

67 (sexual$ or syphili$ or vagina$ or gonorrhoeae or STI or genital$).tw. (476005)

68 (Fiji adj3 (ImageJ$ or OCT$ or software or plugin$)).tw. (361)

69 or/66-68 (1893169)

70 65 not 69 (476)

71 limit 70 to yr="1980 -Current" (428)

Embase

----------------------------------------------------------------------

1. cook islands/ or "federated states of micronesia"/ or fiji/ or french polynesia/ or guam/ or kiribati/ or marshall islands/ or melanesia/ or nauru/ or new caledonia/ or niue/ or northern mariana islands/ or palau/ or papua new guinea/ or pitcairn/ or polynesia/ or samoan islands/ or solomon islands/ or tokelau/ or tonga/ or tuvalu/ or vanuatu/ or "wallis and futuna"/

2. (Micronesia or Melanesia or Palau or Guam or Nauru or Vanuatu or Fiji or Tuvalu or Tonga or Samoa$ or Tokelau or Niue or Kiribati).tw.

3. (Papua adj1 New adj1 Guinea).tw.

4. (New adj1 Caledonia).tw.

5. (Wallis adj2 Futuna).tw.

6. (French adj2 Polynesia).tw.

7. Pitcairn.tw.

8. ((Cook or Marshall or Pacific or Solomon) adj2 (Island$ or territor$ or area$)).tw.

9. (Northern adj1 Mariana adj1 Island$).tw.

10. or/1-9

11. exp eye disease/

12. lens implantation/

13. ((intraocular or intra ocular) adj3 lens$).tw.

14. (IOL or IOLs).tw.

15. cataract$.tw.

16. (myopia or hyperop$ or hypermetrop$ or anisometrop$ or ammetrop$ or astigmati$ or presbyop$).tw.

17. (refractive adj1 error$).tw.

18. spectacles/

19. (spectacle or spectacles).tw.

20. (eyeglasses or eye glasses).tw.

21. exp visual acuity/

22. (visual adj1 acuit$).tw.

23. ((macul$ or retina$) adj2 degener$).tw.

24. (AMD or ARMD or CNV or maculopath$).tw.

25. (glaucoma$ or IOP or OHT).tw.

26. (ocular adj1 hypertension).tw.

27. ((diabet$ or proliferat$) adj3 retinopath$).tw.

28. (diabet$ adj3 (eye$ or vision or visual$ or sight$)).tw.

29. (retinopath$ adj3 (eye$ or vision or visual$ or sight$)).tw.

30. (dilated adj2 fundus).tw.

31. ((eye$ or vision or retina$ or ophthalm$ or retinopath$) adj2 exam$).tw.

32. ((eye$ or vision or retina$ or retinopath$ or ophthalm$) adj2 assess$).tw.

33. ((eye$ or vision or retina$ or ophthalm$ or retinopath$) adj2 test$).tw.

34. visually impaired person/

35. ((low$ or impair$ or partial$ or loss$ or limit$) adj3 (vision or visual$ or sight$)).tw.

36. exp visual system examination/

37. exp visual system function/

38. eye care/

39. (trachoma$ or tracoma$ or trichiasis).tw.

40. onchocerciasis.tw.

41. (retinopath$ adj2 prematur$).tw.

42. (amblyop$ or strabismus).tw.

43. exp eye surgery/

44. ophthalmology/

45. optometry/

46. orthoptics/

47. (ophthalmology or optometry or orthoptics).tw.

48. ophthalmologist/

49. optometrist/

50. eye care professional/

51. (ophthalmologist$ or optometrist$ or optician$ or orthopist$ or refractionist*).tw.

52. (ophthalmic adj3 (surgeon$ or physician$ or nurse$ or technician$ or officer$ or assistant$ or staff$ or worker$)).tw.

53. (eye$ adj3 (surgeon$ or physician$ or nurse$ or technician$ or officer$ or assistant$ or staff$ or worker$)).tw.

54. (eye$ adj2 (care or health or service$)).tw.

55. ((centre$ or center$ or camp$ or clinic$ or hospital$) adj2 (vision or eye$ or refraction or refractive)).tw.

56. or/11-55

57. 10 and 56

58. (mouse or mice or rat or rats).ti.

59. (sexual$ or syphili$ or vagina$ or gonorrhoeae or STI or genital$).tw.

60. (Fiji adj3 (ImageJ$ or OCT$ or software or plugin$)).tw.

61. (vari$ adj3 (copy or genetic$)).ti.

62. or/58-61

63. 57 not 62

Global Health

----------------------------------------------------------------------

1. exp pacific islands/

2. (Micronesia or Melanesia or Palau or Guam or Nauru or Vanuatu or Fiji or Tuvalu or Tonga or Samoa$ or Tokelau or Niue or Kiribati).tw.

3. (Papua adj1 New adj1 Guinea).tw.

4. (New adj1 Caledonia).tw.

5. (Wallis adj2 Futuna).tw.

6. (French adj2 Polynesia).tw.

7. Pitcairn.tw.

8. ((Cook or Marshall or Pacific or Solomon) adj2 (Island$ or territor$ or area$)).tw.

9. (Northern adj1 Mariana adj1 Island$).tw.

10. or/1-9

11. exp eye diseases/

12. eyes/

13. exp vision disorders/

14. vision/

15. ((intraocular or intra ocular) adj3 lens$).tw.

16. (IOL or IOLs).tw.

17. cataract$.tw.

18. (myopia or hyperop$ or hypermetrop$ or anisometrop$ or ammetrop$ or astigmati$ or presbyop$).tw.

19. (refractive adj1 error$).tw.

20. (spectacle or spectacles).tw.

21. (eyeglasses or eye glasses).tw.

22. (visual adj1 acuit$).tw.

23. ((macul$ or retina$) adj2 degener$).tw.

24. (AMD or ARMD or CNV or maculopath$).tw.

25. (glaucoma$ or IOP or OHT).tw.

26. (ocular adj1 hypertension).tw.

27. ((diabet$ or proliferat$) adj3 retinopath$).tw.

28. (diabet$ adj3 (eye$ or vision or visual$ or sight$)).tw.

29. (retinopath$ adj3 (eye$ or vision or visual$ or sight$)).tw.

30. (dilated adj2 fundus).tw.

31. ((eye$ or vision or retina$ or ophthalm$ or retinopath$) adj2 exam$).tw.

32. ((eye$ or vision or retina$ or retinopath$ or ophthalm$) adj2 assess$).tw.

33. ((eye$ or vision or retina$ or ophthalm$ or retinopath$) adj2 test$).tw.

34. people with visual impairment/

35. ((low$ or impair$ or partial$ or loss$ or limit$) adj3 (vision or visual$ or sight$)).tw.

36. (trachoma$ or tracoma$ or trichiasis).tw.

37. onchocerciasis.tw.

38. (retinopath$ adj2 prematur$).tw.

39. (amblyop$ or strabismus).tw.

40. (ophthalmology or optometry or orthoptics).tw.

41. (ophthalmologist$ or optometrist$ or optician$ or orthopist$ or refractionist*).tw.

42. (ophthalmic adj3 (surgeon$ or physician$ or nurse$ or technician$ or officer$ or assistant$ or staff$ or worker$)).tw.

43. (eye$ adj3 (surgeon$ or physician$ or nurse$ or technician$ or officer$ or assistant$ or staff$ or worker$)).tw.

44. (eye$ adj2 (care or health or service$)).tw.

45. ((centre$ or center$ or camp$ or clinic$ or hospital$) adj2 (vision or eye$ or refraction or refractive)).tw.

46. or/11-45

47. 10 and 46

48. (mouse or mice or rat or rats).ti.

49. (sexual$ or syphili$ or vagina$ or gonorrhoeae or STI or genital$).tw.

50. (Fiji adj3 (ImageJ$ or OCT$ or software or plugin$)).tw.

51. (vari$ adj3 (copy or genetic$)).ti.

52. or/48-51

53. 47 not 52

CENTRAL on the Cochrane Library

----------------------------------------------------------------------

#1 MeSH descriptor: [Pacific Islands] this term only

#2 MeSH descriptor: [Polynesia] explode all trees

#3 MeSH descriptor: [Hawaii] this term only

#4 #2 not #3

#5 MeSH descriptor: [Melanesia] explode all trees

#6 MeSH descriptor: [Micronesia] explode all trees

#7 MeSH descriptor: [New Zealand] this term only

#8 #6 not #7

#9 #1 or #4 or #5 or #8

#10 Micronesia or Melanesia or Palau or Guam or Nauru or Vanuatu or Fiji or Tuvalu or Tonga or Samoa$ or Tokelau or Niue or Kiribati

#11 Papua NEAR/1 New NEAR/1 Guinea

#12 New NEAR/1 Caledonia

#13 Wallis NEAR/2 Futuna

#14 French NEAR/2 Polynesia

#15 Pitcairn

#16 (Cook or Marshall or Pacific or Solomon) NEAR/2 (Island* or territor* or area*)

#17 Northern NEAR/1 Mariana NEAR/1 Island*

#18 #10 or #11 or #12 or #13 or #14 or #15 or #16 or #17

#19 #9 or #18

#20 MeSH descriptor: [Eye Diseases] explode all trees

#21 MeSH descriptor: [Cataract Extraction] explode all trees

#22 MeSH descriptor: [Lens Implantation, Intraocular] this term only

#23 MeSH descriptor: [Lenses, Intraocular] this term only

#24 (intraocular or intra ocular) NEAR/3 lens*

#25 IOL or IOLs

#26 cataract*

#27 myopia or hyperop* or hypermetrop* or anisometrop* or ammetrop* or astigmati* or presbyop*

#28 refractive NEAR/1 error*

#29 MeSH descriptor: [Eyeglasses] this term only

#30 spectacle or spectacles

#31 eyeglasses or eye glasses

#32 MeSH descriptor: [Visual Acuity] explode all trees

#33 visual NEAR/1 acuit*

#34 (macul* or retina*) NEAR/2 degener*

#35 AMD or ARMD or CNV or maculopath*

#36 glaucoma* or IOP or OHT

#37 ocular NEAR/1 hypertension

#38 (diabet* or proliferat*) NEAR/3 retinopath*

#39 diabet* NEAR/3 (eye* or vision or visual* or sight*)

#40 retinopath* NEAR/3 (eye* or vision or visual* or sight*)

#41 dilated NEAR/2 fundus

#42 (eye* or vision or retina* or ophthalm* or retinopath*) NEAR/2 exam*

#43 (eye* or vision or retina* or retinopath* or ophthalm*) NEAR/2 assess*

#44 (eye* or vision or retina* or ophthalm* or retinopath*) NEAR/2 test*

#45 MeSH descriptor: [Visually Impaired Persons] this term only

#46 (low* or impair* or partial* or loss* or limit*) NEAR/3 (vision or visual* or sight*)

#47 MeSH descriptor: [Vision Tests] explode all trees

#48 MeSH descriptor: [Vision, Ocular] explode all trees

#49 trachoma* or tracoma* or trichiasis

#50 onchocerciasis

#51 retinopath* NEAR/2 prematur*

#52 amblyop* or strabismus

#53 MeSH descriptor: [Diagnostic Techniques, Ophthalmological] explode all trees

#54 MeSH descriptor: [Ophthalmologic Surgical Procedures] explode all trees

#55 MeSH descriptor: [Ophthalmology] this term only

#56 MeSH descriptor: [Optometry] this term only

#57 MeSH descriptor: [Orthoptics] this term only

#58 ophthalmology or optometry or orthoptics

#59 MeSH descriptor: [Ophthalmologists] this term only

#60 MeSH descriptor: [Optometrists] this term only

#61 ophthalmologist* or optometrist* or optician* or orthopist* or refractionist*

#62 ophthalmic NEAR/3 (surgeon* or physician* or nurse* or technician* or officer* or assistant* or staff* or worker*)

#63 eye* NEAR/3 (surgeon* or physician* or nurse* or technician* or officer* or assistant* or staff* or worker*)

#64 eye* NEAR/2 (care or health or service*)

#65 (centre* or center* or camp* or clinic* or hospital*) NEAR/2 (vision or eye* or refraction or refractive)

#66 #20 or #21 or #22 or #23 or #24 or #25 or #26 or #27 or #28 or #29 or #30 or #31 or #32 or #33 or #34 or #35 or #36 or #37 or #38 or #39 or #40 or #41 or #42 or #43 or #44 or #45 or #46 or #47 or #48 or #49 or #50 or #51 or #52 or #53 or #54 or #55 or #56 or #57 or #58 or #59 or #60 or #61 or #62 or #63 or #64 or #65

#67 #19 and #66

#68 sexual* or syphili* or vagina* or gonorrhoeae or STI or genital*

#69 Fiji adj3 (ImageJ* or OCT* or software or plugin*)

#70 #68 or #69

#71 #67 not #70
