# Supplementary material for: Research about eye health and eye health services in Pacific Island Countries and Territories: a scoping review
Source: Lancet Reg Health West Pac. 2024 Jul 27;50:101152. doi: 10.1016/j.lanwpc.2024.101152 (PMC11332796; doi:10.1016/j.lanwpc.2024.101152)
Supplement: ANNEX 4_Hamm et al Overview by Country [file mmc2.pdf]

Annex 4: Overview of eye health research by Pacific Island Country and Territory

Table of Contents

Publications conducted across more than one country ii
What was the research about? ii
Who is conducting the research? ii
Papua New Guinea iv
What was the research about? iv
Who is conducting the research? iv
Fiji v
What was the research about? v
Who is conducting the research? v
Vanuatu vi
What was the research about? vi
Who is conducting the research? vi
Solomon Islands vii
What was the research about? vii
Who is conducting the research? vii
Federated states of Micronesia viii
What was the research about? viii
Who is conducting the research? viii
Marshall Islands ix
What was the research about? ix
Who is conducting the research? ix
Nauru x
What was the research about? x
Who is conducting the research? x
Samoa xi
What was the research about? xi
Who is conducting the research? xi
American Samoa xii
What was the research about? xii
Who is conducting the research? xii
Guam xiii
What was the research about? xiii
Who is conducting the research? xiii
Kiribati xiv
What was the research about? xiv
Who is conducting the research? xiv
New Caledonia xv
Tonga xv
Tuvalu xv
Cook Islands xv
Niue xv
Northern Mariana Islands xv

Publications conducted across more than one country

What was the research about?

Twenty publications (11%) included more than one Pacific Island Country, area, or territory. For these multi-country publications, four reported population-wide prevalence of specific conditions. The two most recent of these (both published in 2020) estimated the prevalence of trachoma (one in Vanuatu and Kiribati, and another across Solomon Islands and Vanuatu). The other two population-based prevalence studies included an investigation of the prevalence of trachoma in 2009 (including Fiji, Kiribati, Nauru, Solomon Islands, and Vanuatu) and a 1995 publication to understand the magnitude of vitamin A deficiency across five countries (Cook Islands, Kiribati, Solomon Island and Vanuatu). There was also a notable publication conducted in Nauru and the Solomon Islands that compared complications of diabetes before and after an intervention.

Just under half of the multi-country publications did not to target a specific condition (n=9/20, 45%). These publications were looking at the bigger picture of service provision in the region, many specifically targeting workforce training development. Even commentaries about more than one Pacific Island Country, area, or territory tended to focus on the reflections on the eye care workforce. Multi-country research appears to be a newer trend, with most publications published since 2005.

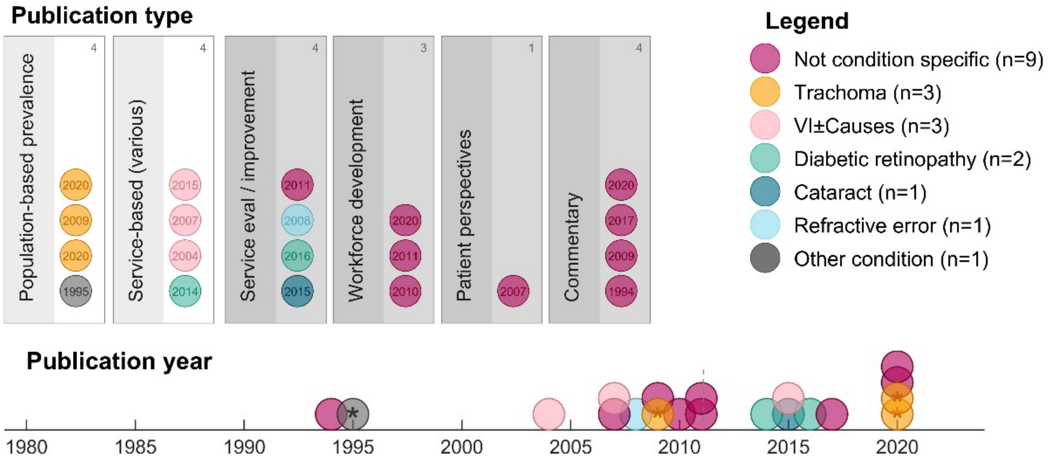

Figure 1. Publication type (upper panel) and timeline (lower panel). Each publication is represented by a dot, including study ID. Colour represents the targeted eye condition, according to the legend. The dashed line is the publication year mean. VI, vision impairment

Who is conducting the research?

This multi-county work appears to be driven by institutions outside Pacific Island Countries, most frequently authors affiliated with institutions in Aotearoa, New Zealand. Only nine publications (45%) had an author affiliated with institutions in Pacific Island Countries (among which the Solomon Islands was the most frequent country of affiliation). Most publications did not report a funder, but when disclosed, the most frequent funders are the Fred Hollows Foundation Australia and the United stated Agency for International Development (USAID).

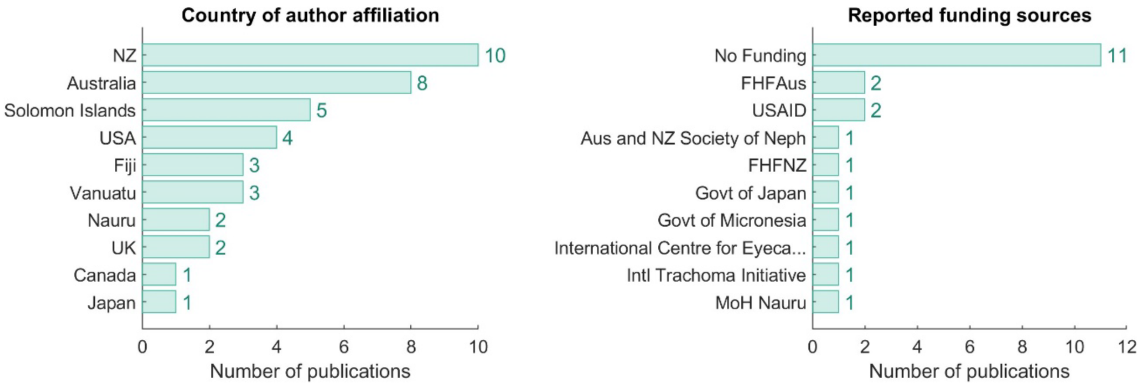

Figure 2. Country of author affiliation and disclosed funding sources. For each subplot, up to the top 10 are displayed (note that each study can have multiple affiliations and funding sources).

Papua New Guinea

What was the research about?

There were 61 publications that included PNG, and 52 specifically about PNG. The focus of the PNG specific research has been on less commonly researched eye conditions (coded at 'Other condition' in the figure below). These conditions include tumours/growths (n=3), ocular trauma (n=3), ocular leprosy (n=2), and a host of rare conditions. Almost half of these publications were describing service-based outcomes (n=24, 46%), and 6 additional publications are case publications. There were five publications that aimed to assess prevalence in population-based samples. Of these, the most common focus was vision impairment and its causes. The research in PNG has shifted from a focus descriptions of patients accessing services for less common conditions towards more robust analysis of vision impairment and it's causes.

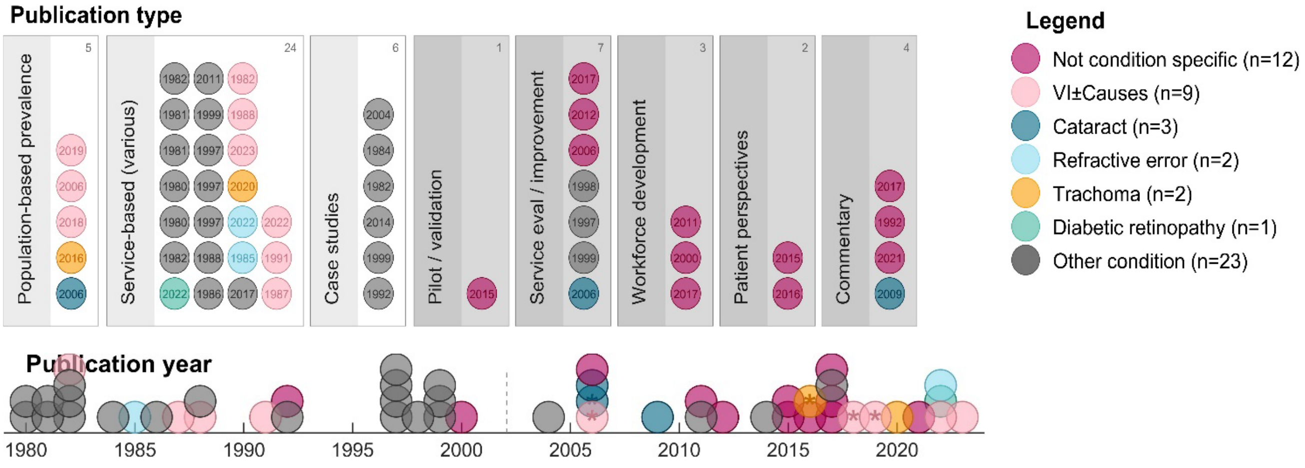

Figure 3. Publication type (upper panel) and timeline (lower panel). Each publication is represented by a dot, including study ID. Colour represents the targeted eye condition, according to the legend. The dashed line is the publication year mean. VI, vision impairment.

Who is conducting the research?

For publications conducted only in PNG, 45 publications (87%) had at least one author affiliated with institutions in Pacific Island Countries, and 22 publications had no external author affiliations. Accordingly, the most common author affiliation was PNG. Most publications did not report a funder, but when disclosed, the most frequent funder of this research has been Fred Hollows Foundation New Zealand.

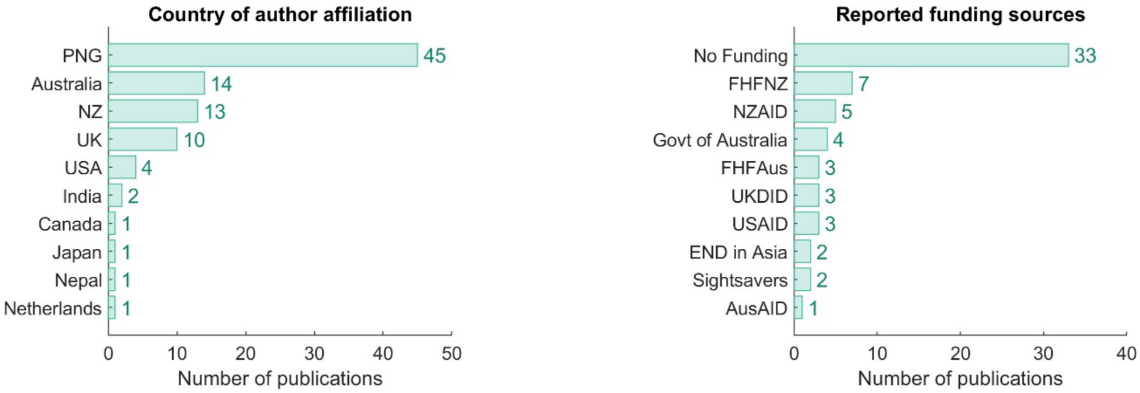

Figure 4. Country of author affiliation and disclosed funding sources. For each subplot, up to the top 10 are displayed (note that each study can have multiple affiliations and funding sources).

Fiji

What was the research about?

There were 46 publications that included Fiji, and 33 specifically about Fiji. Among the 33 publications specifically about Fiji, the most commonly targeted eye condition has been diabetic retinopathy (n=11, 33%). The most common publication type has been population-based prevalence publications (n=9, 27%). The focus of these prevalence studies has been varied, including trachoma (n=3), and diabetic retinopathy (n=2). Most of the research about eye health and eye health services in Fiji has been published since 2005, several reporting on the Fiji Eye Health Study 2009.

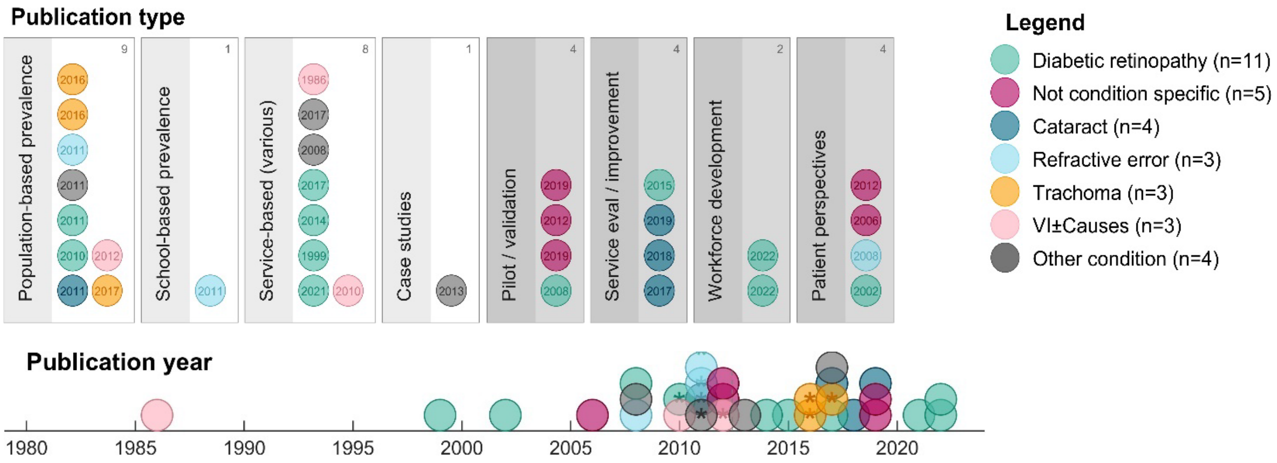

Figure 5. Publication type (upper panel) and timeline (lower panel). Each publication is represented by a dot, including study ID. Colour represents the targeted eye condition, according to the legend. The dashed line is the publication year mean. VI, vision impairment

Who is conducting the research?

For publications conducted only in Fiji, 23 publications (70%) had at least one author affiliated with institutions in Pacific Island Countries, and the most common author affiliation has been Fiji. Although most publications did not report funding, the most frequently reported funders have been AusAID and the Fred Hollows Foundation New Zealand.

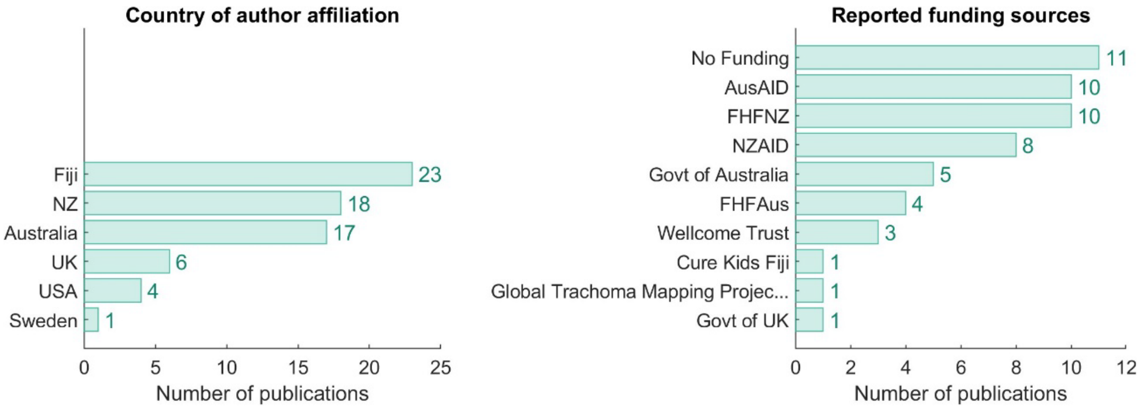

Figure 6. Country of author affiliation and disclosed funding sources. For each subplot, up to the top 10 are displayed (note that each study can have multiple affiliations and funding sources).

Vanuatu

What was the research about?

There were 35 publications that included Vanuatu, and 20 specifically about Vanuatu. Among the 20 publications specifically about Vanuatu, the most commonly targeted eye condition has been refractive error (n=7, 37%), including school-based prevalence publications mostly done in the 1980s. There were two more recent publications that aimed to assess prevalence in wider population-based samples. These publications were about trachoma (in 2017) and general vision impairment (in 1992).

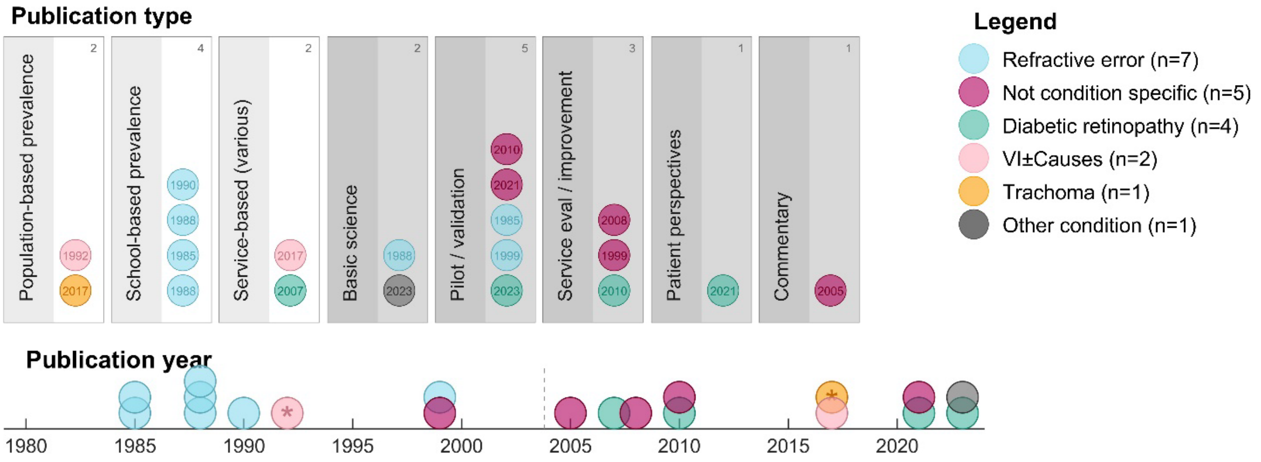

Figure 7. Publication type (upper panel) and timeline (lower panel). Each publication is represented by a dot, including study ID. Colour represents the targeted eye condition, according to the legend. The dashed line is the publication year mean. VI, vision impairment

Who is conducting the research?

For publications conducted only in Vanuatu, nine publications (45%) had at least one author affiliated with institutions in Pacific Island Countries. USA and Vanuatu are the most common countries of author affiliation. Most publications do not mention specific funding. When reported, the most frequent funder of this research has been the government of New Zealand.

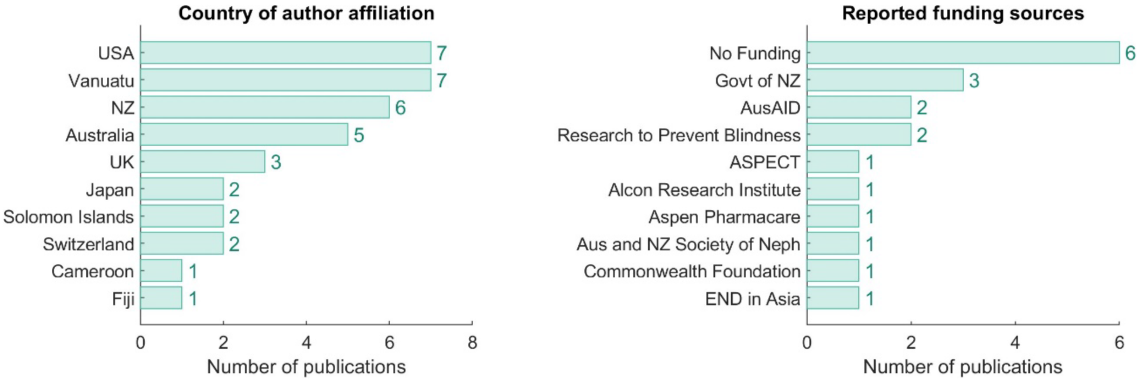

Figure 8. Country of author affiliation and disclosed funding sources. For each subplot, up to the top 10 are displayed (note that each study can have multiple affiliations and funding sources).

Solomon Islands

What was the research about?

There were 23 publications that included Solomon Islands, and nine specifically about Solomon Islands. These nine publications have been relatively recent, and dominated by trachoma research, including population-based prevalence publications, and other supporting work about molecular basis of trachoma and potential use of mass drug administration.

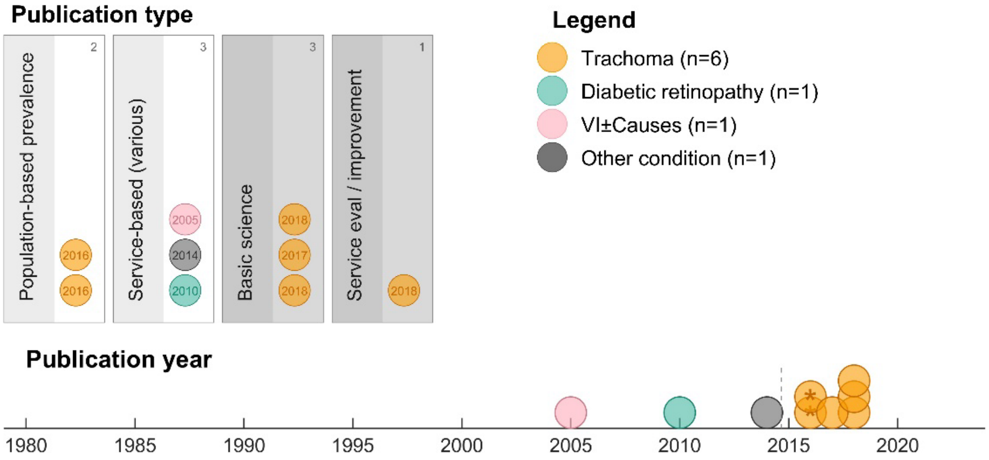

Figure 9. Publication type (upper panel) and timeline (lower panel). Each publication is represented by a dot, including study ID. Colour represents the targeted eye condition, according to the legend. The dashed line is the publication year mean. VI, vision impairment

Who is conducting the research?

For publications conducted only in Solomon Islands, eight publications (89%) had at least one author affiliated with institutions in Pacific Island Countries (with Solomon Island being the most frequent country affiliation), but there were no publications where all the authors were affiliated with institutions in the Solomon Islands. The trachoma work listed many funders (often embedded), with much of the support coming from organizations based in the United Kingdon (UK).

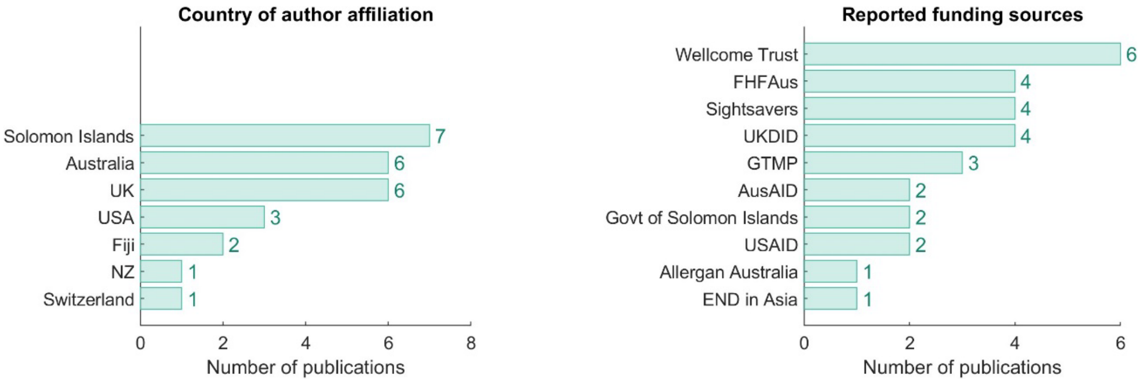

Figure 10. Country of author affiliation and disclosed funding sources. For each subplot, up to the top 10 are displayed (note that each study can have multiple affiliations and funding sources).

Federated states of Micronesia

What was the research about?

There were eight publications that included Federated states of Micronesia, and five specifically about Federated states of Micronesia. All of these publications were on less common conditions. Two publications were about a rare form of complete colour blindness found in Pingelap, called achromatopsia. Both of these focused on the genetic basis of the condition. There was an additional publication seeking to understand the genetic basis of Leber's congenital amaurosis (LCA). Micro-ophthalmia and vitamin A deficiency were also described for patients accessing service-based activities.

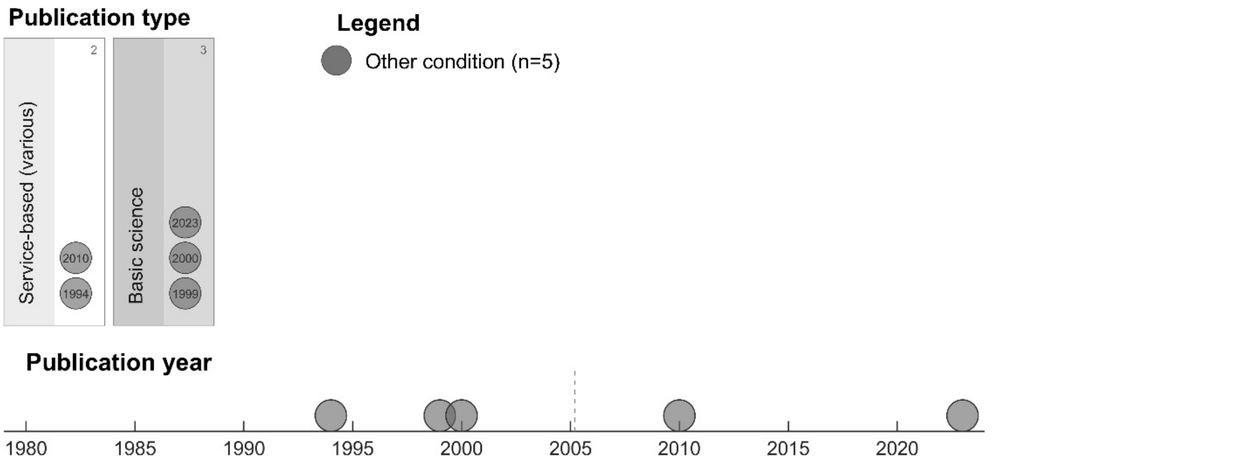

Figure 11. Publication type (upper panel) and timeline (lower panel). Each publication is represented by a dot, including study ID. Colour represents the targeted eye condition, according to the legend. The dashed line is the publication year mean. VI, vision impairment

Who is conducting the research?

For publications conducted only in Federated states of Micronesia, two publications (33%) had at least one authors affiliated with institutions in Pacific Island Countries. No single funder was acknowledged more than others. The most common country of author affiliation was the USA, and the top funder the (NIH) and many of the smaller funding agencies supporting this work are also based in the USA.

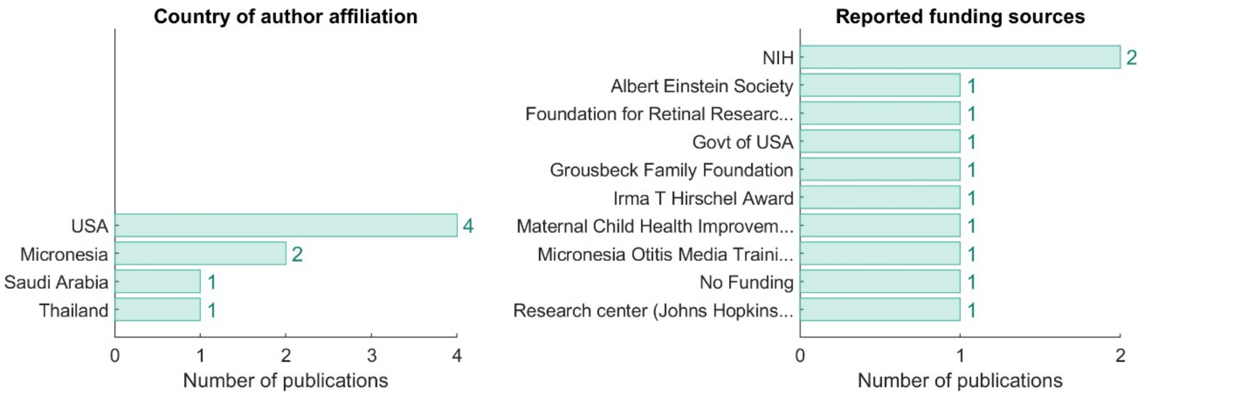

Figure 12. Country of author affiliation and disclosed funding sources. For each subplot, up to the top 10 are displayed (note that each study can have multiple affiliations and funding sources).

Marshall Islands

What was the research about?

There were nine publications that included Marshall Islands, and six specifically about Marshall Islands. Most of these six publications focused less commonly researched conditions, including ocular toxoplasmosis (n=1), tumours/growths (n=1), and vitamin A deficiency (n=1). Only a single publication estimated prevalence in population-based sample, this paper focused on general vision impairment, done in the 1980s. There have been no publications specifically about the Marshall Islands since 2005.

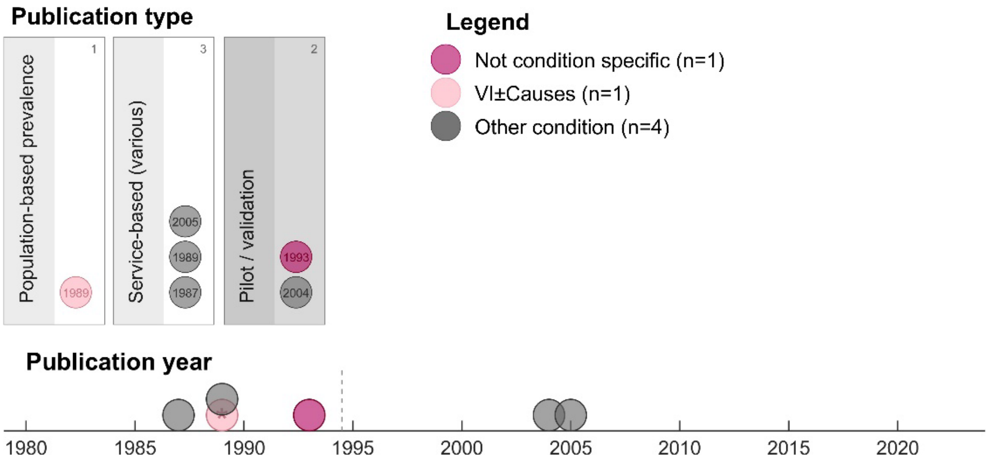

Figure 13. Publication type (upper panel) and timeline (lower panel). Each publication is represented by a dot, including study ID. Colour represents the targeted eye condition, according to the legend. The dashed line is the publication year mean. VI, vision impairment.

Who is conducting the research?

For publications conducted only in Marshall Islands, three publications (50%) had at least one author affiliated with institutions in Pacific Island Countries. The most common country of author affiliation was the USA, followed by the Marshall Islands. The most frequent funder of this research has been the Government of the USA.

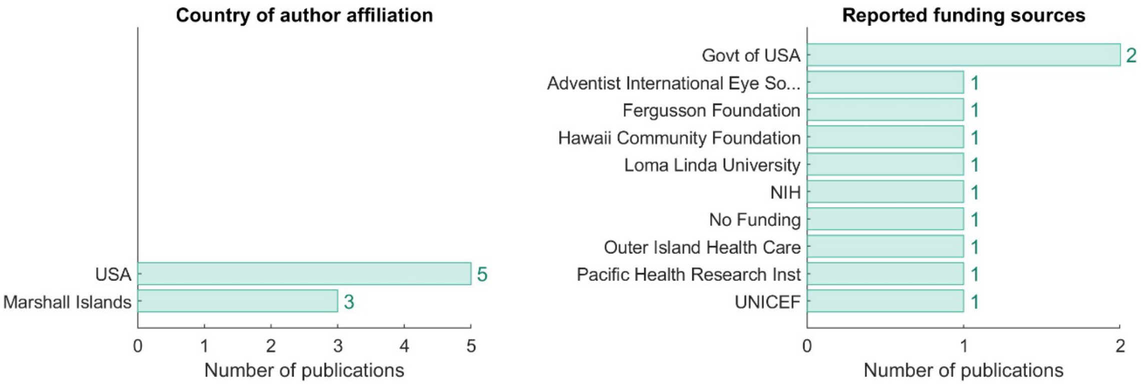

Figure 14. Country of author affiliation and disclosed funding sources. For each subplot, up to the top 10 are displayed (note that each study can have multiple affiliations and funding sources).

Nauru

What was the research about?

There were 12 publications that included Nauru, and five specifically about Nauru. All publications conducted specifically in Nauru are population-based prevalence publications. Note that several of the publications performed different analyses on data from a single study. Early publications (in the 1980s) targeted diabetic retinopathy (n=4, 67%), whereas the recent publication focused on the prevalence of trachoma.

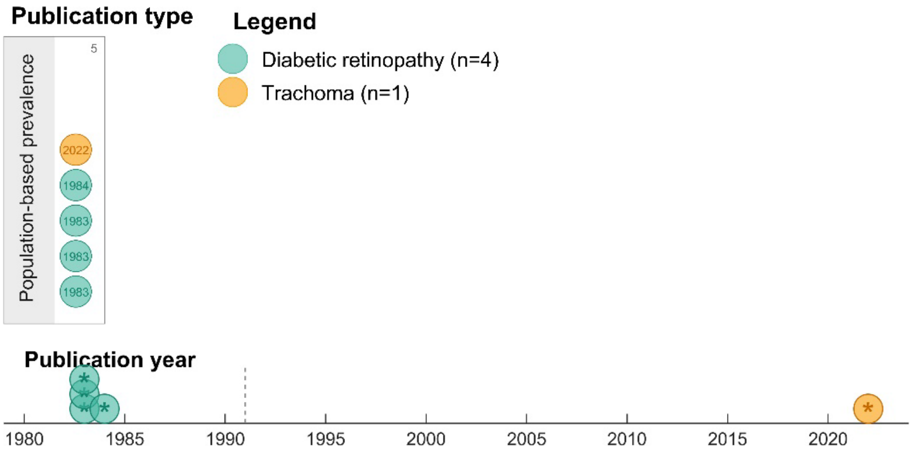

Figure 15. Publication type (upper panel) and timeline (lower panel). Each publication is represented by a dot, including study ID. Colour represents the targeted eye condition, according to the legend. The dashed line is the publication year mean. VI, vision impairment

Who is conducting the research?

For publications conducted only in Nauru, four publications (67%) had at least one author affiliated with institutions in Pacific Island Countries. The most common country of author affiliation was Australia. The early diabetic retinopathy research was funded by the NIH and WHO, whereas the newer research on trachoma is funded by wide a network of funders associated with global trachoma initiatives.

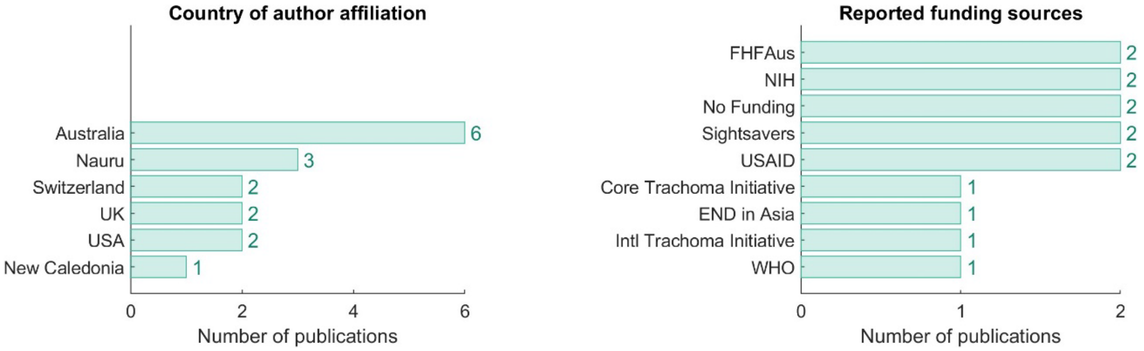

Figure 16. Country of author affiliation and disclosed funding sources. For each subplot, up to the top 10 are displayed (note that each study can have multiple affiliations and funding sources).

Samoa

What was the research about?

There were 14 publications that included Samoa, and six specifically about Samoa. The most common focus of these six publications in terms of eye condition has been diabetic retinopathy (n=3, 50%). The more recent work has been piloting screening services for diabetes and glaucoma and developing ways to monitor the impact of vision impairment in children. One 1995 publication aimed to assess the prevalence of diabetic retinopathy in a population-based sample.

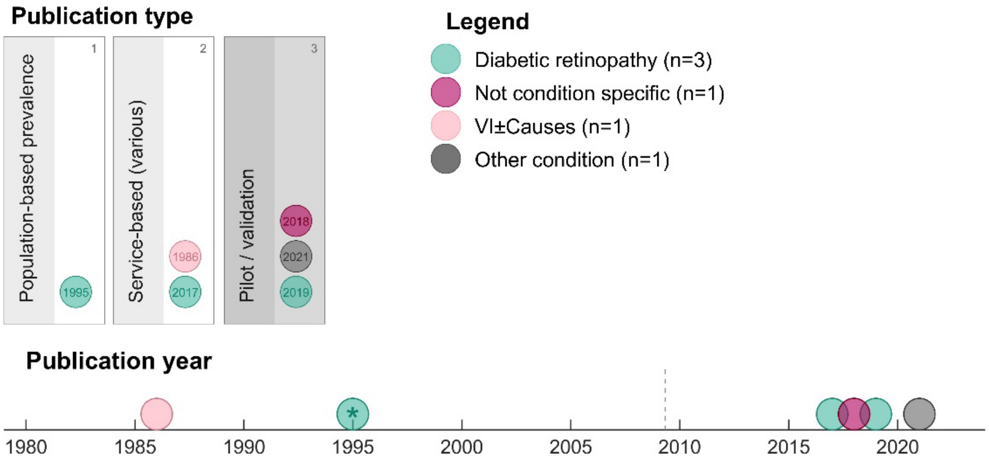

Figure 17. Publication type (upper panel) and timeline (lower panel). Each publication is represented by a dot, including study ID. Colour represents the targeted eye condition, according to the legend. The dashed line is the publication year mean. VI, vision impairment

Who is conducting the research?

For publications conducted only in Samoa, four publications (67%) had at least one authors affiliated with institutions in Pacific Island Countries. The most common country of author affiliation was the USA. Only two publications reported funding, each from a variety of funders.

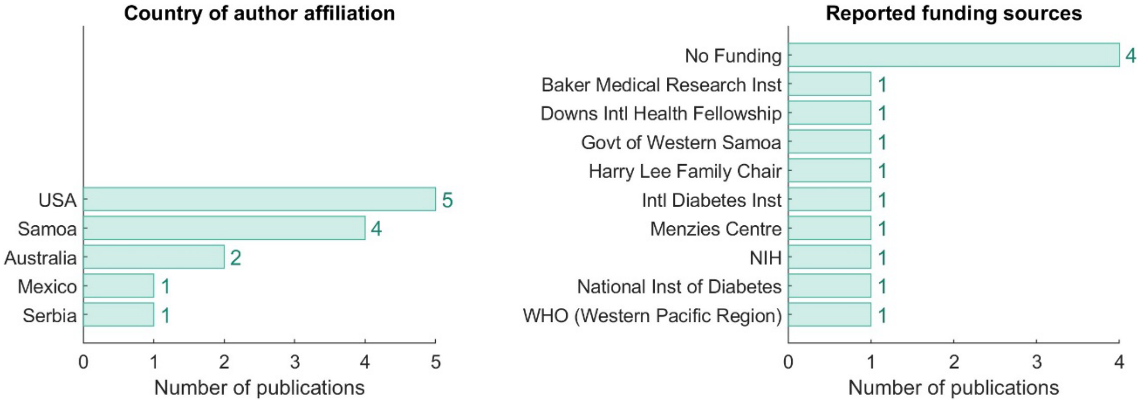

Figure 18. Country of author affiliation and disclosed funding sources. For each subplot, up to the top 10 are displayed (note that each study can have multiple affiliations and funding sources).

American Samoa

What was the research about?

There were six publications that included American Samoa, and five specifically about American Samoa. Most of these five publications describe specific outbreaks of conjunctivitis in the 1980s and 1990s (n=4, 80%), which we categorised as descriptions of services. There was one more recent (2011) publication about vision impairment and its causes. This study sampled from people attending outreach services in the island of Tu'a. Although only measuring those who attended outreach programmes, the sample represented 50% of the island's eligible population.

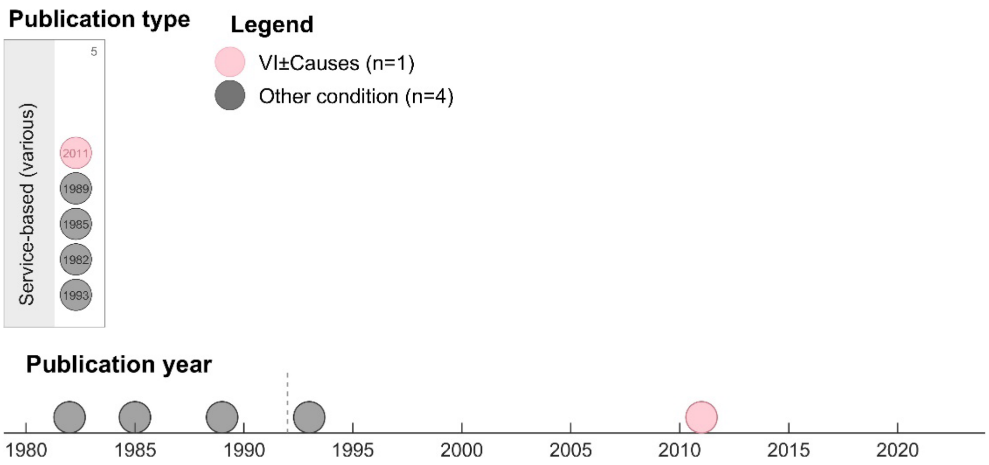

Figure 19. Publication type (upper panel) and timeline (lower panel). Each publication is represented by a dot, including study ID. Colour represents the targeted eye condition, according to the legend. The dashed line is the publication year mean. VI, vision impairment

Who is conducting the research?

Two publications (40%) had at least one author affiliated with institutions in Pacific Island Countries. The most common country of author affiliation was the USA. No single funder was acknowledged more than others, but when reported, funding was from the USA.

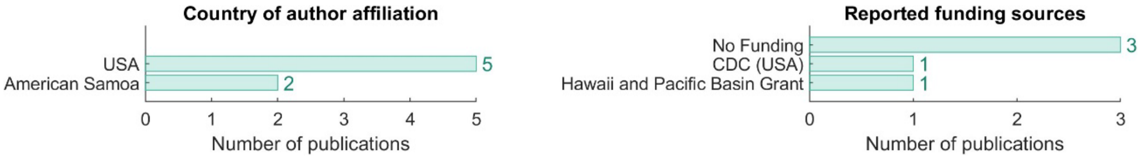

Figure 20. Country of author affiliation and disclosed funding sources. For each subplot, up to the top 10 are displayed (note that each study can have multiple affiliations and funding sources).

Guam

What was the research about?

There were six publications that included Guam, and five specifically about Guam. Among these five publications, most publications have been about a type of retinopathy thought to be connected to a rare neurological condition found in Guam (amyotrophic lateral sclerosis and parkinsonism-dementia complex 'ALS-PDC', n=4, 80%). Most of this work was done in the 1980s and 1990s. The 2015 publication was specifically exploring the connection between the retinopathy and ALS-PDC.

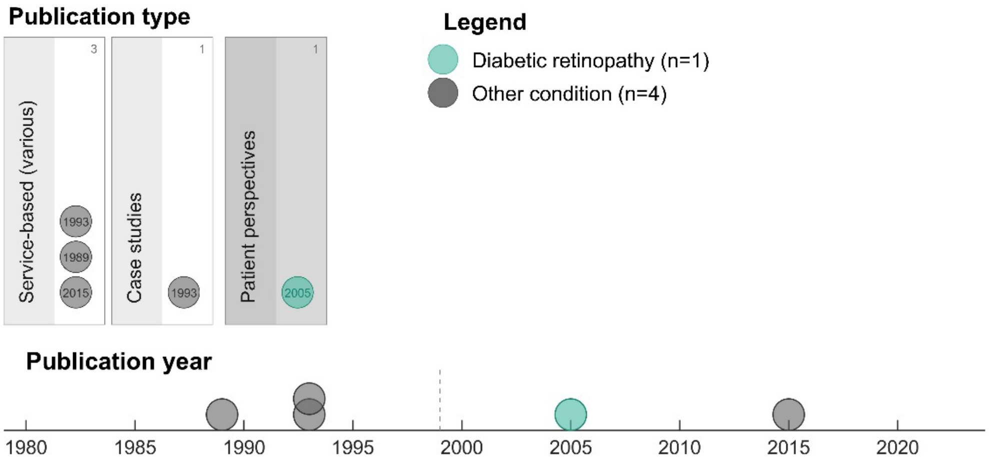

Figure 21. Publication type (upper panel) and timeline (lower panel). Each publication is represented by a dot, including study ID. Colour represents the targeted eye condition, according to the legend. The dashed line is the publication year mean. VI, vision impairment

Who is conducting the research?

For publications conducted only in Guam, each had at least one author affiliated with institutions in Guam (an unincorporated territory of the USA). No single funder was acknowledged more than others, but the bulk of funding was from North America.

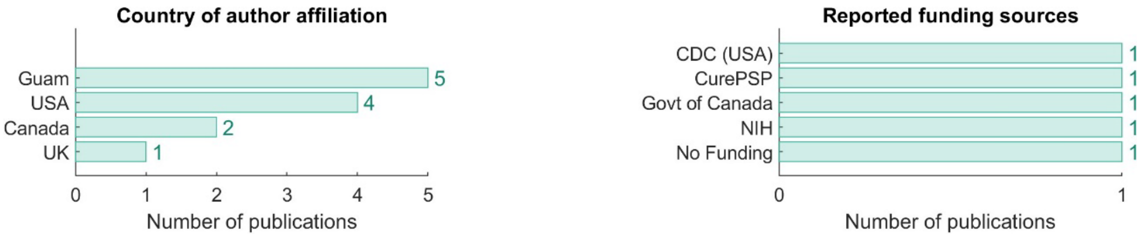

Figure 22. Country of author affiliation and disclosed funding sources. For each subplot, up to the top 10 are displayed (note that each study can have multiple affiliations and funding sources).

Kiribati

What was the research about?

There were 15 publications that included Kiribati, and five specifically about Kiribati. Among the five publications, the most common focus in terms of eye condition has been vitamin A deficiency (n=2, 40%). Population-based prevalence publications have been the most common publication type (n=2, 40%), one in the 1990's about vitamin A deficiency and two more recent publications about trachoma.

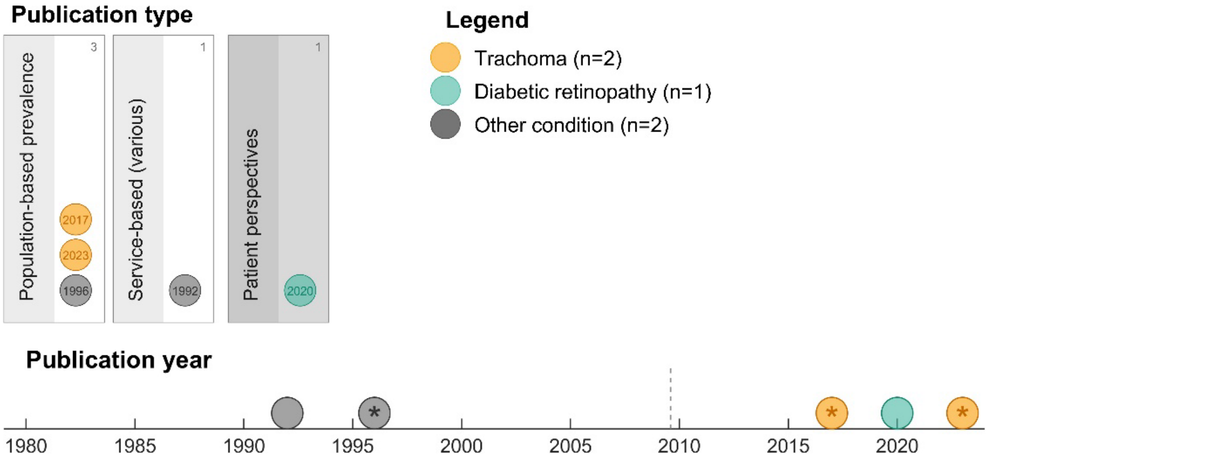

Figure 23. Publication type (upper panel) and timeline (lower panel). Each publication is represented by a dot, including study ID. Colour represents the targeted eye condition, according to the legend. The dashed line is the publication year mean. VI, vision impairment

Who is conducting the research?

For publications conducted only in Kiribati, four publications (80%) had at least one author affiliated with institutions in Pacific Island Countries, with Kiribati the most common. The most frequent funder of this research has been USAID.

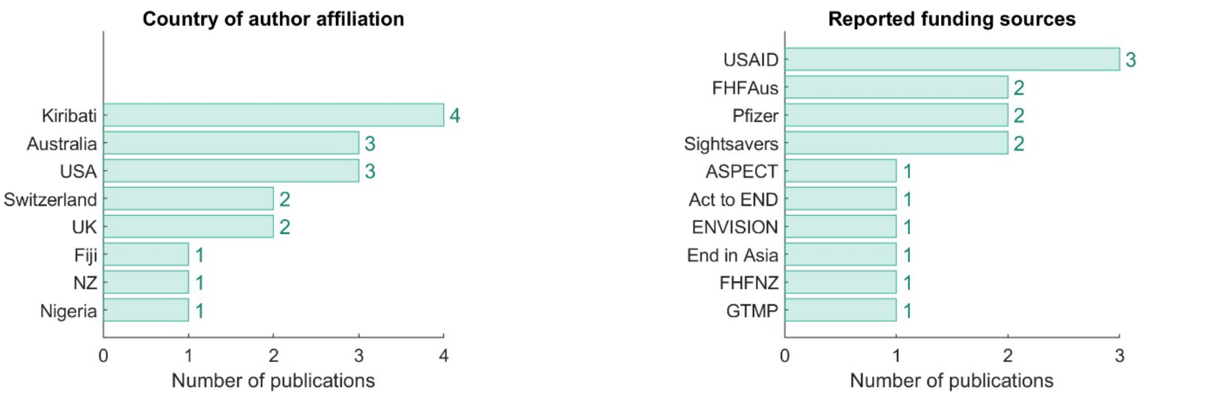

Figure 24. Country of author affiliation and disclosed funding sources. For each subplot, up to the top 10 are displayed (note that each study can have multiple affiliations and funding sources).

New Caledonia

Author	Category	Aim	Eye condition	Country of Authors	Funding Agencies
'Mancel 1999'	'Service-based various outcomes'	'To investigate the role of pathogenic leptospires as an aetiological agent of ocular leptospirosis.'	'Ocular leprosy'	'New Caledonia'	'No Funding'
'Tay 2007'	'Service-based various outcomes'	'To characterize the ophthalmic features and causes of vision loss in a cohort of Melanesians living in New Caledonia with nanophthalmos.'	'Micro or an-ophthalmia'	'Australia, New Caledonia, UK'	'Western Sydney Genetics Prgm, Children's Hospital (Westmead)'

Tonga

Author	Category	Aim	Eye condition	Country of Authors	Funding Agencies
'Newland 1994'	'Population-based prevalence'	'To obtain an accurate estimate of blindness and its causes in Tonga (population-based sample)'	'VI±Causes'	'Australia, Tonga'	'ASPECT, Australian International Development Assistance Bureau'
'Hamm 2020'	'School-based prevalence'	'To assess (1) the functional vision of children attending primary schools in Tonga and (2) how a new recognition acuity test (The Auckland Optotypes displayed on a tablet computer) compares to use of a standardised eye chart (school-based sample)'	'VI±Causes'	'NZ, Australia, Tonga, UK'	'NZAID, Cure Kids, NZOVRF, Robert Leitl Trust, Ranchhold Foundation'

Tuvalu

Author	Category	Aim	Eye condition	Country of Authors	Funding Agencies
'Leppaniemi 1990'	'Service evaluation / improvement'	'To estimate the number and types of surgeries in a 10 Year period, from medical records (clinical sample)'	'Cataract'	'Tuvalu'	'No Funding'
'Ramke 2006'	'Service evaluation / improvement'	'To determine the suitability of donated recycled spectacles for the Pacific nation of Tuvalu'	'Refractive error'	'Australia, NZ'	'NZOVRF, Govt of Australia'

Cook Islands

Author	Category	Aim	Eye condition	Country of Authors	Funding Agencies
"Heriot 1983"	"Population-based prevalence"	"To establish the prevalence of diabetes and its vascular complications among the Cook Islanders."	"VI±Causes"	"Australia, New Caledonia"	"No Funding"

Niue

Author	Category	Aim	Eye condition	Country of Authors	Funding Agencies
"Barker 1989"	"Population-based prevalence"	"To determine the health status and functional limitations of Niuean elderly (population-wide sample)"	"VI±Causes"	"New Zealand"	"Govt of NZ"

Northern Mariana Islands

Author	Category	Aim	Eye condition	Country of Authors	Funding Agencies
"Durand 1997"	"Service-based various outcomes"	"To determine the prevalence of diabetes in the Indigenous population of Commonwealth of the North Mariana Islands, from medical records"	"Diabetic retinopathy"	"USA, Northern Mariana Islands"	"No Funding"
