# Supplementary material for: Research about eye health and eye health services in Pacific Island Countries and Territories: a scoping review
Source: Lancet Reg Health West Pac. 2024 Jul 27;50:101152. doi: 10.1016/j.lanwpc.2024.101152 (PMC11332796; doi:10.1016/j.lanwpc.2024.101152)
Supplement: ANNEX 5_Hamm et al By Condition [file mmc3.pdf]

Annex 5: Overview of eye health research by targeted eye condition

Table of Contents

Not condition specific .... **Error! Bookmark not defined.**
What was the research about? iii
Who is conducting the research? iii
Diabetic retinopathy v
What was the research about? v
Who is conducting the research? v
VI±Causes iv
What was the research about? iv
Who is conducting the research? iv
Trachoma vii
What was the research about? vii
Who is conducting the research? vii
Refractive error ix
What was the research about? ix
Who is conducting the research? ix
Cataract x
What was the research about? x
Who is conducting the research? x
Conjunctivitis xi
What was the research about? xi
Who is conducting the research? xi
Vitamin A deficiency xii
What was the research about? xii
Who is conducting the research? xii
Ocular trauma xiii
What was the research about? xiii
Who is conducting the research? xiii
Tumors/growths xiv
What was the research about? xiv
Who is conducting the research? xiv
Retinopathy (ALS-PDC) xv
What was the research about? xv
Who is conducting the research? xv
Ocular leprosy xvi
Achromatopsia xvi
Glaucoma xvi
Meningitis xvi
Micro or an-ophthalmia xvii
Ptosis xvii
Anterior uveitis xvii

Cranial nerve palsy xvii
Large optic disks xvii
Leber congenital amaurosis xviii
Millipede burns xviii
Neurodevelopmental visual impairment xviii
Nystagmus xviii
Ocular angiostrongyliasis xviii
Ocular toxoplasmosis xviii
Pterygium xix
Quinine blindness xix
Retinopathy (malaria) xix

Publications that did not target a single eye condition

What was the research about?

If a publication did not target a specific eye condition, we separated them into those focused broadly on eye health services (described here) and those measuring vision impairment (with or without describing the causes, described in the next section).

These broad publications about eye health services included the piloting or validation of tools used to measure functional impact of vision impairment, publications focusing on workforce development, as well as commentaries summarising national level services. Although most took place in PNG, Fiji, and Vanuatu, nine were conducted across multiple countries. This broad research about eye health services has increased since the 1980.

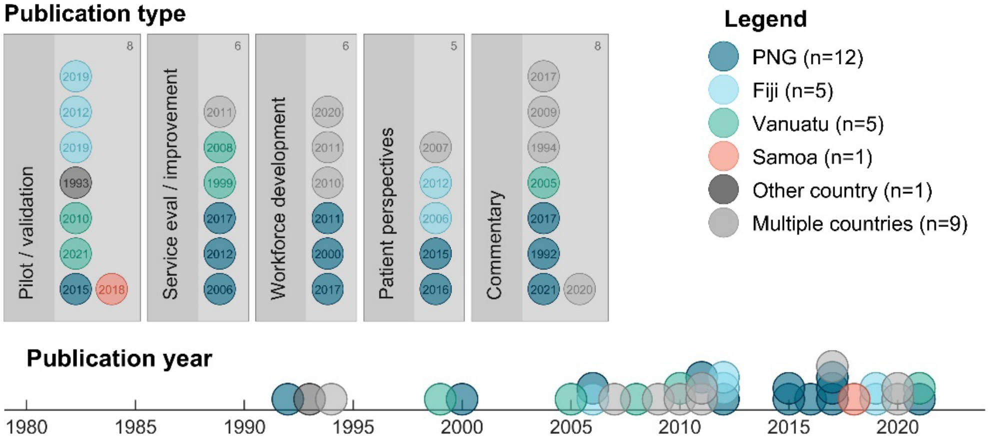

Figure 1. Publication type (upper panel) and timeline (lower panel). Each publication is represented by a dot, including study ID. Colour represents the country where the research was done, according to the legend.

Who is conducting the research?

Fourteen studies (42%) had at least one author affiliated with institutions in Pacific Island Country. The most frequent funder of this research has been Government of Australia, and most frequently affiliated countries were Australia and New Zealand.

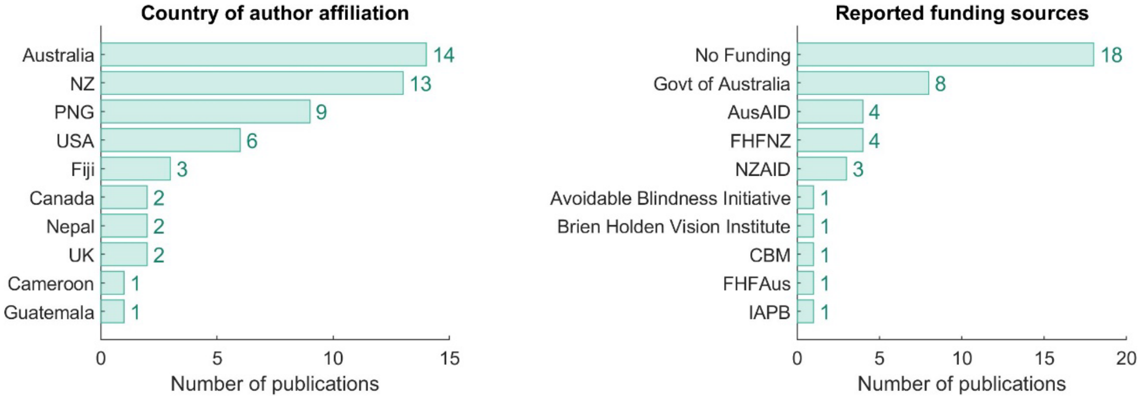

Figure 2. Country of author affiliation and disclosed funding sources. For each subplot, up to the top 10 are displayed (note that each study can have multiple affiliations and funding sources).

Vision impairment (± causes)

What was the research about?

Publications focused on vision impairment generally, tended to be either descriptions of patients accessing services, or population-based prevalence studies. Most studies were done in PNG. Of the nine studies that aimed to assess prevalence in population-based samples, only two were done in the last decade. Reported prevalence of blindness varied with sample age, ranging from 0.4% (>=6years, Cook Islands,1983) to 5.6% (>=50years, PNG, 2019). When reported, the most common cause of blindness was cataracts, and the most common cause of vision impairment was uncorrected refractive error.

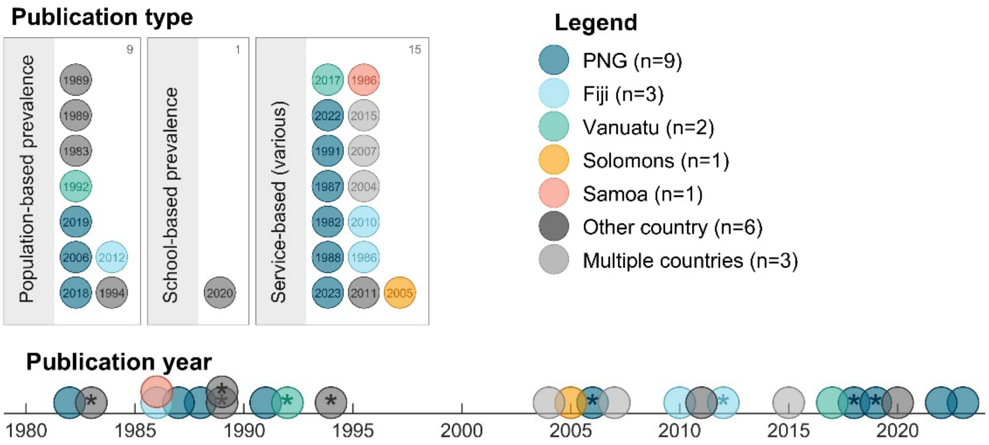

Figure 3. Publication type (upper panel) and timeline (lower panel). Each publication is represented by a dot, including study ID. Colour represents the country where the research was done, according to the legend.

Who is conducting the research?

Sixteen publications (64%) had at least one author affiliated with institutions in Pacific Island Countries. The most frequent funder of this research has been NZAID, followed AusAID and the Fred Hollows Foundation New Zealand.

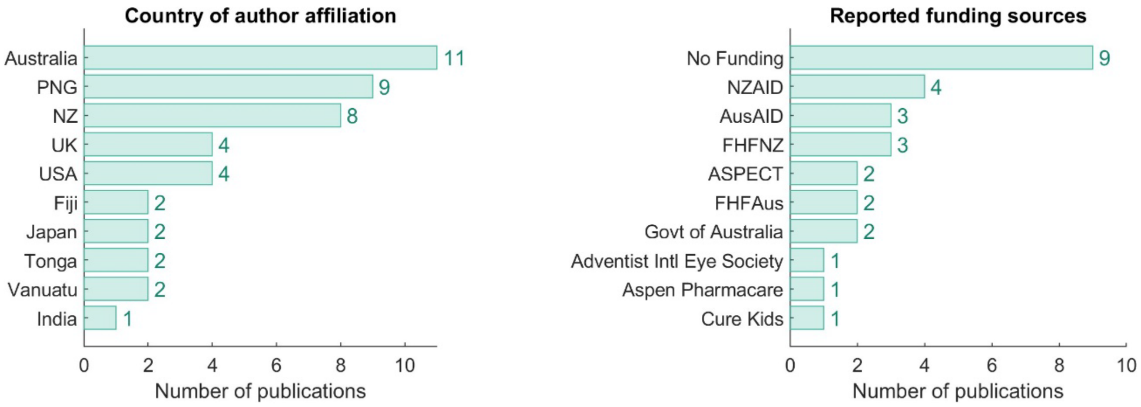

Figure 4. Country of author affiliation and disclosed funding sources. For each subplot, up to the top 10 are displayed (note that each study can have multiple affiliations and funding sources).

Diabetic retinopathy

What was the research about?

Research about diabetic retinopathy was always conducted with adults, covering a wide range of study types including population-based and service-based studies of eye health, and a variety of studies about eye health services (most frequently gathering patient perspectives about access to services and preventing the progression of retinopathy).

Seven publications described prevalence of diabetic retinopathy in population-based samples (four of these publications reported different components of data collected from a single study in Nauru in 1982, and two report different components of the Fiji Eye Health Survey 2009). Thresholds for diabetes and retinopathy varied between publications, with estimates of diabetic retinopathy ranging from 4-7%.

Overall, most diabetic retinopathy research has been done in Fiji. ‘Other’ countries with diabetic retinopathy research (not covered in the legend below) are Guam and Northern Marian Islands. The two studies across multiple countries were both conducted in Nauru and the Solomon Islands, and one included Vanuatu.

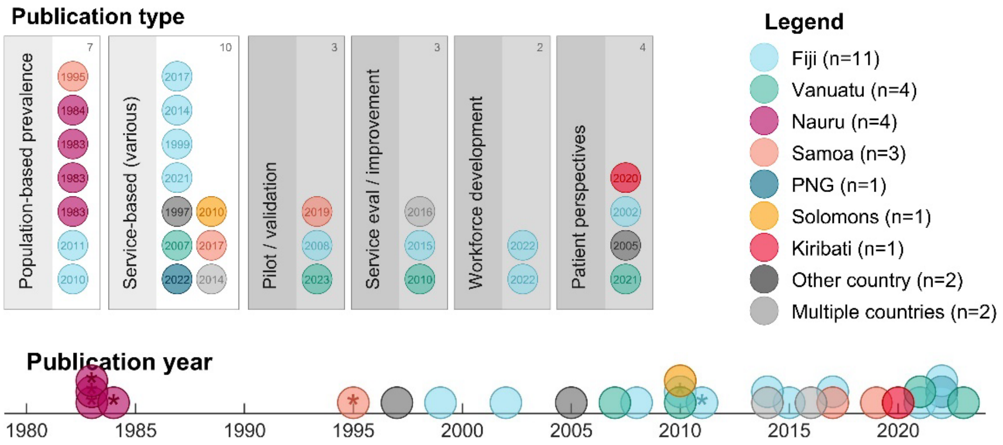

Figure 5. Publication type (upper panel) and timeline (lower panel). Each publication is represented by a dot, including study ID. Colour represents the country where the research was done, according to the legend.

Who is conducting the research?

Twenty-four studies (83%) had at least one author affiliated with institutions in Pacific Island Countries. The most frequent funder of this research has been the Fred Hollows Foundation New Zealand, with authors commonly affiliated with Australia, New Zealand and Fiji.

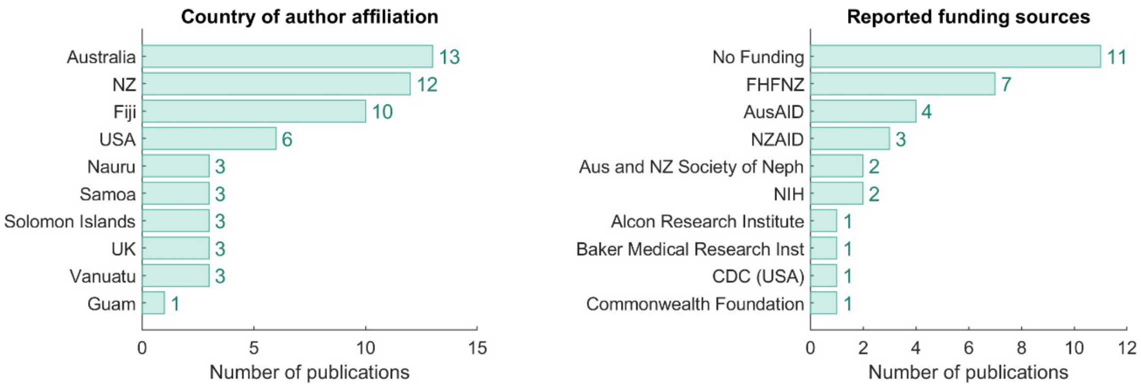

Figure 6. Country of author affiliation and disclosed funding sources. For each subplot, up to the top 10 are displayed (note that each study can have multiple affiliations and funding sources).

Trachoma

What was the research about?

Studies about trachoma tended to include all ages, with separate child and adult components in most studies. Most trachoma studies reported population-based prevalence (n=13), with the remainder exploring associated genetics and services improvement strategies to determine appropriateness of mass-drug administration. Of the 13 studies that aimed to assess prevalence in population-based samples, there were two or more publications Fiji, Solomon Islands, and Kiribati. Three studies were done across more than one country, each of these included Vanuatu, two included Solomon Islands, and two included Kiribati, and one included Nauru.

A 2009 study across several island nations suggested prevalence may be high, especially in Kiribati and the Solomon Islands. However, follow up studies generally showed low trachomatous trichiasis in those aged 15 years and above (most below the 0.2% WHO elimination threshold) despite higher rates of trachomatous inflammation in children.

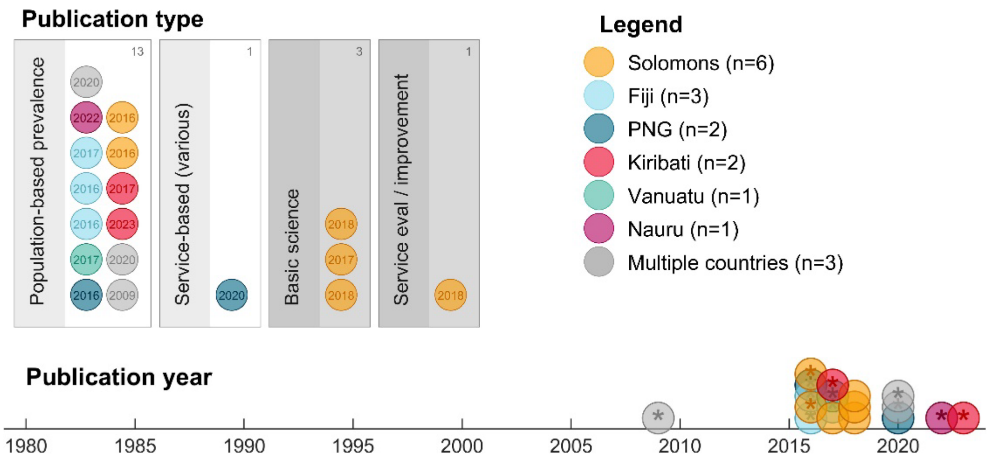

Figure 7. Publication type (upper panel) and timeline (lower panel). Each publication is represented by a dot, including study ID. Colour represents the country where the research was done, according to the legend.

Who is conducting the research?

All eighteen publications had at least one author affiliated with institutions in Pacific Island Countries. The most frequent author affiliation was within the United Kingdom. The most frequent funder of this research has been the Fred Hollows Foundation, Australia, and the Wellcome Trust, followed by Sightsavers, and other funders affiliated with the Global Trachoma Mapping Project.

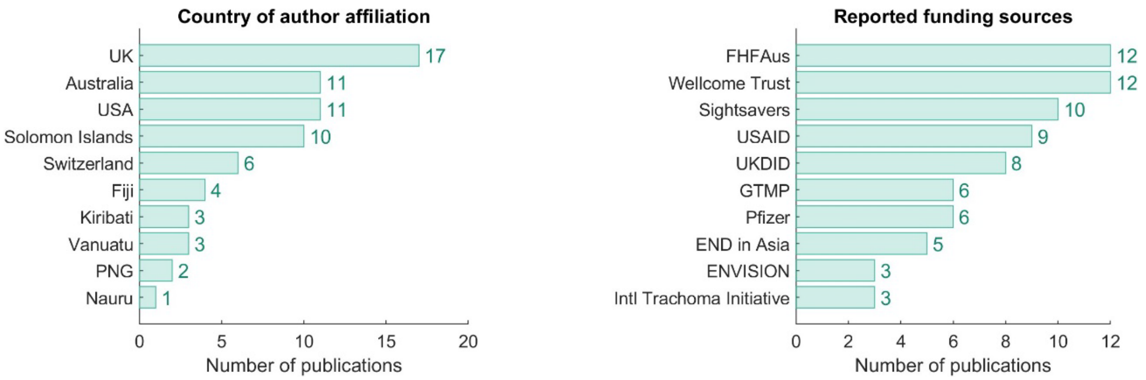

Figure 8. Country of author affiliation and disclosed funding sources. For each subplot, up to the top 10 are displayed (note that each study can have multiple affiliations and funding sources).

Refractive error

What was the research about?

Research targeting refractive error has mostly focused on prevalence in school-aged children. Most of these studies were done in Vanuatu in the 1980, and suggested rates of refractive error in children was low. The 2011 population-based study in Fiji reported the refractive error correction coverage was 68.3%, and presbyopia correction coverage was 42.2%. The most recent publication focused on refractive error was a retrospective review of ophthalmic clinic notes in PNG highlighting uncorrected refractive error as a significant cause of vision impairment in PNG. There was one publication conducted in Tuvalu about the appropriateness of recycled glasses, and one multi-country study, which summarised availability of refractive services and spectacle provision across eight countries.

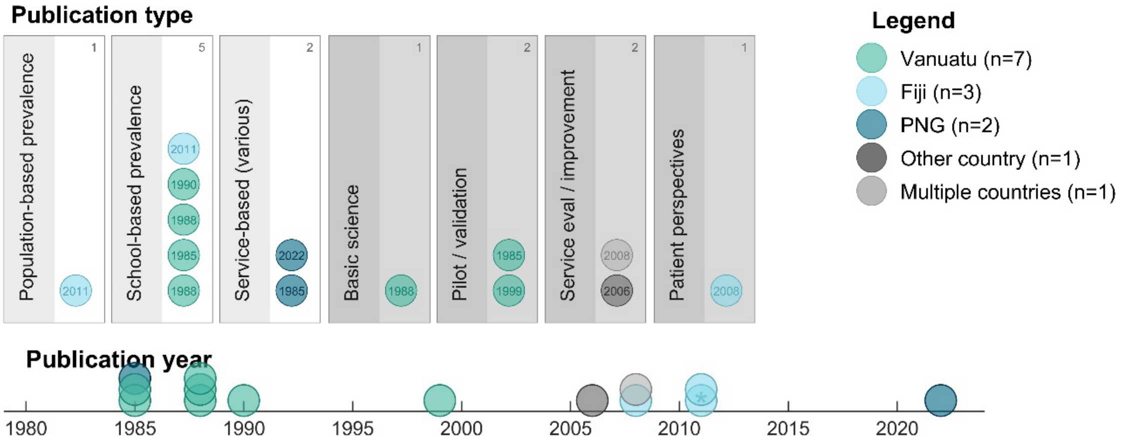

Figure 9. Publication type (upper panel) and timeline (lower panel). Each publication is represented by a dot, including study ID. Colour represents the country where the research was done, according to the legend.

Who is conducting the research?

Four studies (29%) had at least one author affiliated with institutions in Pacific Island Countries. The most frequent funder of this research has been Government of Aotearoa, New Zealand.

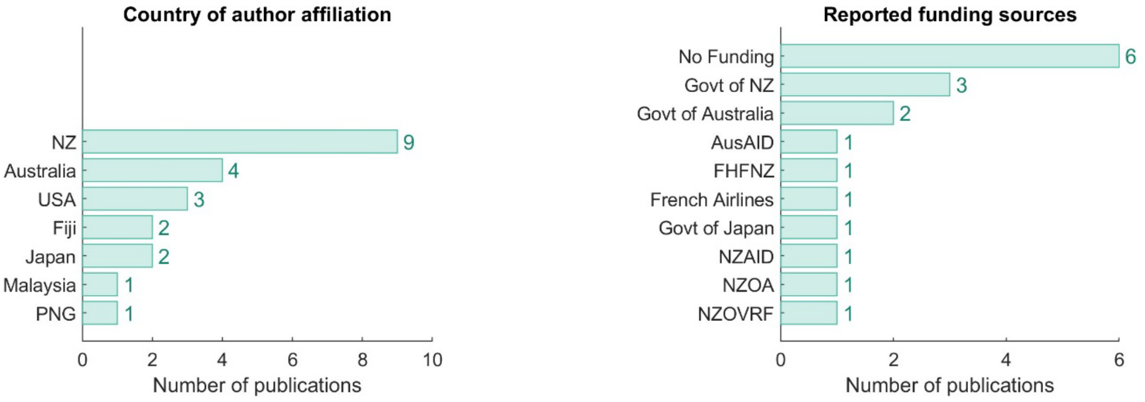

Figure 10. Country of author affiliation and disclosed funding sources. For each subplot, up to the top 10 are displayed (note that each study can have multiple affiliations and funding sources).

Cataract

What was the research about?

Research targeting cataracts has most commonly been about service evaluation and improvement, focused on how to optomise cataract removal surgery. Most of this work has been done in Fiji and PNG (the ‘other country’ where research was done was Tuvalu). The multi-country study tracked outcomes after cataract surgery done by a single resident ophthalmologist based in Fiji, doing outreach activities across five Pacific Island Countries.

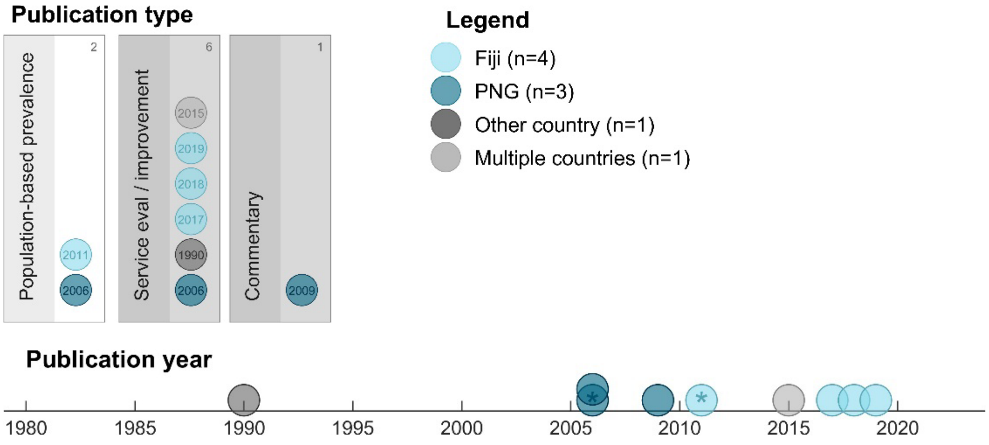

Figure 11. Publication type (upper panel) and timeline (lower panel). Each publication is represented by a dot, including study ID. Colour represents the country where the research was done, according to the legend.

Who is conducting the research?

All studies about cataract had at least one author affiliated with institutions in Pacific Island Countries. The most frequent funder of this research has been the Fred Hollows Foundation, New Zealand.

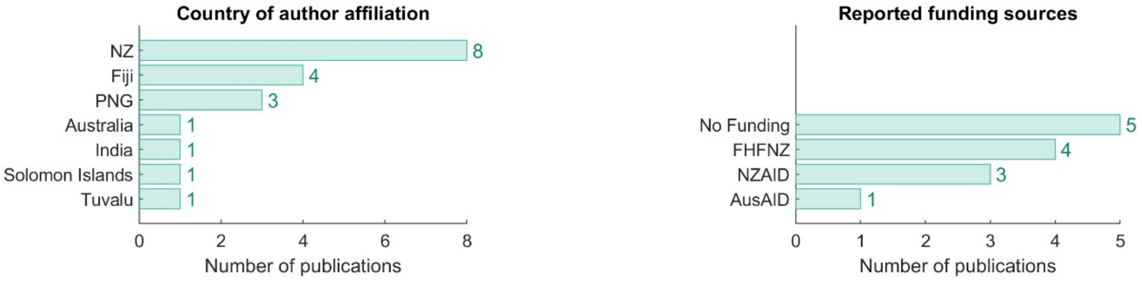

Figure 12. Country of author affiliation and disclosed funding sources. For each subplot, up to the top 10 are displayed (note that each study can have multiple affiliations and funding sources).

Conjunctivitis

What was the research about?

Almost all the studies about conjunctivitis were done in American Samoa (n=4). These were all describing outbreaks in the 1980s and early 1990s. The most recent study aimed to understand the viruses associated with conjunctivitis in Vanuatu.

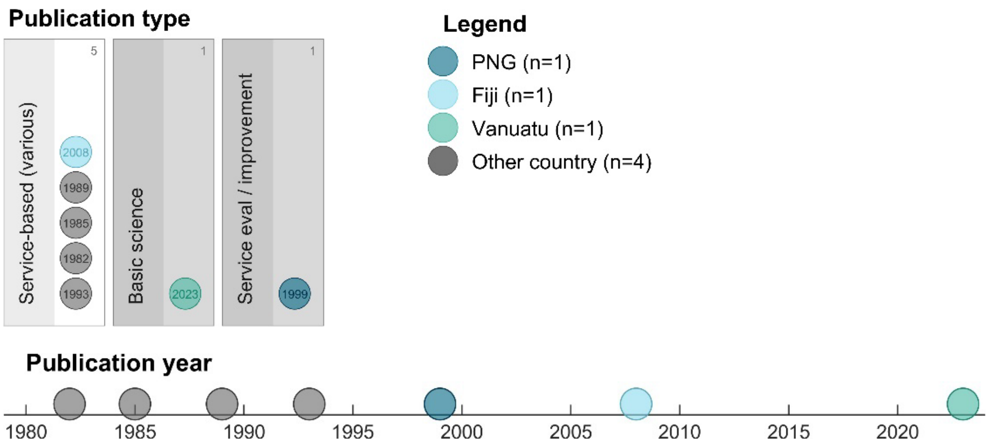

Figure 13. Publication type (upper panel) and timeline (lower panel). Each publication is represented by a dot, including study ID. Colour represents the country where the research was done, according to the legend.

Who is conducting the research?

Four studies (57%) had at least one author affiliated with institutions in Pacific Island Countries. Most studies had no funding.

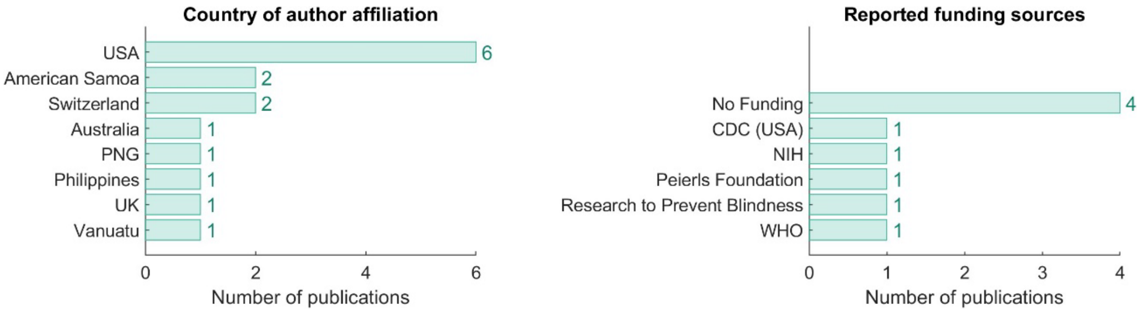

Figure 14. Country of author affiliation and disclosed funding sources. For each subplot, up to the top 10 are displayed (note that each study can have multiple affiliations and funding sources).

Vitamin A deficiency

What was the research about?

The most common category of study has been descriptions of patients accessing services (n=3, 50%). There were two studies that aimed to assess prevalence in population-based samples. Four of the six publications focused on children.

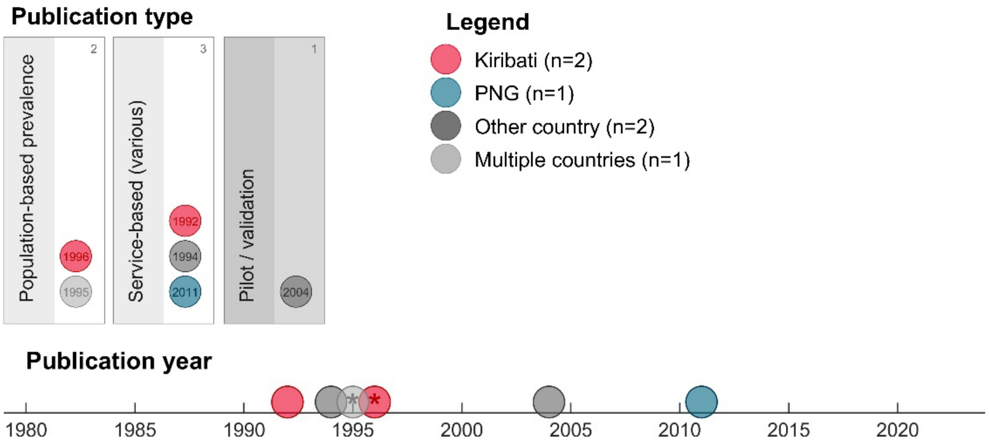

Figure 15. Publication type (upper panel) and timeline (lower panel). Each publication is represented by a dot, including study ID. Colour represents the country where the research was done, according to the legend. The dashed line is the publication year mean.

Who is conducting the research?

Four studies (67%) had at least one author affiliated with institutions in Pacific Island Countries. The most frequent funder of this research has been USAID.

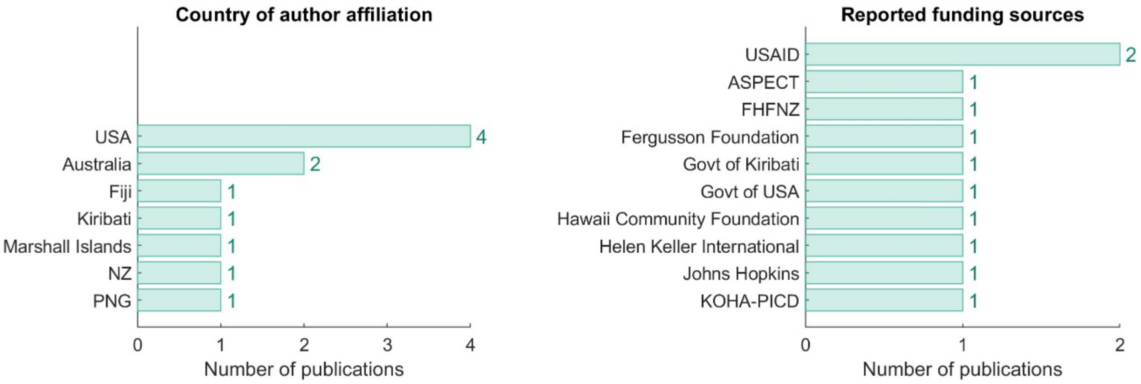

Figure 16. Country of author affiliation and disclosed funding sources. For each subplot, up to the top 10 are displayed (note that each study can have multiple affiliations and funding sources).

Ocular trauma

What was the research about?

The most common category of study has been descriptions of patients accessing services (n=3, 60%). There was one study that aimed to assess prevalence in population-based samples, in Fiji.

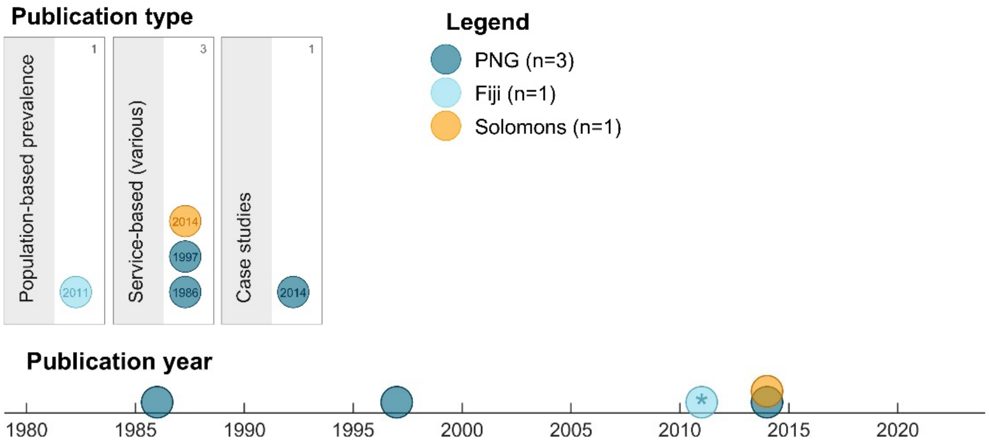

Figure 17. Publication type (upper panel) and timeline (lower panel). Each publication is represented by a dot, including study ID. Colour represents the country where the research was done, according to the legend.

Who is conducting the research?

All publication had at least one author affiliated with institutions in Pacific Island Countries. No single funder was acknowledged more than others.

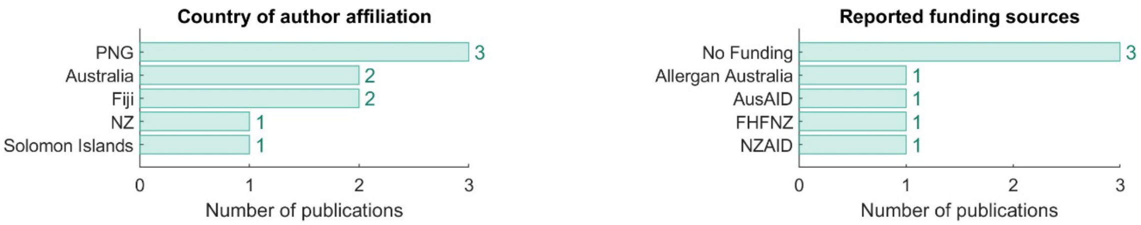

Figure 18. Country of author affiliation and disclosed funding sources. For each subplot, up to the top 10 are displayed (note that each study can have multiple affiliations and funding sources).

Tumours/growths

What was the research about?

The most common category of study has been descriptions of patients accessing services (n=3, 60%). Most of the research about tumours/growths has been done in PNG. About half of these focused on children.

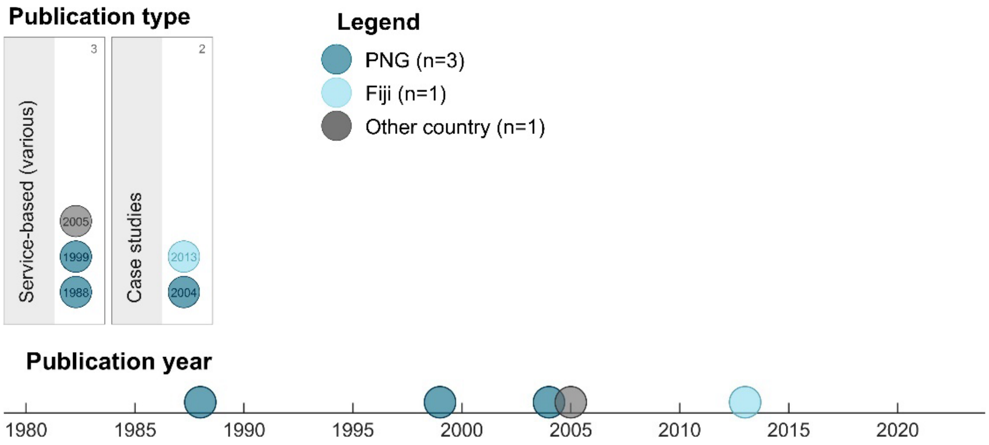

Figure 19. Publication type (upper panel) and timeline (lower panel). Each publication is represented by a dot, including study ID. Colour represents the country where the research was done, according to the legend.

Who is conducting the research?

All publications had at least one author affiliated with institutions in Pacific Island Countries. No single funder was acknowledged more than others.

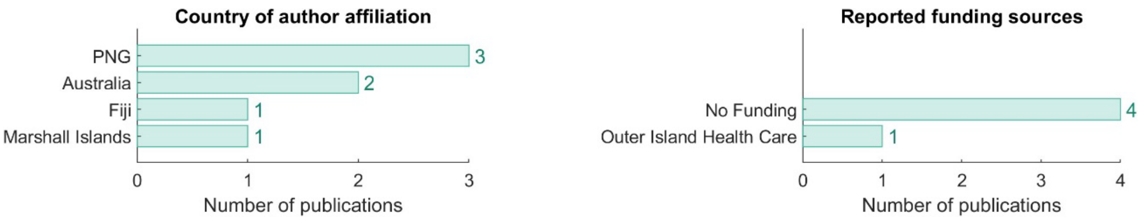

Figure 20. Country of author affiliation and disclosed funding sources. For each subplot, up to the top 10 are displayed (note that each study can have multiple affiliations and funding sources).

Retinopathy (ALS-PDC)

What was the research about?

The most common category of study has been descriptions of patients accessing services (n=3, 75%), all of these studies were done in Guam.

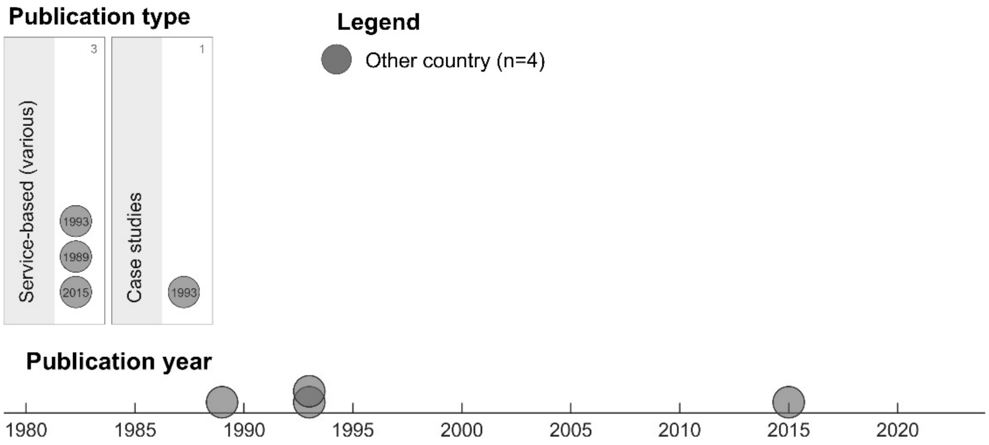

Figure 21. Publication type (upper panel) and timeline (lower panel). Each publication is represented by a dot, including study ID. Colour represents the country where the research was done, according to the legend.

Who is conducting the research?

All publications had at least one author affiliated with institutions in Pacific Island Countries. No single funder was acknowledged more than others.

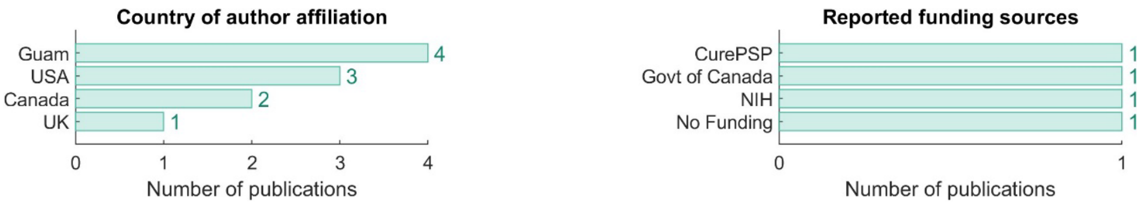

Figure 22. Country of author affiliation and disclosed funding sources. For each subplot, up to the top 10 are displayed (note that each study can have multiple affiliations and funding sources).

Ocular leprosy

Author	Category	Aim	Eye condition	Country of Authors	Funding Agencies
'Ree 1980'	'Service-based various outcomes'	'To describe ocular lesions in leprosy patients by type, duration and activity of the disease '	'Ocular leprosy'	'PNG'	'No Funding'
'Dethlefs 1981'	'Service-based various outcomes'	'To document the prevalence and type of ocular complications seen in leprosy patients in Port Moresby.'	'Ocular leprosy'	'PNG'	'No Funding'
'Mancel 1999'	'Service-based various outcomes'	'To investigate the role of pathogenic leptospires as an aetiological agent of ocular leptospirosis.'	'Ocular leprosy'	'New Caledonia'	'No Funding'

Achromatopsia

Author	Category	Aim	Eye condition	Country of Authors	Funding Agencies
'Winick 1999'	'Basic science'	'To obtain insights into the genetic basis of achromatopsia, as well as into the genetic history of Pingelapese kindreds with achromatopsia.'	'Achromatopsia'	'USA'	'Rockefeller University, NIH, Irma T Hirschel Award'
'Sundin 2000'	'Basic science'	'To describe the genetic basis of total colour blindness in Pingelap'	'Achromatopsia'	'USA'	'Research center (Johns Hopkins), Foundation for Retinal Research, Grousbeck Family Foundation, The Louise Sloan Trust, Wilmer Intramural Grant, Research to Prevent Blindness'

Glaucoma

Author	Category	Aim	Eye condition	Country of Authors	Funding Agencies
'LaMonica 2021'	'Pilot/validation of tool'	'To investigate factors that impact inter-rater agreement of glaucoma suspect optic disc status using a low-cost, handheld nonmydriatic fundus camera'	'Glaucoma'	'USA, Samoa'	'NIH, Downs Intl Health Fellowship, Yale School of Public Health, Harry Lee Family Chair'
'Dethlefs 1982'	'Service-based various outcomes'	'To describe patients with glaucoma who presented over a two year period at Port Moresby General Hospital, Papua New Guinea. '	'Glaucoma'	'Australia'	'No Funding'

Meningitis

Author	Category	Aim	Eye condition	Country of Authors	Funding Agencies
'Seaton 1997'	'Service evaluation / improvement'	'To compare treatment regimes for type of meingitis on visual loss (retrospctive)'	'Meningitis'	'PNG, UK'	'No Funding'
'Seaton 1997'	'Service-based various outcomes'	'To review ophthalmic findings in 82 immunocompetent patients and attempt to identify those features of the disease that predict an unfavourable visual outcome.'	'Meningitis'	'PNG, UK'	'No Funding'

Micro or an-ophthalmia

Author	Category	Aim	Eye condition	Country of Authors	Funding Agencies
'Tay 2007'	'Service-based various outcomes'	'To characterize the ophthalmic features and causes of visual loss in a cohort of Melanesians living in New Caledonia with nanophthalmos.'	'Micro or an-ophthalmia'	'Australia, New Caledonia, UK'	'Western Sydney Genetics Prgm, Children's Hospital (Westmead)'
'Yomai 2010'	'Service-based various outcomes'	'To explore potential putative genetic and environmental causes previously associated with rare congenital defects of eye development (microphthalmia and anophthalmia) in Chuuk, Federated States of Micronesia, where alarmingly high rates have been observed. The study will hopefully shed light on the prevention of these conditions.'	'Micro or an-ophthalmia'	'Micronesia'	'No Funding'

Ptosis

Author	Category	Aim	Eye condition	Country of Authors	Funding Agencies
'Scrimgeour 1984'	'Case study'	'To describe a case of ptosis (alongside wider issues)'	'Ptosis'	'PNG, Australia'	'No Funding'
'Gushchin 2017'	'Service-based various outcomes'	'To present the results of a high-volume oculoplastic surgical outreach in a remote region of Simbu Province in the Eastern Highlands of Papua New Guinea. The authors describe the clinical features and evaluation and treatment of a novel ptosis syndrome found in this area (outreach sample)'	'Ptosis'	'USA, Nepal'	'Monica Mercer'

Anterior uveitis

Author	Category	Aim	Eye condition	Country of Authors	Funding Agencies
"Dethlefs 1981"	"Service-based various outcomes"	"To describe case studies of acute anterior uveitis (iritis), presenting at clinic"	"Anterior uveitis"	"PNG"	"No Funding"

Cranial nerve palsy

Author	Category	Aim	Eye condition	Country of Authors	Funding Agencies
"Cheng 1999"	"Case study"	"To describe a case of a complete superior orbital fissure syndrome including the optic nerve"	"Cranial nerve palsy"	"PNG"	"No Funding"

Large optic disks

Author	Category	Aim	Eye condition	Country of Authors	Funding Agencies
"Maisel 1989"	"Service-based various outcomes"	"To compare the fundus photographs of a group of patients noted to have high cup/disk ratios and that of a selected group of control patients in order to prove that the optic disks that appeared on ophthalmoscopic examination to have high cup/disk ratios were actually larger than those optic disks judged clinically normal on ophthalmoscopy."	"Large optic disks"	"USA"	"Govt of USA"

Leber congenital amaurosis

Author	Category	Aim	Eye condition	Country of Authors	Funding Agencies
"Albakri 2023"	"Basic science"	"To determine the cause of Leber congenital amaurosis (LCA) in Chuuk state, Federated States of Micronesia (FSM). We describe the discovery of a novel frameshift pathogenic variant in CRB1, as the cause of LCA in Chuuk, Micronesia, a geographically isolated population."	"Leber congenital amaurosis"	"USA, Saudi Arabia, Thailand, Micronesia"	"The Foerderer Fund, Robison Harley Endowed Chair, NIH, Albert Einstein Society"

Millipede burns

Author	Category	Aim	Eye condition	Country of Authors	Funding Agencies
"Hudson 1997"	"Service-based various outcomes"	"To determine the frequency and severity of eye conditions due to 'burns' from these millipedes, and to review management."	"Millipede burns"	"PNG"	"No Funding"

Neurodevelopmental visual impairment

Author	Category	Aim	Eye condition	Country of Authors	Funding Agencies
"Milner 2017"	"Service-based various outcomes"	"To assess early childhood neurodevelopmental and health outcomes for a cohort of high-risk neonates, defined on the basis of NICU admission, relative to control healthy, term neonates in Fiji, specifically: 1. prevalence of moderate to severe NDI at approximately 2 years of age 2. prevalence of stunting, wasting, anaemia, other health morbidity, immunisation rates and health service usage"	"Neurodevelopmental visual impairment"	"Australia, UK, Fiji"	"AusAID, Cure Kids Fiji"

Nystagmus

Author	Category	Aim	Eye condition	Country of Authors	Funding Agencies
"Hornabrook 1980"	"Service-based various outcomes"	"To understand nystagmus in what appears to be a unique form of albinism in a rural area"	"Nystagmus"	"PNG, UK"	"No Funding"

Ocular angiostrongyliasis

Author	Category	Aim	Eye condition	Country of Authors	Funding Agencies
"Scrimgeour 1982"	"Case study"	"To describe a case of an intraocular nematode infestation"	"Ocular angiostrongyliasis"	"PNG"	"No Funding"

Ocular toxoplasmosis

Author	Category	Aim	Eye condition	Country of Authors	Funding Agencies
"Adams 1987"	"Service-based various outcomes"	"To determine if radiation exposure may have produced long term immunosuppression which might place the exposed population at greater risk for clinical illness due to toxoplasmosis. A second goal of the investigation was to evaluate the prevalence of Toxoplasma	"Ocular toxoplasmosis"	"USA"	"Govt of USA"

Author	Category	Aim	Eye condition	Country of Authors	Funding Agencies
		seropositivity and the prevalence and incidence of suspected Toxoplasma retinochoroiditis in a larger population of Marshallese which included unexposed individuals."			

Pterygium

Author	Category	Aim	Eye condition	Country of Authors	Funding Agencies
"Verma 1998"	"Service evaluation / improvement"	"To compare the results of a single intraoperative application of 0.02% mitomycin C solution with a similar group of 65 patients in which the drug was not used."	"Pterygium"	"PNG"	"No Funding"

Quinine blindness

Author	Category	Aim	Eye condition	Country of Authors	Funding Agencies
"Naraqi 1992"	"Case study"	"To present a case report on quinine blindness and review the management"	"Quinine blindness"	"PNG"	"No Funding"

Retinopathy (malaria)

Author	Category	Aim	Eye condition	Country of Authors	Funding Agencies
"Davis 1982"	"Service-based various outcomes"	"To determine the frequency of retinal hemorrhages in acuity malaria of al types and to examine various clinical and hematological indices in relation to their genesis"	"Retinopathy (malaria)"	"PNG"	"No Funding"
